# Transformation of lung cancer patient education paradigm driven by large language models: a multidimensional performance study

**DOI:** 10.1097/JS9.0000000000003503

**Published:** 2025-09-22

**Authors:** Chang Song, Chun-Yan Zhao, Tong Yang, Ren-Hao Liu, Zhi-Jun Chen, Mei-Song Liu, Qing-Dong Zhu, Jing-Song Chen

**Affiliations:** a Department of Gastroenterology, Hepu County People’s Hospital, Beihai, Guangxi, China; b Guangxi Medical University, Nanning, Guangxi, China; c Department of Rehabilitation, Hepu County People’s Hospital, Beihai, Guangxi, China; d Department of Information, Hepu County People’s Hospital, Beihai, Guangxi, China; e Administration Office, Hepu County People’s Hospital, Beihai, Guangxi, China; f Department of Tuberculosis, The Fourth People’s Hospital of Nanning, Nanning, Guangxi, China

Lung cancer remains a major global public health challenge, ranking first in overall cancer-related mortality[1]. Despite recent advancements in precision medicine, such as targeted therapy and immunotherapy, which have expanded diagnostic and treatment options, patients continue to face numerous barriers in managing their disease. Notably, the lack of disease-related knowledge, insufficient treatment adherence, and increased psychological burden are particularly prominent. Studies have shown that stigma associated with lung cancer can delay patients’ medical consultations, thereby impeding early diagnosis and significantly diminishing treatment efficacy[2]. Health education and consultation services play a crucial role in lung cancer prevention and management[3]. As the leading cause of cancer mortality worldwide, lung cancer presents critical challenges in multidisciplinary collaboration and information integration, making it a priority scenario for Large language models (LLMs) applications. The disease’s prominent psychosocial needs (e.g., the high prevalence of prognostic anxiety) provide a unique window for evaluating clinical empathy capabilities. Unlike the single-modality-dominated diagnosis of breast and prostate cancers, lung cancer relies on multimodal fusion analysis incorporating imaging, pathology, and genomics, which better demonstrates the core value of LLMs in processing complex medical information. However, traditional educational models are constrained by limited human resources and high time costs, making it difficult to deliver personalized and continuous care. LLMs, a major breakthrough in artificial intelligence, offer substantial promise in the health care sector due to their advanced natural language processing capabilities and extensive knowledge bases. Research has demonstrated that these technologies exhibit significant potential in various domains, including medical knowledge querying, clinical decision support, and patient education. In the field of oncology, LLMs have been employed to provide patients with personalized cancer information support^[4,5]^. This integrated model, combining medical expertise with psychological assistance, has the potential to improve patient education, increase treatment compliance, and enhance overall disease management. However, it is crucial to acknowledge that LLMs exhibit notable limitations in clinical applications, including, but not limited to, challenges in ensuring information accuracy, algorithmic bias issues, deficiencies in emotional empathy, and ethical concerns[6]. Accordingly, this study aims to systematically evaluate the multidimensional performance of leading LLMs in lung cancer patients, education, providing empirical evidence and actionable insights for the integration of artificial intelligence (AI) technologies into lung cancer prevention and treatment systems.

This study employed a multidimensional approach to construct a disease-related question bank by integrating multi-source data from online medical forums, clinical experience, and patient needs. Through the analysis of concerns raised by over 200 lung cancer patients, combined with electronic medical records from both outpatient and inpatient settings, we systematically identified common issues encountered by patients throughout the continuum of care. During the questionnaire design phase, the collected questions underwent dual screening by medical experts and patient representatives, followed by expert panel discussions to evaluate and optimize the clinical relevance and clarity of each item. Ultimately, a comprehensive questionnaire consisting of 33 items was developed (Supplemental Digital Content Table 1, available at http://links.lww.com/JS9/F178), encompassing five critical dimensions: basic knowledge, diagnosis, treatment, prognosis and nursing care, as well as clinical cases, thereby ensuring the scientific validity and practical utility of the instrument. For model evaluation, four mainstream LLMs, including ChatGPT-4o, Copilo, Gemini Advanced 2.0 Flash, and Claude 3.7 Sonnet were selected. Testing was conducted in March 2025, and all model interactions were recorded in full (Supplemental Digital Content Table 2, available at http://links.lww.com/JS9/F178). A five-point Likert scale was used for evaluation, with triple quality-control scoring performed independently by three clinical experts. The scoring protocol incorporated double-blind assessment, independent feedback, and result verification, with the final scores calculated as the average of the three expert ratings. Statistical analysis was performed using SPSS 21.0. Descriptive statistics (mean, standard deviation) were calculated, and scale reliability and inter-rater consistency were assessed through the Cronbach’s alpha coefficient and the intraclass correlation coefficient (ICC). One-way ANOVA was used to compare score differences, with *post hoc* tests selected and determined by the homogeneity of variance. A significance threshold of *P* < 0.05 was applied. The entire study adhered to contemporary standards of methodological rigor in AI-related research, following the “TITAN” framework proposed by Agha et al., which provides comprehensive guidelines for ensuring transparency in AI studies[7].

As shown in Supplemental Digital Content Table 3, available at http://links.lww.com/JS9/F178, the three raters demonstrated strong inter-rater reliability across all questions (ICC single = 0.844, ICC average = 0.942, Cronbach’s α = 0.942) and across different question types (*P* < 0.001), confirming the robustness of the evaluation. Model performance across the 33 questions is shown in Figure 1.

**Figure 1.**
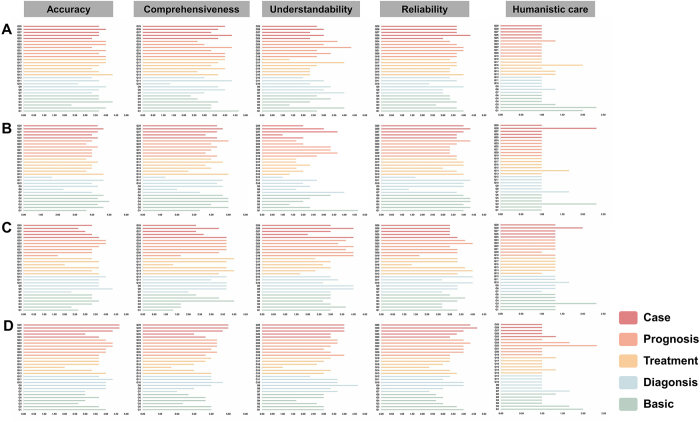
The performance of the four LLMs on various questions across different dimensions. (A) Chat GPT; (B) Claude; (C) Copilt; (D) Gemini.

Dimension-specific analysis revealed significant differences in all areas except for humanistic care (*P* < 0.05). Claude performed with the highest accuracy and comprehensiveness, while Coplit performed best in terms of understandability. All models performed poorly in the humanistic care dimension, with no significant differences between them (Table 1). *Post hoc* analysis further revealed that Claude outperformed the other models in accuracy and comprehensiveness (*P* < 0.001), but ranked lowest in understandability. In contrast, Coplit surpassed Claude in understandability but was significantly less accurate and reliable than Gemini and Claude (*P* < 0.05). ChatGPT-4o demonstrated moderate performance in both reliability and understandability. These results quantify the variation in model capabilities and provide a basis for informed model selection (Supplemental Digital Content Table 4, available at http://links.lww.com/JS9/F178). However, despite the slight advantage of the Claude model in terms of accuracy, the clinical significance of this difference still needs further verification. Subsequent research should explore the performance differences of the model in different application scenarios to more comprehensively evaluate its practical value.

**Table 1:** Scores of four LLMs in different dimensions

Different dimensions	LLM	Minimum	Maximum	Mean	SD	SE	F	*P* value
Accuracy*	ChatGPT	2.330	4.330	3.679	0.432	0.080	7.788	<0.001
	Claude	1.670	5.000	4.058	0.673	0.125		
	Coplit	1.670	4.330	3.310	0.623	0.116		
	Gemini	2.000	4.670	3.725	0.604	0.112		
Comprehensiveness*	ChatGPT	1.330	4.670	3.483	0.711	0.132	7.898	<0.001
	Claude	1.330	5.000	4.069	0.884	0.164		
	Coplit	1.330	4.000	3.035	0.880	0.163		
	Gemini	1.670	5.000	3.713	0.834	0.155		
Understandability*	ChatGPT	1.330	4.330	2.827	0.733	0.136	7.821	<0.001
	Claude	1.000	4.670	2.368	0.923	0.171		
	Coplit	1.670	4.000	3.173	0.646	0.120		
	Gemini	1.000	4.670	3.230	0.725	0.135		
Reliability*	ChatGPT	2.330	4.330	3.518	0.433	0.080	4.056	0.009
	Claude	1.330	4.330	3.712	0.705	0.131		
	Coplit	1.330	4.000	3.172	0.614	0.114		
	Gemini	2.330	4.670	3.494	0.609	0.113		
Humanistic care	ChatGPT	1.000	2.330	1.172	0.351	0.065	2.632	0.054
	Claude	1.000	2.330	1.138	0.372	0.069		
	Coplit	1.000	2.330	1.365	0.257	0.048		
	Gemini	1.000	2.330	1.195	0.351	0.065		

* indicates that a statistically significant difference exists among the large language models according to the one-way ANOVA (P < 0.05), necessitating the use of post-hoc analysis.

Analysis from various perspectives reveals that the four LLMs exhibit significant differences only in foundational capabilities, with Claude achieving the highest score (3.399), and Coplit the lowest (2.633). In other dimensions, the models showed no statistically significant differences, indicating that LLMs have not yet demonstrated substantial divergence in core medical domains (Table 2). Although Gemini performed relatively well in prognosis dimension (3.399), its advantage was not statistically significant. *Post hoc* analysis results confirmed that Claude outperformed both Coplit and Gemini in foundational capabilities. These findings underscore Claude’s advantage in this dimension and provide important evidence to guide model selection (Supplemental Digital Content Table 5, available at http://links.lww.com/JS9/F178).

**Table 2:** Scores of four LLMs in different aspects

Different aspects	LLM	Minimum	Maximum	Mean	SD	SE	F	*P* value
Basic*	ChatGPT	1.000	4.670	2.911	1.040	0.190	2.750	0.046
	Claude	1.000	5.000	3.399	1.451	0.265		
	Coplit	1.330	4.000	2.633	0.851	0.155		
	Gemini	1.000	4.000	2.790	0.933	0.170		
Diagnosis	ChatGPT	1.000	4.330	2.777	1.084	0.198	0.695	0.557
	Claude	1.000	4.670	2.689	1.389	0.254		
	Coplit	1.000	4.330	2.978	0.999	0.182		
	Gemini	1.000	4.670	3.078	1.187	0.217		
Treatment	ChatGPT	1.000	4.330	2.944	1.080	0.197	0.387	0.762
	Claude	1.000	5.000	2.934	1.338	0.244		
	Coplit	1.000	4.000	2.710	1.036	0.189		
	Gemini	1.000	4.000	2.722	1.058	0.193		
Prognosis	ChatGPT	1.000	4.330	3.089	1.131	0.206	0.755	0.521
	Claude	1.000	5.000	3.122	1.263	0.231		
	Coplit	1.000	4.000	2.967	1.074	0.196		
	Gemini	1.000	4.330	3.399	1.129	0.206		
Case	ChatGPT	1.000	4.330	2.960	1.124	0.225	1.183	0.319
	Claude	1.000	4.670	3.226	1.353	0.271		
	Coplit	1.330	4.000	2.759	0.820	0.164		
	Gemini	1.000	5.000	3.266	1.294	0.193		

In recent years, the rapid development of artificial intelligence has provided new possibilities for health care, with LLMs demonstrating potential in medical question answering, health consultation, and patient education due to their powerful natural language processing abilities. Particularly in resource-limited regions, LLMs enable round-the-clock, low-cost engagement through mobile devices, helping to alleviate critical shortages in health care personnel^[6,8]^. International research has begun to validate the feasibility of LLMs (e.g., ChatGPT-4o) in medical information generation and clinical decision support[9]. This study is the first to quantitatively assess the performance boundaries of LLMs in lung cancer patient education using a multidimensional framework, revealing the structural disconnect between technological capabilities and clinical needs. While LLMs demonstrate robustness in the delivery of foundational medical knowledge, their consistent underperformance in the dimension of humanistic care highlights a key limitation; current algorithms struggle to simulate contextualized empathy in doctor-patient communication[10]. Although LLMs have demonstrated certain potential in providing medical information, none of the models met the clinical empathy standards in the dimension of clinical empathy. This result highlights the significant shortcomings of LLMs in addressing patients’ psychosocial needs, particularly in managing emotional distress, providing personalized support, and establishing patient-doctor trust. In the era of AI, the core value of physicians lies not only in their technical proficiency but also in their irreplaceable role in maintaining humanistic care in medical practice, as well as their multifunctional responsibilities in technical interpretation, ethical mediation, and decision-making oversight. Physicians need not resist the development of AI nor fear being replaced. The judicious use of AI technology can effectively alleviate the burden of procedural tasks, allowing physicians to focus more on creative diagnostic and therapeutic activities. Meanwhile, LLMs can serve as a valuable complement to physicians’ work by providing patients and health care providers with rapid and accurate information. Future research should further explore the application of hybrid human-AI education models in the medical field, designing and testing educational systems that integrate physicians’ expertise with AI intelligence to enhance the effectiveness and efficiency of medical education. This approach will provide scientific evidence and practical guidance for physicians’ role transformation in the AI era, not only optimizing the allocation of medical resources but also fostering collaborative innovation between the medical and educational sectors. Furthermore, the “understandability-accuracy paradox” identified in this study (where Claude demonstrates optimal comprehensiveness but poorest comprehensibility) imposes new requirements for the application of LLMs in the medical field: it necessitates moving beyond the mere transplantation of academic corpora toward the development of novel systems equipped with intelligent cognitive adaptation capabilities. Specifically, the focus should be on constructing a multi-layered knowledge representation system that offers users with varying needs a progressive information acquisition path, ranging from fundamental concepts to professional details. Particularly in low-resource regions and among populations with low health literacy, there is a critical need to develop personalized, context-specific technological solutions to ensure that patients from diverse backgrounds can genuinely understand and benefit from the model’s recommendations. Additionally, the integration of multimodal explanatory tools and hierarchical information delivery mechanisms should be considered. By incorporating non-textual forms such as charts, animations, and interactive visualizations, complex professional terminology can be effectively supplemented or replaced, thereby enhancing the comprehensibility of information while maintaining the accuracy of core medical concepts. This comprehensive technological strategy not only helps bridge the “empathy gap” between health care providers and patients but also provides a viable technical pathway toward achieving truly inclusive intelligent health care services. LLMs notable limitations in real-time validation, ethical risks, clinical verification, and cross-cultural adaptability still warrant serious attention. Future work will involve deploying patient feedback systems to enhance patient-driven model optimization, establishing dynamic evaluation protocols to quantify clinical utility, and developing ethical guidelines for AI deployment through an interdisciplinary collaborative framework involving ethicists, clinicians, and policymakers.

However, it is undeniable that this study has certain limitations, specifically the absence of comparisons between the research findings and the performance of human physicians or existing educational materials. This limitation is primarily attributable to a combination of the study’s focused objectives, challenges in data acquisition, and numerous ethical and privacy concerns. Future research could focus on establishing a unified evaluation framework and actively securing data from human physicians to conduct comprehensive and in-depth comparative analyses. In this study, the analysis of model performance did not fully explore the potential impact of question difficulty as a critical factor, which may limit our comprehensive understanding of the causes of low humanistic care scores. Future research should consider a more detailed classification and analysis of question difficulty to more accurately assess the model’s performance in handling complex queries and further optimize the model to enhance its effectiveness in complex scenarios. Furthermore, future work will incorporate more open-source models (such as LLaMA and GPT-3.5) for systematic comparison.

## Supplementary Material

## Data Availability

Data available within the article or its supplementary materials.
