# Supplementary material for: Transformation of lung cancer patient education paradigm driven by large language models: a multidimensional performance study
Source: Int J Surg. 2025 Sep 22;112(1):1881–5. doi: 10.1097/JS9.0000000000003503 (PMC12825824; doi:10.1097/JS9.0000000000003503)
Supplement: Supplementary file 1 [file js9-112-1881-001.docx]

**Supplementary Table 1.** Questionnaire

| **Category** | **Number** | **Question** | **Sources** |
| --- | --- | --- | --- |
| **Overview** | 1 | What is lung cancer? | Clinical guidelines, Patient forums |
|  | 2 | What are the clinical features of lung cancer? | Clinical guidelines, Patient forums |
|  | 3 | Is lung cancer hereditary? | Clinical guidelines, Patient forums |
|  | 4 | What are the types of pathological diagnoses for lung cancer? | Clinical guidelines, Expert consultation |
|  | 5 | What are the characteristics of different types of lung cancer? | Clinical guidelines, Expert consultation |
|  | 6 | What are the common metastasis sites of lung cancer? | Clinical guidelines, Expert consultation |
| **Diagnosis** | 7 | What are the early symptoms of lung cancer? | Clinical guidelines, Patient forums |
|  | 8 | What are the diagnostic criteria for lung cancer? | Clinical guidelines, Expert consultation |
|  | 9 | What are the risk factors for lung cancer? | Clinical guidelines, Patient forums |
|  | 10 | What are the auxiliary examinations and assessments that should be performed for lung cancer? | Clinical guidelines, Expert consultation |
|  | 11 | What is the role of CT scan in the diagnosis of lung cancer? | Clinical guidelines, Expert consultation |
|  | 12 | What is the role of genetic testing in the diagnosis of lung cancer? | Clinical guidelines, Expert consultation |
| **Treatment** | 13 | What are the treatment methods for lung cancer? | Clinical guidelines, Expert consultation |
|  | 14 | Is further treatment needed after surgery? | Clinical guidelines, Expert consultation |
|  | 15 | In what situations is targeted therapy for lung cancer applicable? | Clinical guidelines, Expert consultation |
|  | 16 | How effective is chemotherapy for lung cancer? | Clinical guidelines, Expert consultation |
|  | 17 | In what situations is radiotherapy for lung cancer applicable? | Clinical guidelines, Expert consultation |
|  | 18 | What is immunotherapy? | Clinical guidelines, Expert consultation |
| **Prognosis** | 19 | What is the frequency of follow-up after lung cancer surgery? | Clinical guidelines, Expert consultation |
|  | 20 | What should be done if a new nodule is found during follow-up? | Clinical guidelines, Expert consultation |
|  | 21 | How to determine whether lung cancer recurs during follow-up? | Clinical guidelines, Expert consultation |
|  | 22 | How to manage lifestyle after lung cancer surgery? | Clinical guidelines, Patient forums |
|  | 23 | Whether the focus of follow-up for long-term survivors (e.g., more than 5 years after surgery) needs to be adjusted? | Clinical guidelines, Expert consultation |
|  | 24 | How to manage long-term side effects after treatment, such as lung function damage, radiation pneumonitis, etc. | Clinical guidelines, Expert consultation |
| **Case** | 25 | A 68-year-old male patient, with a 40-year history of smoking, has been experiencing cough and blood-tinged sputum for 3 months. Chest X-ray showed an irregular mass in the right upper lung with a rough edge; CT scan suggested that the mass invaded the pleura and mediastinal lymph nodes were enlarged. Lung cancer is initially considered, and bronchoscopy and biopsy are recommended to confirm the pathology. What further tests does the patient need to confirm the diagnosis? | Clinical case records, Expert consultation |
|  | 26 | A 55-year-old female patient, diagnosed with advanced non-small cell lung cancer (NSCLC), with EGFR gene mutation positive. She has received gefitinib targeted therapy, but recently developed drug resistance and tumor progression. How to adjust the next treatment plan? | Clinical case records, Expert consultation |
|  | 27 | A 70-year-old male patient, on the 3rd day after lung cancer surgery, developed pleural effusion and poor drainage. Body temperature was 37.8°C, heart rate was 90 beats/min, respiratory rate was 22 breaths/min, and the drainage tube was blocked. The patient complained of chest tightness. How to deal with the blocked drainage tube? | Clinical case records, Expert consultation |
|  | 28 | A 48-year-old female patient, newly diagnosed with advanced lung cancer, is feeling low and refusing treatment. The patient has obvious anxiety and depression, worrying about the side effects of treatment and family burden. How to help the patient rebuild treatment confidence? | Clinical case records, Expert consultation |
|  | 29 | A 65-year-old male patient, diagnosed with locally advanced non-small cell lung cancer, is not suitable for surgical resection. He has completed concurrent chemoradiotherapy, but developed radiation pneumonitis and shortness of breath. How to relieve the symptoms of radiation pneumonitis? | Clinical case records, Expert consultation |

**Supplementary Table 2a:** Claude 3.7 Responses to Tuberculosis Questions

| **Question** | **Claude 3.7 Response** |
| --- | --- |
| **Topic: Overview** | |
| 1. **What is lung cancer?** | Lung cancer is a disease characterized by the uncontrolled growth of abnormal cells in one or both lungs. These abnormal cells divide rapidly and form tumors that interfere with the lung's ability to provide oxygen to the body through the blood.  **There are two main types of lung cancer:**   1. Non-small cell lung cancer (NSCLC), which is more common (about 80-85% of cases) 2. Small cell lung cancer (SCLC), which is less common but tends to grow faster and spread earlier   **The most common cause of lung cancer is long-term exposure to tobacco smoke, which accounts for about 80-90% of lung cancer cases. Other risk factors include exposure to:**   1. Secondhand smoke 2. Radon gas 3. Asbestos and other carcinogens 4. Air pollution 5. Family history of lung cancer   **Symptoms of lung cancer may include:**   1. Persistent cough 2. Coughing up blood 3. Chest pain 4. Shortness of breath 5. Hoarseness 6. Weight loss 7. Fatigue   Early detection improves treatment outcomes, but unfortunately, lung cancer often doesn't cause symptoms until it has advanced. Treatment options typically include surgery, chemotherapy, radiation therapy, targeted therapy, and immunotherapy, depending on the type and stage of cancer. |
| 1. **What are the clinical features of lung cancer?** | The clinical features of lung cancer can vary depending on the type, location, and stage of the cancer. Here are the key clinical manifestations:   1. **Early Symptoms**   Many patients are asymptomatic in early stages, which contributes to late diagnosis. When symptoms do appear, they may include:   - 1. Persistent cough (most common symptom)   2. Hemoptysis (coughing up blood)   3. Dyspnea (shortness of breath)   4. Chest pain that worsens with deep breathing or coughing   5. Hoarseness or voice changes   6. Recurrent respiratory infections (pneumonia, bronchitis)  1. **Local Disease Features**   Symptoms related to the primary tumor:   - 1. Wheezing or stridor due to airway obstruction   2. Atelectasis (lung collapse)   3. Pleural effusion (fluid around the lungs)   4. Superior vena cava syndrome (in tumors of the upper right lung)   5. Horner's syndrome (ptosis, miosis, anhidrosis) from involvement of sympathetic nerves   6. Pancoast syndrome (shoulder pain radiating down the arm) in apical tumors  1. **Metastatic Disease Features**   As the disease advances, symptoms related to spread may include:   - 1. Bone pain (from bone metastases)   2. Neurological symptoms (headache, seizures, cognitive changes from brain metastases)   3. Hepatomegaly or right upper quadrant pain (liver metastases)   4. Lymphadenopathy, particularly supraclavicular or cervical   5. Jaundice from liver involvement  1. **Paraneoplastic Syndromes**   Lung cancer can cause various paraneoplastic syndromes:   - 1. Hypercalcemia (often in squamous cell carcinoma)   2. Syndrome of inappropriate antidiuretic hormone secretion (SIADH)   3. Cushing's syndrome from ectopic ACTH production   4. Lambert-Eaton myasthenic syndrome   5. Dermatomyositis   6. Hypertrophic pulmonary osteoarthropathy   7. Trousseau's syndrome (migratory thrombophlebitis)  1. **Systemic Symptoms**   Constitutional symptoms that may indicate advanced disease:   - 1. Unexplained weight loss   2. Fatigue   3. Anorexia (loss of appetite)   4. Cachexia (wasting syndrome)   5. Fever of unknown origin   These clinical features can vary significantly between patients, and many may be attributed to other conditions, which can delay diagnosis. The presence of risk factors (smoking history, occupational exposures, family history) alongside these symptoms should raise suspicion for lung cancer. |
| 1. **Is lung cancer hereditary?** | Lung cancer can have a hereditary component, but it's not as strongly hereditary as some other cancers. The relationship between genetics and lung cancer is complex:   1. **Genetic Factors**    1. Only about 8% of lung cancers are thought to have a hereditary component.    2. Having a first-degree relative (parent, sibling, child) with lung cancer increases your risk by about 1.5-2 times, even after accounting for shared smoking habits.    3. The risk increases if multiple family members have had lung cancer or if family members developed it at a young age. 2. **Specific Genetic Mutations**   Several genetic variations have been identified that may increase susceptibility:   - 1. Mutations in genes involved in DNA repair (CHEK2, BRCA1/2)   2. Variations in genes affecting carcinogen metabolism (CYP1A1, GSTM1)   3. Chromosome 6q genetic locus   4. Changes in chromosome 15q25, which contains genes for nicotinic acetylcholine receptors  1. **Hereditary Cancer Syndromes**   Some rare inherited syndromes can increase lung cancer risk:   - 1. Li-Fraumeni syndrome (TP53 mutations)   2. Retinoblastoma (RB1 mutations)  1. **Environmental Interaction**   The most important thing to understand is that genetic factors typically interact with environmental exposures:   - 1. A genetic predisposition may make someone more susceptible to the carcinogenic effects of tobacco smoke or other environmental toxins.   2. Even with a family history, avoiding tobacco smoke dramatically reduces risk.  1. **Perspective**   While genetic factors play a role, environmental factors—particularly smoking—remain by far the most significant risk factor for lung cancer. About 80-90% of lung cancers are linked to smoking, whereas the genetic contribution is much smaller.  If you have a strong family history of lung cancer, it may be worth discussing screening options with a healthcare provider, especially if you have other risk factors. |
| 1. **What are the types of pathological diagnoses for lung cancer?** | The pathological diagnosis of lung cancer involves several classification systems that help guide treatment and determine prognosis. Here are the main types of pathological diagnoses for lung cancer:  **Main Histological Types**   1. **Non-Small Cell Lung Cancer (NSCLC) - approximately 80-85% of cases**    1. Adenocarcinoma - most common type; often arises in peripheral lung tissues    2. Squamous Cell Carcinoma - typically occurs in central airways, associated with smoking    3. Large Cell Carcinoma - poorly differentiated, can occur in any part of the lung    4. Adenosquamous Carcinoma - combines features of adenocarcinoma and squamous cell carcinoma    5. Sarcomatoid Carcinoma - rare, aggressive variant with sarcoma-like features 2. **Small Cell Lung Cancer (SCLC) - approximately 15% of cases**    1. Pure Small Cell Carcinoma    2. Combined Small Cell Carcinoma (with non-small cell component) 3. **Neuroendocrine Tumors**    1. Typical Carcinoid - low-grade, less aggressive    2. Atypical Carcinoid - intermediate-grade    3. Large Cell Neuroendocrine Carcinoma - high-grade    4. Small Cell Carcinoma (also classified here as a high-grade neuroendocrine tumor) 4. **Other Rare Primary Lung Tumors**    1. Salivary gland-type tumors (mucoepidermoid carcinoma, adenoid cystic carcinoma)    2. Sarcomas    3. Lymphomas    4. Hamartomas   **Molecular/Genetic Diagnoses**  Modern pathological diagnosis also includes molecular testing for targetable mutations:   1. EGFR mutations - common in adenocarcinoma, more frequent in non-smokers 2. ALK rearrangements - more common in younger patients and non-smokers 3. ROS1 rearrangements - similar to ALK, targetable with specific therapies 4. BRAF mutations - less common, associated with smoking 5. MET amplification or exon 14 skipping mutations 6. RET fusions 7. NTRK fusions 8. KRAS mutations - common but historically difficult to target 9. HER2 mutations or amplifications   **PD-L1 Status**  PD-L1 expression testing helps determine potential response to immunotherapy:   1. Negative: <1% expression 2. Low: 1-49% expression 3. High: ≥50% expression   **Grade**  Histological grading based on degree of differentiation:   1. Well-differentiated (Grade 1) 2. Moderately differentiated (Grade 2) 3. Poorly differentiated (Grade 3) 4. Undifferentiated (Grade 4)   **Staging-Related Pathology**  Pathological staging (pTNM) includes assessment of:   1. Primary tumor size and invasion (pT) 2. Regional lymph node involvement (pN) 3. Presence of distant metastases (pM)   **Residual Tumor Classification**  After surgical resection:   1. R0: No residual tumor 2. R1: Microscopic residual tumor 3. R2: Macroscopic residual tumor   These pathological diagnoses are crucial for guiding treatment decisions, predicting outcomes, and determining eligibility for targeted therapies and clinical trials. |
| 1. **What are the characteristics of different types of lung cancer?** | **Characteristics of Different Types of Lung Cancer**  **Non-Small Cell Lung Cancers (NSCLC)**  **Adenocarcinoma**   1. Prevalence: Most common type (40% of all lung cancers) 2. Location: Usually peripheral (outer parts of the lung) 3. Cell origin: Glandular cells that produce mucus 4. Growth pattern: Tends to grow more slowly than other types 5. Demographics: Most common type in non-smokers; more common in women 6. Molecular features: Higher frequency of EGFR, ALK, ROS1, BRAF mutations 7. Histology: Glandular structures, mucin production, lepidic, acinar, papillary patterns 8. Prognosis: Generally better than other types when caught early   **Squamous Cell Carcinoma**   1. Prevalence: 25-30% of all lung cancers 2. Location: Central, often involving major bronchi 3. Cell origin: Squamous epithelial cells lining the airways 4. Growth pattern: Often forms cavities 5. Demographics: Strong association with smoking 6. Molecular features: Often has p53 mutations, FGFR1 amplifications, PI3K mutations 7. Histology: Keratinization, intercellular bridges, sheet-like growth 8. Paraneoplastic features: Commonly causes hypercalcemia   **Large Cell Carcinoma**   1. Prevalence: About 10% of lung cancers 2. Location: Can occur anywhere in the lung 3. Cell origin: Poorly differentiated cells without clear lineage 4. Growth pattern: Typically aggressive, rapid growth 5. Histology: Large cells with abundant cytoplasm, prominent nucleoli, no specific pattern 6. Variants: Large cell neuroendocrine carcinoma has particularly poor prognosis   **Small Cell Lung Cancer (SCLC)**   1. Prevalence: 10-15% of all lung cancers 2. Location: Typically central, often with bulky hilar masses 3. Cell origin: Neuroendocrine cells 4. Growth pattern: Extremely aggressive, rapid doubling time (30-50 days) 5. Demographics: Almost exclusively in heavy smokers 6. Molecular features: Nearly universal RB1 and TP53 inactivation 7. Histology: Small cells with scant cytoplasm, nuclear molding, "salt and pepper" chromatin 8. Metastatic behavior: Early and widespread metastasis 9. Paraneoplastic syndromes: SIADH, Cushing's, Lambert-Eaton 10. Staging: Simplified staging (limited vs. extensive) often used 11. Treatment response: Initially very chemosensitive but high recurrence rate   **Neuroendocrine Tumors**  **Carcinoid Tumors**   1. Prevalence: About 1-2% of lung tumors 2. Types: Typical (low-grade) and atypical (intermediate-grade) 3. Location: Usually central (75%) 4. Growth pattern: Slow-growing, less aggressive 5. Histology: Organoid nesting pattern, uniform cells with finely granular chromatin 6. Molecular features: Often lack common mutations seen in other lung cancers 7. Symptoms: May cause carcinoid syndrome (flushing, diarrhea) but rarely in lung primaries 8. Prognosis: Typical carcinoids have excellent prognosis (5-year survival >90%)   **Large Cell Neuroendocrine Carcinoma**   1. Prevalence: Rare (about 3% of lung cancers) 2. Behavior: High-grade neuroendocrine tumor with poor prognosis 3. Histology: Large cells with neuroendocrine morphology and markers 4. Treatment approach: Often treated similarly to SCLC   **Other Distinctive Features**  **Imaging Characteristics**   1. Adenocarcinoma: Often appears as ground-glass opacity or part-solid nodule 2. Squamous cell: Frequently shows cavitation 3. SCLC: Typically presents with bulky hilar mass and extensive lymphadenopathy   **Treatment Responsiveness**   1. NSCLC: Surgery for early stages; targeted therapy options for specific mutations 2. SCLC: Primarily treated with chemotherapy and radiation; rarely surgical candidates 3. Carcinoids: Primarily surgical treatment; generally resistant to conventional chemotherapy   **Metastatic Patterns**   1. Adenocarcinoma: Tendency for early distant metastasis (brain, bone, liver) 2. Squamous cell: Often locally advanced before metastasizing 3. SCLC: Early and widespread metastases, including brain, liver, bone, adrenal   These characteristics guide diagnostic approaches, treatment selection, and prognostic expectations for patients with different types of lung cancer. |
| 1. **What are the common metastasis sites of lung cancer?** | Lung cancer commonly metastasizes to several sites throughout the body, with patterns that can vary by lung cancer type. Here are the most common metastatic sites:  **Most Frequent Metastatic Sites**   1. **Brain**    1. Particularly common with small cell lung cancer and adenocarcinoma    2. Often presents with headaches, seizures, cognitive changes, or focal neurological deficits    3. Represents approximately 10-30% of all lung cancer patients at some point in their disease 2. **Bone**    1. Affects 30-40% of patients with advanced lung cancer    2. Common locations include spine, ribs, pelvis, proximal long bones, and skull    3. Often presents with pain, pathological fractures, spinal cord compression, or hypercalcemia 3. **Liver**    1. Present in about 20-30% of patients with metastatic disease    2. More common in small cell lung cancer    3. May cause right upper quadrant pain, hepatomegaly, jaundice, or abnormal liver function tests 4. **Adrenal Glands**    1. Occurs in approximately 20-40% of patients with metastatic disease    2. Often asymptomatic and discovered incidentally on imaging    3. May occasionally cause adrenal insufficiency 5. **Lymph Nodes**    1. Regional: Hilar, mediastinal (N1, N2, N3 disease)    2. Distant: Supraclavicular, cervical, axillary (considered M1 disease)    3. Can cause lymphedema, pain, or compression of adjacent structures   **Less Common but Significant Sites**   1. **Pleura**    1. Malignant pleural effusions or pleural nodules    2. Can cause dyspnea, chest pain, and respiratory compromise    3. Common with adenocarcinoma 2. **Contralateral Lung**   Separate tumor nodules in the opposite lung are considered metastatic disease   1. **Pericardium**    1. Can lead to pericardial effusion or tamponade    2. May cause dyspnea, chest pain, or hypotension 2. **Kidneys and Soft Tissue**   Less common but can occur in advanced disease   1. **Gastrointestinal Tract**   Uncommon location but may occur with small cell lung cancer  **Metastatic Patterns by Histological Type**   1. Adenocarcinoma: Higher propensity for brain, bone, and liver metastases 2. Small Cell Lung Cancer: Widespread early metastases, particularly to brain, liver, bone, and adrenal glands 3. Squamous Cell Carcinoma: Tends to remain localized longer before metastasizing; when it does, bone and liver are common sites   This pattern of metastasis influences surveillance strategies, staging workup, and prophylactic treatments (such as prophylactic cranial irradiation for small cell lung cancer). |
| **Topic: Diagnosis** | |
| 1. **What are the early symptoms of lung cancer?** | Early symptoms of lung cancer can be subtle and are often mistaken for other conditions, which can delay diagnosis. Many people with early-stage lung cancer have no symptoms at all, which is why it's frequently diagnosed at more advanced stages. Here are the most common early signs and symptoms:   1. **Respiratory Symptoms**    1. Persistent cough: A cough that doesn't go away or worsens over time    2. Coughing up blood (hemoptysis): Even small amounts should be evaluated    3. Shortness of breath (dyspnea): Initially with exertion, later at rest    4. Wheezing: New-onset wheezing, especially in smokers    5. Recurrent respiratory infections: Repeated bouts of bronchitis or pneumonia in the same area of the lung 2. **Chest Symptoms**    1. Chest pain: Often dull, aching, and persistent, may worsen with deep breathing or coughing    2. Hoarseness: Persistent voice changes due to tumor affecting the recurrent laryngeal nerve 3. **Systemic Symptoms**    1. Unexplained weight loss: Losing weight without trying    2. Fatigue: Persistent tiredness that doesn't improve with rest    3. Low-grade fever: Unexplained mild fever, especially if persistent 4. **Less Common Early Signs**    1. Shoulder/arm pain: Particularly in tumors at the lung apex (Pancoast tumors)    2. Facial or neck swelling: Due to superior vena cava obstruction    3. Clubbing of fingers: Thickening of fingertips due to decreased oxygen   It's important to note that these symptoms are often attributed to other causes, especially in smokers who may have chronic respiratory issues like bronchitis. Any persistent symptom, particularly in individuals with risk factors for lung cancer (smoking history, occupational exposures, family history), warrants medical evaluation. |
| 1. **What are the diagnostic criteria for lung cancer?** | Diagnosing lung cancer involves multiple steps, with pathological confirmation being the definitive standard. Here's a comprehensive overview of the diagnostic criteria for lung cancer:  **Initial Evaluation**   1. **Clinical Assessment**    1. Comprehensive history including risk factors (smoking, occupational exposures)    2. Physical examination findings (abnormal breath sounds, lymphadenopathy)    3. Review of symptoms (cough, hemoptysis, weight loss, etc.) 2. **Imaging Studies**    1. Chest X-ray: Often the first test revealing abnormalities    2. CT scan: Higher sensitivity for detecting lung nodules and masses   Features suggestive of malignancy: Spiculated margins, size >8mm, growth over time, upper lobe location   - 1. PET/CT: Helps distinguish benign from malignant lesions based on metabolic activity   SUV >2.5 generally concerning for malignancy   - 1. Brain MRI: Often performed for staging once diagnosis is confirmed   **Pathological Confirmation**   1. **Tissue Sampling Methods**    1. Bronchoscopy: For central lesions       1. Endobronchial biopsy, brushings, washings       2. Endobronchial ultrasound-guided transbronchial needle aspiration (EBUS-TBNA) for hilar/mediastinal nodes    2. CT-guided percutaneous needle biopsy: For peripheral lesions    3. Surgical procedures:       1. Mediastinoscopy for mediastinal lymph nodes       2. Video-assisted thoracoscopic surgery (VATS) biopsy       3. Thoracotomy (open lung biopsy)    4. Liquid biopsy: Circulating tumor DNA detection (emerging approach) 2. **Cytology/Histology Criteria**    1. Cytological diagnosis: Sputum cytology, pleural fluid analysis    2. Histological diagnosis: Definitive method       1. Confirmation of malignant cells       2. Classification into specific histological types   **Molecular and Immunohistochemical Testing**   1. **Immunohistochemical (IHC) Markers**    1. TTF-1: Typically positive in adenocarcinoma, SCLC    2. p40/p63: Typically positive in squamous cell carcinoma    3. Chromogranin/Synaptophysin: Positive in neuroendocrine tumors    4. CK7/CK20 profile: Helps distinguish primary vs. metastatic 2. **Molecular Testing (for NSCLC, particularly adenocarcinoma)**    1. EGFR mutation    2. ALK rearrangement    3. ROS1 rearrangement    4. BRAF mutation    5. NTRK fusion    6. MET exon 14 skipping    7. RET fusion    8. KRAS mutation    9. HER2 mutation 3. **PD-L1 Testing**    1. IHC evaluation of tumor proportion score (TPS)    2. Guides immunotherapy decisions   **Staging Assessment**   1. **TNM Staging Criteria (8th edition AJCC/UICC)**    1. T: Tumor size and local invasion    2. N: Regional lymph node involvement    3. M: Distant metastasis    4. Combined to determine stage grouping (I-IV) 2. **Performance Status**    1. ECOG or Karnofsky score    2. Critical for treatment decision-making   **Additional Considerations**   1. **Pulmonary Function Tests**   Assesses respiratory reserve for treatment planning   1. **Multidisciplinary Team Review**   Confirmation of diagnosis and treatment plan by tumor board  The definitive diagnosis of lung cancer requires pathological confirmation of malignant cells through one of the tissue sampling methods. The specific diagnostic approach often depends on the location of the suspicious lesion, patient factors, and local expertise. |
| 1. **What are the risk factors for lung cancer?** | **Risk Factors for Lung Cancer**   1. **Tobacco Use**    1. Cigarette smoking: The leading cause, responsible for 80-90% of lung cancer deaths       1. Risk increases with duration and intensity (pack-years)       2. 20-25 times higher risk in long-term smokers compared to never-smokers    2. Cigar and pipe smoking: Lower risk than cigarettes but still significant    3. Secondhand smoke exposure: 20-30% increased risk in non-smokers 2. **Environmental and Occupational Exposures**    1. Radon gas: Second leading cause; naturally occurring radioactive gas in soil    2. Asbestos: Particularly when combined with smoking (synergistic effect)    3. Other workplace carcinogens:       1. Arsenic       2. Chromium       3. Nickel       4. Beryllium       5. Cadmium       6. Silica       7. Diesel exhaust       8. Coal smoke    4. Air pollution: Both outdoor pollution and indoor cooking fumes    5. Radiation exposure: Previous radiation therapy to chest area 3. **Genetic and Personal Factors**    1. Family history: Increased risk with first-degree relatives who had lung cancer    2. Personal history of:       1. Previous lung cancer       2. COPD or pulmonary fibrosis       3. Tuberculosis    3. Genetic predisposition:       1. TP53, EGFR, KRAS mutations       2. Polymorphisms in genes involved in carcinogen metabolism 4. **Other Medical Conditions**    1. Chronic Obstructive Pulmonary Disease (COPD): Independent risk factor beyond smoking    2. Interstitial lung disease/Pulmonary fibrosis: 3-5 times increased risk    3. HIV infection: Independent risk factor, regardless of smoking status 5. **Demographic Factors**    1. Age: Risk increases with age; majority of cases diagnosed after age 65    2. Sex: Historically higher in men, but increasing in women    3. Race/ethnicity: Variations in risk and tumor biology exist across different populations 6. **Lifestyle Factors**    1. Diet: Low consumption of fruits and vegetables associated with increased risk    2. Physical inactivity: May contribute to risk    3. Alcohol: Heavy consumption may increase risk, particularly in combination with smoking   The risk from multiple factors is often multiplicative rather than additive. For example, a person who smokes and has occupational asbestos exposure has a risk much higher than the sum of each individual risk factor. Smoking cessation significantly reduces risk over time, though former smokers maintain some elevated risk compared to never-smokers. |
| 1. **What are the auxiliary examinations and assessments that should be performed for lung cancer?** | **Auxiliary Examinations and Assessments for Lung Cancer**  **Imaging Studies**   1. **Initial Diagnosis**    1. Chest X-ray: Often the first test revealing abnormalities    2. CT scan with contrast: Essential for detailed evaluation of the primary tumor and regional lymph nodes    3. PET/CT scan: Evaluates metabolic activity and helps detect metastatic disease    4. Brain MRI: Recommended for all NSCLC patients with stage II-IV and all SCLC patients 2. **Additional Imaging for Metastatic Evaluation**    1. Bone scan: If bone metastases are suspected and not evaluated by PET/CT    2. Abdominal CT/MRI: If liver or adrenal metastases are suspected    3. Whole-body MRI: Sometimes used as an alternative to PET/CT   **Tissue Sampling Procedures**   1. Bronchoscopy: For central tumors    1. Endobronchial biopsy    2. Bronchoalveolar lavage    3. Bronchial brushings/washings 2. EBUS-TBNA (Endobronchial ultrasound-guided transbronchial needle aspiration): For mediastinal and hilar lymph nodes 3. CT-guided needle biopsy: For peripheral lesions 4. Thoracentesis: If pleural effusion is present 5. Mediastinoscopy: Surgical sampling of mediastinal lymph nodes 6. VATS (Video-assisted thoracoscopic surgery): For diagnostic and therapeutic purposes 7. Thoracotomy: When other methods are inconclusive   **Laboratory Tests**   1. Complete blood count: Baseline assessment and monitoring 2. Comprehensive metabolic panel: Evaluate liver, kidney function 3. Coagulation studies: Prior to invasive procedures 4. Tumor markers: CEA, CYFRA 21-1, NSE (limited utility) 5. Arterial blood gases: If respiratory insufficiency is suspected   **Pathological and Molecular Testing**   1. Histological classification: Differentiate between NSCLC and SCLC 2. Immunohistochemistry: Further characterize tumor type 3. Molecular testing for targeted therapy selection:    1. EGFR mutation    2. ALK and ROS1 rearrangements    3. BRAF mutation    4. NTRK fusions    5. MET exon 14 skipping    6. RET fusions    7. KRAS G12C mutation    8. HER2 mutations 4. PD-L1 expression: To determine eligibility for immunotherapy 5. Tumor mutational burden (TMB): May predict immunotherapy response   **Pulmonary Function Assessment**   1. Pulmonary function tests (PFTs): Spirometry, lung volumes, diffusion capacity 2. Cardiopulmonary exercise testing: For borderline surgical candidates 3. Split lung function studies: For patients with borderline lung function 4. Quantitative perfusion scan: To predict post-surgical lung function   **Cardiac Assessment**   1. ECG: Baseline cardiac evaluation 2. Echocardiogram: If cardiac involvement suspected or prior to certain chemotherapies 3. Stress testing: For patients with cardiac risk factors being considered for surgery   **Invasive Staging Procedures**   1. Mediastinal lymph node evaluation:    1. Mediastinoscopy    2. EBUS/EUS    3. VATS 2. Surgical staging: When non-invasive methods are inconclusive   **Performance Status Assessment**   1. ECOG (Eastern Cooperative Oncology Group) Scale: 0-5 2. Karnofsky Performance Status: 0-100% 3. Comprehensive geriatric assessment: For elderly patients   **Nutritional and Functional Assessment**   1. Nutritional status evaluation: Body mass index, weight loss 2. Functional capacity assessment: 6-minute walk test 3. Quality of life questionnaires: EORTC QLQ-C30, FACT-L   **Risk Assessment Tools**   1. Thoracic revised cardiac risk index: For perioperative risk 2. Multidisciplinary risk assessment: Involving pulmonologists, thoracic surgeons, oncologists   These comprehensive examinations help in accurate diagnosis, staging, treatment planning, and monitoring of lung cancer patients throughout their care journey. |
| 1. **What is the role of CT scan in the diagnosis of lung cancer?** | CT scanning plays a central role in the diagnosis, staging, and management of lung cancer. Here are the key functions of CT scanning in lung cancer:  **Initial Detection and Characterization**   1. Superior sensitivity: Detects nodules as small as 1-2mm (versus 8-10mm on chest X-ray) 2. Nodule characterization: Evaluates features suggestive of malignancy:    1. Spiculated or irregular margins    2. Solid versus part-solid or ground-glass appearance    3. Size (>8mm more concerning)    4. Location (upper lobes more common for primary lung cancer)    5. Presence of calcification patterns    6. Enhancement patterns after contrast 3. Serial imaging: Allows assessment of growth rate (volume doubling time)   **Staging Assessment**   1. T staging: Evaluates primary tumor size, location, and local invasion    1. Chest wall involvement    2. Mediastinal invasion    3. Pleural involvement    4. Satellite nodules 2. N staging: Assesses hilar and mediastinal lymph nodes    1. Size (>1cm short axis diameter is suspicious)    2. Morphology and enhancement patterns 3. M staging: Detects metastases to:    1. Contralateral lung    2. Pleura    3. Liver and adrenal glands (on abdominal CT)    4. Allows assessment for bone metastases   **Procedural Guidance**   1. Guides percutaneous needle biopsies of peripheral lesions 2. Helps plan endobronchial approaches by showing relationship to airways 3. Supports surgical planning by delineating anatomical relationships   **Treatment Planning**   1. Radiation therapy planning: Defines target volumes and critical structures 2. Surgical assessment: Determines resectability and appropriate surgical approach 3. Baseline for response assessment: Provides measurements for evaluating treatment response   **Surveillance and Follow-up**   1. Post-treatment monitoring: Detects recurrence during follow-up 2. Lung cancer screening: Low-dose CT is now standard for high-risk individuals 3. Evaluation of treatment complications: Radiation pneumonitis, drug toxicities   **Special CT Techniques**   1. High-resolution CT (HRCT): Better characterizes ground-glass opacities 2. CT perfusion: Can help distinguish benign from malignant lesions 3. Dual-energy CT: Improves lesion characterization 4. Four-dimensional CT (4D-CT): Accounts for respiratory motion in radiation planning   While CT is essential, it has limitations including radiation exposure, limited specificity (many benign conditions can mimic malignancy), and limited functional assessment compared to PET. CT findings typically require pathological confirmation before definitive diagnosis of lung cancer can be made. |
| 1. **What is the role of genetic testing in the diagnosis of lung cancer?** | Genetic testing has become a crucial component in diagnosing and managing lung cancer, particularly non-small cell lung cancer (NSCLC). Here's the comprehensive role genetic testing plays:  **Primary Roles of Genetic Testing in Lung Cancer**   1. **Guiding Treatment Selection**    1. Identifies patients eligible for targeted therapies    2. Determines potential response to immunotherapy    3. Informs chemotherapy selection in some cases 2. **Prognostic Assessment**    1. Provides information about expected disease course    2. Helps predict aggressiveness and recurrence risk 3. **Aids in Difficult Diagnoses**    1. Distinguishes primary from metastatic lung lesions    2. Supports classification of poorly differentiated tumors   **Key Molecular Alterations Tested**   1. **Driver Mutations with FDA-Approved Targeted Therapies**    1. EGFR mutations (exon 19 deletions, L858R, T790M, exon 20 insertions)       1. Frequency: ~15% of Western, ~40-50% of Asian adenocarcinoma patients       2. Treatments: Osimertinib, erlotinib, gefitinib, afatinib, dacomitinib    2. ALK rearrangements       1. Frequency: 3-7% of NSCLC       2. Treatments: Alectinib, brigatinib, lorlatinib, crizotinib    3. ROS1 rearrangements       1. Frequency: 1-2% of NSCLC       2. Treatments: Entrectinib, crizotinib    4. BRAF V600E mutation       1. Frequency: 1-3% of NSCLC       2. Treatments: Dabrafenib + trametinib    5. NTRK fusions       1. Frequency: <1% of NSCLC       2. Treatments: Larotrectinib, entrectinib    6. MET exon 14 skipping       1. Frequency: 3-4% of NSCLC       2. Treatments: Capmatinib, tepotinib    7. RET fusions       1. Frequency: 1-2% of NSCLC       2. Treatments: Selpercatinib, pralsetinib    8. KRAS G12C mutation       1. Frequency: ~13% of NSCLC       2. Treatment: Sotorasib, adagrasib 2. **Predictive Biomarkers for Immunotherapy**    1. PD-L1 expression level       1. Measured by immunohistochemistry (IHC)       2. Higher levels generally correlate with better immunotherapy response    2. Tumor Mutational Burden (TMB)   High TMB may predict better response to immunotherapy regardless of PD-L1 status   - 1. Microsatellite Instability (MSI) and Mismatch Repair Deficiency (dMMR)   Rare in lung cancer but predictive of immunotherapy response   1. **Emerging Biomarkers**    1. HER2 mutations and amplifications    2. NRG1 fusions    3. SMARCA4/BRG1 mutations    4. KEAP1/NRF2 pathway alterations   **Testing Methodologies**   1. **Single-gene tests**    1. PCR-based methods    2. Fluorescence in situ hybridization (FISH) for rearrangements    3. Immunohistochemistry for ALK, ROS1, and PD-L1 2. **Next-Generation Sequencing (NGS) panels**    1. Comprehensive genomic profiling of multiple genes simultaneously    2. Can detect point mutations, insertions/deletions, copy number variations, and gene fusions    3. Increasingly the standard of care 3. **Liquid Biopsy (Circulating Tumor DNA)**    1. Non-invasive alternative when tissue is insufficient or inaccessible    2. Used for initial diagnosis, monitoring treatment response, and detecting resistance mechanisms    3. Less sensitive than tissue testing but improving   **Implementation in Clinical Practice**   1. Testing Timing: Recommended at diagnosis of advanced NSCLC before first-line treatment 2. Sample Requirements: Adequate tissue sample needed (core biopsy preferred over FNA) 3. Reflex Testing: Many centers automatically test for key alterations upon NSCLC diagnosis 4. Sequential vs. Comprehensive: Trend toward upfront NGS testing rather than sequential single-gene tests 5. Repeat Testing: Often performed at progression to identify resistance mechanisms   Genetic testing has transformed lung cancer from a disease treated with a one-size-fits-all approach to a collection of molecularly defined subtypes with specific treatments, dramatically improving outcomes for patients with targetable alterations. |
| **Topic: Treatment** | |
| **What are the treatment methods for lung cancer?** | **Treatment Methods for Lung Cancer**  Treatment approaches for lung cancer vary based on the cancer type, stage, molecular profile, patient's overall health status, and preferences. Here's a comprehensive overview of treatment methods:  **Surgery**  **Early-Stage Disease (Stages I-II and select Stage III)**   1. Lobectomy: Removal of an entire lobe (preferred approach) 2. Pneumonectomy: Removal of an entire lung (when necessary) 3. Segmentectomy/Wedge resection: Removal of part of a lobe (for small tumors or compromised lung function) 4. Video-Assisted Thoracoscopic Surgery (VATS): Minimally invasive approach 5. Robotic-assisted surgery: Enhanced precision for complex cases 6. Lymph node dissection: Performed alongside resection for staging   **Radiation Therapy**   1. External Beam Radiation Therapy (EBRT)    1. Conventional fractionation    2. Hypofractionation (fewer, larger doses) 2. Stereotactic Body Radiation Therapy (SBRT): Precise, high-dose treatment for early-stage disease 3. Intensity-Modulated Radiation Therapy (IMRT): Shapes radiation dose to tumor 4. Proton Therapy: Reduces radiation to surrounding tissues 5. Brachytherapy: Internal radiation sources (less common) 6. Palliative radiation: For symptom control in advanced disease   **Systemic Therapies**  **Chemotherapy**   1. Platinum-based combinations: Standard for many patients   Cisplatin/carboplatin with pemetrexed, gemcitabine, paclitaxel, or docetaxel   1. Adjuvant chemotherapy: After surgery for stages II-IIIA 2. Neoadjuvant chemotherapy: Before surgery in select cases 3. Concurrent chemoradiation: For locally advanced disease   **Targeted Therapies**   1. EGFR inhibitors: Osimertinib, erlotinib, gefitinib, afatinib 2. ALK inhibitors: Alectinib, brigatinib, lorlatinib, crizotinib 3. ROS1 inhibitors: Entrectinib, crizotinib 4. BRAF/MEK inhibitors: Dabrafenib + trametinib 5. MET inhibitors: Capmatinib, tepotinib 6. RET inhibitors: Selpercatinib, pralsetinib 7. NTRK inhibitors: Larotrectinib, entrectinib 8. KRAS G12C inhibitors: Sotorasib, adagrasib 9. Anti-angiogenic agents: Bevacizumab, ramucirumab   **Immunotherapy**   1. PD-1/PD-L1 inhibitors:   Pembrolizumab, nivolumab, atezolizumab, cemiplimab, durvalumab   1. CTLA-4 inhibitors: Ipilimumab (often in combination) 2. Combination approaches: Dual immunotherapy or chemoimmunotherapy   **Multimodality Approaches**   1. Concurrent chemoradiation: Standard for unresectable stage III NSCLC 2. Trimodality therapy: Chemotherapy, radiation, and surgery for select stage III 3. Consolidation immunotherapy: Following chemoradiation (durvalumab) 4. Adjuvant targeted therapy: Following surgery for EGFR-positive NSCLC 5. Neoadjuvant immunotherapy: Before surgery in select patients   **Local Therapies for Advanced Disease**   1. Radiofrequency ablation: Thermal destruction of small tumors 2. Cryotherapy: Freezing tumors (bronchoscopic or percutaneous) 3. Photodynamic therapy: Light-activated treatment for endobronchial disease 4. Endobronchial stenting: For airway obstruction 5. Pleurodesis: For malignant pleural effusions   **Special Considerations for Small Cell Lung Cancer (SCLC)**   1. Limited-stage: Concurrent chemoradiation with platinum/etoposide 2. Extensive-stage: Chemotherapy (platinum/etoposide) with immunotherapy 3. Prophylactic cranial irradiation (PCI): To prevent brain metastases 4. Surgery: Rarely, for very early-stage disease   **Supportive and Palliative Care**   1. Pain management: Medications, nerve blocks, radiation 2. Dyspnea management: Oxygen, medications, thoracentesis 3. Nutritional support: Dietary consultation, supplements 4. Pulmonary rehabilitation: Exercise, breathing techniques 5. Psychosocial support: Counseling, support groups 6. Advanced care planning: Goals of care discussions   **Emerging Approaches**   1. Tumor-treating fields (TTFields): Electric field therapy 2. Bispecific antibodies: Engaging immune cells to target tumors 3. Antibody-drug conjugates: Targeted delivery of cytotoxins 4. Cell therapies: CAR-T and other cellular approaches 5. Circulating tumor DNA-guided therapy: Precision treatment based on liquid biopsy   Treatment selection is increasingly personalized based on molecular profiling and biomarkers, with multidisciplinary tumor boards often coordinating complex treatment plans. |
| 1. **Is further treatment needed after surgery?** | Whether further treatment is needed after lung cancer surgery (known as adjuvant therapy) depends on several factors including cancer stage, tumor characteristics, surgical margins, and patient health status. Here's a breakdown:  **Early-Stage Disease (Stage I)**   1. **Stage IA (T1N0M0):**    1. Generally, no adjuvant therapy needed if complete resection with negative margins    2. Observation with regular follow-up is standard    3. Exception: High-risk features may warrant adjuvant chemotherapy consideration 2. **Stage IB (T2aN0M0):**    1. Controversial area; decisions are individualized    2. Observation is reasonable for many patients    3. Adjuvant chemotherapy may be considered for tumors >4cm or with high-risk features (poorly differentiated, vascular invasion, visceral pleural involvement)   **Intermediate-Stage Disease (Stage II)**  **Stage II (N1 disease or T2b-T3N0):**   - 1. Adjuvant chemotherapy is recommended   2. Typically 4 cycles of platinum-based doublet (cisplatin/vinorelbine or carboplatin/paclitaxel)   3. Provides approximately 5-10% absolute improvement in 5-year survival   **Locally-Advanced Disease (Stage III)**  **Stage IIIA:**   - 1. Adjuvant chemotherapy strongly recommended   2. Postoperative radiation therapy (PORT) considered for positive margins or N2 disease   3. Some patients receive neoadjuvant therapy (before surgery) instead   **Molecular-Targeted Adjuvant Therapy**   1. EGFR mutations: Osimertinib approved for adjuvant therapy in stage IB-IIIA EGFR-positive NSCLC 2. ALK-positive: Clinical trials evaluating ALK inhibitors in adjuvant setting 3. Testing for targetable mutations is increasingly recommended even in early-stage disease   **Other Considerations**   1. Surgical margins: Positive margins may require re-resection or radiation therapy 2. Nodal upstaging: Finding unexpected lymph node involvement may change adjuvant plans 3. Performance status: Patient must be healthy enough to tolerate adjuvant therapy 4. Timing: Adjuvant therapy ideally begins within 6-8 weeks after surgery   **Follow-up After Treatment**  Regardless of whether adjuvant therapy is given, follow-up typically includes:   1. Regular CT scans (every 3-6 months initially, then extending intervals) 2. Physical examinations 3. Consideration of pulmonary function tests to monitor lung function   The decision about adjuvant therapy should be made by a multidisciplinary team considering all these factors, and patient preferences should be incorporated into the treatment plan after thorough discussion of potential benefits and risks. |
| 1. **In what situations is targeted therapy for lung cancer applicable?** | Targeted therapy for lung cancer is applicable in specific scenarios based primarily on the molecular profile of the tumor. Here are the key situations where targeted therapy is appropriate:  **Based on Molecular Alterations**   1. **EGFR Mutations**    1. First-line treatment for metastatic NSCLC with sensitizing EGFR mutations (exon 19 deletions, L858R)    2. Osimertinib for adjuvant treatment of stage IB-IIIA NSCLC after complete resection    3. Osimertinib or newer agents for acquired T790M resistance mutation    4. EGFR exon 20 insertion-specific drugs (amivantamab, mobocertinib) 2. **ALK Rearrangements**    1. First-line for metastatic disease with ALK fusion (alectinib preferred)    2. Sequential ALK inhibitors based on resistance patterns (brigatinib, lorlatinib) 3. **ROS1 Rearrangements**   First-line for metastatic disease with ROS1 fusion (entrectinib, crizotinib)   1. **BRAF V600E Mutation**   Combination therapy with BRAF/MEK inhibitors (dabrafenib + trametinib)   1. **NTRK Fusions**   Rare but actionable with larotrectinib or entrectinib   1. **MET Exon 14 Skipping Mutations**   Targeted with capmatinib or tepotinib   1. **RET Fusions**   Targeted with selpercatinib or pralsetinib   1. **KRAS G12C Mutation**   Recently targetable with sotorasib or adagrasib   1. **HER2 Mutations/Amplifications**   Emerging targets with trastuzumab deruxtecan and other agents  **Treatment Settings**   1. **Advanced/Metastatic Disease (Stage IV)**    1. Primary setting for targeted therapy    2. First-line or subsequent lines based on molecular profile    3. Often continued beyond progression if clinical benefit persists 2. **Locally Advanced Disease (Stage III)**    1. Emerging role in combination with other modalities    2. Sequential use after chemoradiation and/or surgery in clinical trials 3. **Early Stage Disease (Stage I-II)**    1. Adjuvant osimertinib for EGFR-positive disease after surgery    2. Other targeted therapies being evaluated in adjuvant setting 4. **Oligometastatic Disease**   Combined with local treatments (surgery, radiation) for limited metastases  **Special Clinical Scenarios**   1. **CNS Metastases**    1. Newer targeted agents with enhanced CNS penetration    2. May defer or avoid whole brain radiation 2. **Acquired Resistance**    1. Switching to next-generation inhibitors    2. Combination approaches targeting resistance mechanisms 3. **Maintenance Therapy**   Continued use after initial response to delay progression   1. **Elderly or Poor Performance Status Patients**   Often better tolerated than chemotherapy for these populations   1. **Never-Smokers or Light Smokers**   Higher likelihood of harboring actionable mutations  Molecular testing is now recommended for all advanced non-squamous NSCLC and for select patients with squamous cell carcinoma (younger age, never-smokers). The detection of these molecular alterations through tissue or liquid biopsy is required before initiating targeted therapy. |
| **How effective is chemotherapy for lung cancer?** | The effectiveness of chemotherapy for lung cancer varies considerably depending on the type and stage of lung cancer, as well as patient-specific factors. Here's an overview of its effectiveness:  **Non-Small Cell Lung Cancer (NSCLC)**  **Early Stage (I-II):**   1. As adjuvant therapy after surgery: Improves 5-year survival by approximately 5-10% in stage II-IIIA 2. Stage IA generally does not benefit from adjuvant chemotherapy 3. Stage IB shows marginal benefit, typically reserved for tumors >4cm   **Locally Advanced (Stage III):**   1. With concurrent radiation: Response rates of 60-70% 2. Improves median survival from 11 months (radiation alone) to 14-17 months 3. 5-year survival rates increase from 5-10% to 15-25%   **Advanced/Metastatic (Stage IV):**   1. Response rates: 20-35% with platinum-based doublets 2. Disease control rate (including stable disease): 60-70% 3. Median survival: 8-12 months (vs. 4-5 months with supportive care alone) 4. year survival: 30-40% 5. 5-year survival: <5%   **Small Cell Lung Cancer (SCLC)**  **Limited Stage:**   1. Response rates with chemoradiation: 80-90% (complete responses in 45-75%) 2. Median survival: 15-20 months 3. 5-year survival: 15-25%   **Extensive Stage:**   1. Response rates: 60-70% with platinum/etoposide 2. Median survival: 9-11 months 3. 5-year survival: 1-2% 4. Addition of immunotherapy has improved median survival to 12-13 months   **Factors Affecting Effectiveness**  **Positive Factors:**   1. Good performance status 2. Adequate organ function 3. Younger age 4. Adenocarcinoma histology (for pemetrexed-based regimens) 5. No significant weight loss   **Negative Factors:**   1. Poor performance status 2. Significant comorbidities 3. Advanced age with frailty 4. Extensive prior treatment   **Chemotherapy in Context**  Modern lung cancer treatment has evolved substantially with the addition of:   1. Targeted therapies for driver mutations (much more effective than chemotherapy when applicable) 2. Immunotherapy (has largely replaced chemotherapy as first-line for many patients) 3. Maintenance approaches (extending benefit of initial therapy)   In summary, while chemotherapy provides modest benefits for many lung cancer patients, its effectiveness is greatest in small cell lung cancer, as adjuvant therapy in early-stage NSCLC, and in combination with radiation for locally advanced disease. In the metastatic setting, chemotherapy's role is increasingly being refined to specific contexts and combinations with other modalities. |
| 1. **In what situations is radiotherapy for lung cancer applicable?** | Radiotherapy plays a versatile role in lung cancer management across various stages and clinical scenarios. Here are the key situations where radiotherapy is applicable:  **Early-Stage Disease (Stage I-II)**   1. **Stereotactic Body Radiation Therapy (SBRT/SABR)**    1. Primary treatment for medically inoperable patients    2. Alternative to surgery in operable patients who decline surgery    3. Achieves local control rates of 85-95% in carefully selected patients    4. Typically delivered in 1-5 fractions with high biological effective doses 2. **Post-operative Radiation Therapy (PORT)**    1. Generally not recommended for completely resected N0-N1 disease    2. May be considered for positive margins when re-resection not feasible   **Locally Advanced Disease (Stage III)**   1. **Concurrent Chemoradiation**    1. Standard of care for unresectable stage III NSCLC    2. Typically 60-66 Gy in 30-33 fractions with platinum-based chemotherapy    3. Often followed by consolidation durvalumab (immunotherapy) 2. **Sequential Chemoradiation**    1. Alternative for patients unable to tolerate concurrent approach    2. Less effective but better tolerated in frail patients 3. **Preoperative (Neoadjuvant) Radiation**    1. Part of trimodality approach for select stage IIIA patients    2. May improve resectability of superior sulcus tumors 4. **Postoperative Radiation**    1. Considered for N2 disease or positive margins after surgery    2. Controversial with mixed evidence on survival benefit   **Advanced/Metastatic Disease (Stage IV)**   1. **Oligometastatic Disease**    1. SBRT to limited metastatic sites in good performance status patients    2. Can achieve long-term disease control in select patients 2. **Palliative Radiation**    1. Symptomatic primary tumor (hemoptysis, obstruction, pain)    2. Painful bone metastases (single fraction of 8 Gy or fractionated regimens)    3. Spinal cord compression (emergency treatment)    4. Brain metastases (whole brain RT or stereotactic radiosurgery)    5. Superior vena cava syndrome   **Small Cell Lung Cancer (SCLC)**   1. **Limited-Stage Disease**    1. Concurrent chemoradiation (early initiation preferred)    2. Accelerated hyperfractionation (twice-daily treatment) shows best outcomes but is logistically challenging 2. **Prophylactic Cranial Irradiation (PCI)**    1. Reduces incidence of brain metastases    2. Improves overall survival in limited-stage with good response to initial therapy    3. Considered but more controversial in extensive-stage disease 3. **Consolidative Thoracic RT**   May benefit selected extensive-stage patients with good response to chemotherapy  **Special Situations**   1. **Re-irradiation**    1. For recurrence in previously irradiated areas    2. Often uses SBRT techniques to minimize normal tissue toxicity 2. **Combination with Immunotherapy**    1. Emerging evidence for potential synergistic effects    2. Sequential or concurrent approaches under investigation 3. **Brachytherapy**    1. Endobronchial brachytherapy for airway obstruction    2. Less commonly used with advancement of external beam techniques   The selection of radiotherapy technique, dose, fractionation, and timing depends on tumor location, size, proximity to critical structures, patient performance status, and treatment goals (curative vs. palliative). |
| 1. **What is immunotherapy?** | Immunotherapy is a treatment approach that harnesses the body's own immune system to fight cancer. Unlike traditional cancer treatments that directly target cancer cells (like chemotherapy or targeted therapy), immunotherapy works by enhancing or restoring the immune system's ability to recognize and attack cancer cells.  In the context of lung cancer, immunotherapy has become a cornerstone treatment, particularly for advanced disease. Here's how it works and its key aspects:  **Core Mechanisms of Cancer Immunotherapy**   1. Immune Checkpoint Inhibitors: The most widely used form of immunotherapy for lung cancer. These drugs block proteins (checkpoints) that cancer cells use to evade immune detection.    1. PD-1/PD-L1 inhibitors (pembrolizumab, nivolumab, atezolizumab, cemiplimab, durvalumab)    2. CTLA-4 inhibitors (ipilimumab) 2. Cancer Vaccines: Stimulate the immune system to recognize cancer cells (less established in lung cancer) 3. Adoptive Cell Therapy: Engineering immune cells (like T cells) to better recognize and attack cancer cells (experimental in lung cancer)   **How Immunotherapy Works for Lung Cancer**   1. Cancer cells often express proteins (like PD-L1) that act as "off switches" when they interact with immune cells 2. Checkpoint inhibitors block these interactions, allowing immune cells to remain active 3. This reactivates the immune system to recognize and attack cancer cells 4. The response can be durable, sometimes lasting years after treatment ends   **When Immunotherapy Is Used in Lung Cancer**   1. First-line treatment for metastatic NSCLC with high PD-L1 expression (≥50%) 2. Combined with chemotherapy for metastatic NSCLC regardless of PD-L1 status 3. Consolidation therapy after chemoradiation for unresectable Stage III NSCLC 4. Combined with chemotherapy for extensive-stage small cell lung cancer 5. Adjuvant therapy for early-stage NSCLC (emerging role)   **Biomarkers for Immunotherapy Response**   1. PD-L1 expression: Higher levels generally correlate with better response 2. Tumor Mutational Burden (TMB): Tumors with many mutations may respond better 3. Microsatellite Instability (MSI): Rare in lung cancer but predictive of response   **Unique Aspects of Immunotherapy**   1. Delayed response: May take longer to see effects compared to other treatments 2. Pseudoprogression: Tumors may appear to grow before shrinking 3. Durable responses: Some patients experience long-term disease control 4. Immune-related adverse events: Side effects related to immune system activation 5. Different response patterns: Standard response criteria may not fully capture benefits   Immunotherapy has transformed the treatment landscape for lung cancer, offering new hope for patients with advanced disease and the potential for long-term survival for some patients who previously had limited options. |
| **Topic: Prognosis** | |
| 1. **What is the frequency of follow-up after lung cancer surgery?** | The frequency of follow-up after lung cancer surgery follows a risk-based approach, with more frequent visits in the early post-operative period when recurrence risk is highest. Here's a typical follow-up schedule based on current guidelines:  **First 2 Years (Highest Risk Period)**   1. Clinical visits with history and physical exam: Every 3-6 months 2. CT scanning: Every 3-6 months    1. Low-dose CT chest preferred    2. Some protocols include upper abdomen to assess adrenals and liver   **Years 3-5 (Moderate Risk Period)**   1. Clinical visits: Every 6 months 2. CT scanning: Every 6-12 months   **Beyond 5 Years (Lower Risk Period)**   1. Clinical visits: Annually 2. CT scanning: Annually 3. Consider transition to lung cancer screening protocols for long-term survivors   **Additional Components of Follow-up**   1. Pulmonary function tests (PFTs): Baseline post-surgery and as clinically indicated 2. Smoking cessation support: Ongoing for current smokers 3. Survivorship care: Addressing treatment-related toxicities and psychosocial needs 4. Specific testing based on symptoms: Additional imaging or procedures as needed if new symptoms develop   **Special Considerations**   1. Higher-stage disease: May warrant more frequent surveillance 2. Adenocarcinoma histology: Sometimes monitored more closely due to risk of brain metastases 3. Brain MRI: Not routinely recommended but considered for adenocarcinoma or higher-stage disease 4. PET/CT: Generally not used for routine surveillance but may be used to evaluate concerning findings 5. Tumor markers: Not recommended for routine surveillance   The frequency and intensity of follow-up should be individualized based on:   1. Initial stage of disease 2. Surgical margins 3. Patient's overall health status 4. Ability to tolerate potential salvage treatments 5. Patient preference after discussion of benefits and limitations   This surveillance schedule aims to detect recurrence or new primary lung cancers at a stage where additional curative-intent treatment may still be possible. |
| 1. **What should be done if a new nodule is found during follow-up?** | When a new nodule is found during follow-up after lung cancer surgery, a systematic evaluation is required to determine its nature and appropriate management. Here's the recommended approach:  **Initial Evaluation**   1. **Review Previous Imaging**    1. Compare with all prior imaging to confirm it's truly new    2. Assess growth rate if it was previously present but subtle    3. Review original surgical/pathology reports to understand the initial cancer 2. **Characterize the Nodule**    1. Location (same lobe, different lobe, contralateral lung)    2. Size (precise measurements in all dimensions)    3. Morphology (solid, part-solid, ground-glass)    4. Margins (smooth, lobulated, spiculated)    5. Density (HU measurements)    6. Presence of calcification, cavitation, or fat 3. **Clinical Context Assessment**    1. Time since initial cancer treatment    2. Original cancer histology and stage    3. Patient's risk factors (smoking status, occupational exposures)    4. Presence of symptoms   **Management Approach**   1. **For Very Small Nodules (<6mm)**    1. Short-interval follow-up CT in 3 months if solid    2. Follow-up in 6 months if pure ground-glass opacity    3. Apply Fleischner Society or NCCN guidelines based on risk factors 2. **For Larger Nodules (≥6mm) or Growing Nodules**    1. Consider PET/CT imaging to assess metabolic activity    2. Brain MRI if metastatic disease is suspected    3. Consider non-surgical biopsy (CT-guided or bronchoscopic)   **Decision-Making Framework**   1. **Suspected second primary:** More likely if:    1. Long interval since initial cancer    2. Different lobe/contralateral location    3. Ground-glass or part-solid appearance    4. Slow growth rate 2. **Suspected recurrence/metastasis:** More likely if:    1. Short interval since initial cancer    2. Same lobe location    3. Solid appearance    4. Rapid growth    5. Multiple nodules   **Management Options**   1. **Surgical Resection**    1. Consider for suspected second primary when feasible    2. Anatomic resection (lobectomy) or sublobar resection depending on:       1. Nodule characteristics       2. Location       3. Patient's remaining lung function 2. **Stereotactic Body Radiation Therapy (SBRT)**    1. Alternative for patients who cannot undergo surgery    2. Appropriate for isolated recurrence in some cases 3. **Systemic Therapy**    1. For suspected widespread recurrence/metastatic disease    2. Consider molecular testing if metastatic disease is confirmed 4. **Active Surveillance**    1. For indeterminate small nodules    2. Sequential imaging at appropriate intervals 5. **Multidisciplinary Discussion**    1. All cases should be discussed in a tumor board setting    2. Input from thoracic surgery, radiation oncology, medical oncology, and pulmonology   The approach should be individualized based on the patient's overall health status, preferences, and goals of care. The decision between treating aggressively versus surveillance involves balancing the risks of undertreatment against unnecessary procedures for benign findings. |
| 1. **How to determine whether lung cancer recurs during follow-up?** | Determining whether lung cancer has recurred during follow-up requires a systematic approach using multiple clinical and diagnostic tools. Here's how recurrence is detected and confirmed:  **Clinical Surveillance**   1. **Symptom Assessment**    1. New or worsening cough    2. Unexplained weight loss    3. Chest or bone pain    4. Shortness of breath    5. Hemoptysis    6. Neurological symptoms (headache, seizures, weakness)    7. Fatigue not explained by other causes 2. **Physical Examination**    1. New supraclavicular or cervical lymphadenopathy    2. Chest wall abnormalities at previous surgical site    3. Focal neurological findings    4. Hepatomegaly    5. New pleural effusion   **Imaging Surveillance**   1. **Regular CT Scanning**    1. New pulmonary nodules or masses    2. Enlarging lymph nodes (hilar, mediastinal)    3. Pleural thickening or effusion    4. Local changes at surgical site    5. Growth of previously stable nodules 2. **Additional Imaging When Indicated**    1. PET/CT: To assess metabolic activity of suspicious findings    2. Brain MRI: For suspected brain metastases, particularly in adenocarcinoma    3. Bone scan: When bone metastases are suspected    4. Abdominal CT/MRI: For evaluation of liver or adrenal lesions   **Confirmation of Recurrence**   1. **Tissue Confirmation**    1. CT-guided biopsy: For accessible lesions    2. Bronchoscopy: For central lesions or endobronchial involvement    3. EBUS/EUS: For mediastinal lymphadenopathy    4. Surgical biopsy: When less invasive methods are inadequate    5. Liquid biopsy: Detection of circulating tumor DNA (emerging approach) 2. **Distinguishing Between Types of Recurrence**    1. Local recurrence: At or near the original tumor site    2. Regional recurrence: Involving regional lymph nodes    3. Distant metastasis: New lesions in distant organs    4. Second primary lung cancer: Distinct new primary (different histology or long disease-free interval)   **Diagnostic Challenges**   1. **Post-treatment Changes vs. Recurrence**    1. Radiation fibrosis can mimic recurrence    2. Post-surgical changes may be difficult to interpret    3. Distinguishing features:       1. Stability over time (suggests benign)       2. Progressive enlargement (suggests recurrence)       3. Metabolic activity on PET (high SUV suggests recurrence) 2. **Molecular Analysis**    1. Comparison with original tumor    2. New actionable mutations may emerge in recurrent disease 3. **Biomarkers**    1. Rising tumor markers (CEA, CYFRA 21-1) may indicate recurrence in some patients    2. Not sufficiently sensitive or specific for routine surveillance   A multidisciplinary approach involving thoracic surgeons, radiologists, pulmonologists, and oncologists helps determine whether suspicious findings represent true recurrence versus treatment-related changes or new primary disease. The distinction is crucial as it guides subsequent management strategies. |
| 1. **How to manage lifestyle after lung cancer surgery?** | **Managing Lifestyle After Lung Cancer Surgery**  **Immediate Post-Surgical Period (1-3 Months)**  **Physical Activity**   1. Gradual return to activity: Start with short walks, gradually increasing distance 2. Breathing exercises: Continue respiratory exercises taught by respiratory therapists 3. Avoid heavy lifting: Typically restrict lifting >10 lbs for 6-8 weeks 4. Listen to your body: Rest when tired, but avoid becoming completely sedentary   **Wound Care**   1. Keep incision clean and dry 2. Watch for signs of infection (redness, warmth, drainage, fever) 3. Attend follow-up appointments for staple/suture removal   **Pain Management**   1. Take prescribed pain medications as directed 2. Transition to over-the-counter options when appropriate 3. Use pillows for positioning to minimize discomfort during sleep   **Long-Term Lifestyle Adjustments**  **Smoking Cessation**   1. Absolute priority: Quitting smoking reduces recurrence risk by 30-40% 2. Utilize smoking cessation resources (medications, counseling, support groups) 3. Avoid secondhand smoke exposure   **Physical Activity and Exercise**   1. Regular exercise: Aim for at least 150 minutes of moderate activity weekly 2. Pulmonary rehabilitation: Consider formal program if experiencing significant shortness of breath 3. Strength training: Begin once cleared by surgeon (typically 2-3 months post-surgery) 4. Balance aerobic exercise with strength training and flexibility   **Nutrition**   1. Anti-inflammatory diet: Emphasize fruits, vegetables, whole grains, lean proteins 2. Maintain healthy weight: Avoid unintentional weight loss or excessive weight gain 3. Adequate protein intake: Supports healing and maintains muscle mass 4. Consider nutritional counseling if experiencing significant weight loss or appetite issues   **Lung Health**   1. Avoid respiratory irritants: Pollution, chemical fumes, smoke 2. Vaccination: Stay current with flu, pneumonia, and COVID-19 vaccines 3. Prompt attention to respiratory infections   **Psychological Well-being**   1. Acknowledge emotional challenges: Fear of recurrence is normal 2. Support groups: Connect with other lung cancer survivors 3. Professional help: Consider counseling if experiencing persistent anxiety or depression 4. Mindfulness practices: Meditation, deep breathing exercises, yoga   **Practical Daily Adjustments**  **Energy Conservation**   1. Prioritize important activities 2. Plan rest periods throughout the day 3. Organize living space to minimize unnecessary exertion   **Weather Considerations**   1. Avoid temperature extremes 2. Use scarves/masks in cold weather to warm inhaled air 3. Limit outdoor activity during high pollution days   **Work Re-entry**   1. Gradual return when cleared by physician 2. Consider modified duties initially 3. Communicate limitations to employer   **Monitoring and Follow-up**  **Self-monitoring**   1. Track any new symptoms 2. Monitor weight changes 3. Keep a symptom diary if needed   **Medical Follow-up**   1. Adhere to recommended follow-up schedule 2. Prepare questions before appointments 3. Report new or worsening symptoms promptly   **Building Resilience**   1. Set realistic expectations for recovery 2. Celebrate milestones and progress 3. Focus on aspects of health within your control 4. Connect with cancer survivorship resources   These lifestyle adjustments can significantly improve quality of life after lung cancer surgery and may help reduce the risk of recurrence. Each person's recovery is unique, so work closely with your healthcare team to develop a personalized plan that addresses your specific needs and circumstances. |
| 1. **Whether the focus of follow-up for long-term survivors (e.g., more than 5 years after surgery) needs to be adjusted?** | For long-term lung cancer survivors who are more than 5 years post-surgery, the focus of follow-up does indeed need to be adjusted. This shift reflects both the changing risk profile and the evolving needs of survivors.  **Changing Focus in Long-term Surveillance**  **Medical Surveillance Adjustments**   1. Recurrence monitoring: While still important, the intensity typically decreases as the risk of recurrence drops significantly after 5 years 2. Second primary lung cancers: Becomes a more prominent concern, with an annual risk of 1-2% that persists indefinitely 3. Transition to screening protocols: Consider moving to lung cancer screening protocols similar to those for high-risk individuals without prior cancer   **Expanded Health Priorities**   1. Late treatment effects: Monitoring for delayed complications of surgery, radiation, or systemic therapy    1. Cardiac toxicity from certain chemotherapies    2. Pulmonary fibrosis from radiation    3. Chronic pain syndromes 2. General preventive healthcare: Often neglected during active cancer treatment    1. Age-appropriate screenings for other cancers    2. Management of comorbidities (COPD, heart disease, diabetes)    3. Bone health assessment, particularly after certain treatments   **Psychosocial Considerations**   1. Shift from fear of recurrence to long-term survivorship challenges 2. Address lingering psychological impacts: "New normal" adjustment, PTSD, survivor's guilt 3. Vocational and financial issues: Return to work challenges, insurance concerns, retirement planning   **Practical Recommendations for Long-term Follow-up**   1. **Imaging frequency:**    1. Annual low-dose CT chest (rather than every 6 months)    2. Consider risk-adapted approach based on original stage and histology 2. **Clinical visits:**    1. Annually with oncology team    2. Consider transition to shared-care model with primary care physician 3. **Functional assessment:**    1. Periodic pulmonary function testing    2. Exercise capacity evaluation    3. Quality of life assessment 4. **Survivorship care plans:**    1. Comprehensive documentation of treatment history    2. Clear guidelines for ongoing surveillance    3. Delineation of responsibilities between specialists and primary care 5. **Health promotion:**    1. Continued emphasis on smoking cessation/avoidance    2. Physical activity guidance specific to lung capacity    3. Nutritional counseling for long-term health   The adjustment of follow-up for long-term survivors should be individualized based on the patient's specific risk factors, treatment history, and personal preferences. A multidisciplinary survivorship clinic model can be especially valuable for addressing the complex needs of this population. |
| 1. **How to manage long-term side effects after treatment, such as lung function damage, radiation pneumonitis, etc.** | **Managing Long-term Side Effects After Lung Cancer Treatment**  **Lung Function Damage**  **Assessment**   1. Regular pulmonary function tests (PFTs) to monitor changes 2. 6-minute walk tests to assess functional capacity 3. Oxygen saturation monitoring during rest and activity   **Management Strategies**   1. **Pulmonary rehabilitation programs**     1. Supervised exercise training    2. Breathing techniques and airway clearance    3. Energy conservation strategies 2. **Medication approaches**     1. Bronchodilators for airflow limitation    2. Inhaled corticosteroids for inflammatory components    3. Oxygen supplementation when indicated 3. **Lifestyle adjustments** 4. Graduated exercise program appropriate to capacity 5. Avoidance of respiratory irritants 6. Breathing exercises and techniques 7. Vaccination against influenza and pneumococcal pneumonia   **Radiation Pneumonitis and Fibrosis**  **Radiation Pneumonitis (Early)**   1. **Monitoring:** Typically occurs 2-6 months post-radiation 2. **Treatment:**     1. Corticosteroids (prednisone) for symptomatic cases    2. Tapering schedule over 6-12 weeks    3. Prophylactic antibiotics during steroid treatment 3. **Supportive care:**     1. Cough suppressants for severe cough    2. Adequate hydration    3. Rest during acute phases   **Radiation Fibrosis (Late)**  **Management:**   - 1. Limited reversibility, focus on symptom management   2. Pulmonary rehabilitation to maximize remaining function   3. Consideration of antifibrotic medications in severe cases (pirfenidone, nintedanib)   4. Supplemental oxygen if hypoxemia is present   **Chronic Pain Syndromes**  **Post-thoracotomy Pain**   1. **Multimodal approach:**     1. Regular assessment using pain scales    2. NSAIDs and acetaminophen as first-line options    3. Neuropathic pain medications (gabapentin, pregabalin)    4. Topical agents (lidocaine patches)    5. Limited use of opioids for severe pain 2. **Interventional options:**     1. Intercostal nerve blocks    2. Thoracic epidural injections    3. Neurolysis procedures for refractory cases 3. **Non-pharmacological approaches:**     1. Physical therapy    2. TENS units    3. Heat/cold therapy    4. Acupuncture   **Cardiovascular Complications**  **Radiation-induced Heart Disease**   1. Regular cardiovascular assessment 2. Aggressive management of cardiac risk factors 3. Echocardiogram every 1-2 years if heart was in radiation field 4. Early cardiology referral for new symptoms   **Chemotherapy-related Cardiac Toxicity**   1. Baseline and periodic cardiac function assessment 2. ACE inhibitors or beta-blockers for early dysfunction 3. Heart-healthy lifestyle modifications   **Esophageal Complications**  **Esophageal Stricture**   1. Periodic barium swallow studies if symptomatic 2. Endoscopic dilation procedures 3. Dietary modification and nutritional support 4. Proton pump inhibitors to reduce reflux   **Chronic Esophagitis**   1. Dietary adjustments (soft, non-irritating foods) 2. Swallowing therapy 3. Medication for symptom management   **Neurocognitive Effects**  **Post-whole Brain Radiation**   1. Cognitive assessment and monitoring 2. Cognitive rehabilitation strategies 3. Memory aids and compensatory strategies 4. Consideration of medications (memantine)   **Fatigue Management**   1. Structured exercise program (even short, gentle sessions) 2. Energy conservation techniques 3. Sleep hygiene optimization 4. Screening for and treatment of contributing factors:    1. Anemia    2. Hypothyroidism    3. Depression    4. Sleep apnea   **Comprehensive Approach**  **Multidisciplinary Management**   1. Pulmonologist for lung-specific issues 2. Pain specialist for chronic pain 3. Cardiologist for cardiac complications 4. Physical and occupational therapy 5. Nutritionist for dietary support 6. Mental health professional   **Patient Self-management**   1. Symptom diary to track patterns and triggers 2. Pacing activities to prevent exhaustion 3. Learning warning signs requiring medical attention 4. Participation in support groups with other survivors   **Communication with Healthcare Team**   1. Regular follow-up with treatment team 2. Bringing detailed symptom descriptions to appointments 3. Discussing quality of life impacts of side effects 4. Setting realistic goals for symptom management   Long-term side effects often require ongoing management rather than expecting complete resolution. The focus should be on maximizing function, minimizing symptom burden, and maintaining quality of life through a combination of medical interventions and self-management strategies. |
| **Topic: Case** | |
| 1. **A 68-year-old male patient, with a 40-year history of smoking, has been experiencing cough and blood-tinged sputum for 3 months. Chest X-ray showed an irregular mass in the right upper lung with a rough edge; CT scan suggested that the mass invaded the pleura and mediastinal lymph nodes were enlarged. Lung cancer is initially considered, and bronchoscopy and biopsy are recommended to confirm the pathology. What further tests does the patient need to confirm the diagnosis?** | For this 68-year-old male patient with a concerning presentation of lung cancer, further diagnostic testing should include:  **Confirming the Diagnosis**   1. **Bronchoscopy with biopsy** (already recommended)    1. This is essential for histological confirmation and molecular testing    2. Enables tissue sampling from the mass and potentially from mediastinal lymph nodes (via EBUS) 2. **CT-guided percutaneous needle biopsy**    1. Alternative or complementary approach if the lesion is peripheral and difficult to access via bronchoscopy    2. May provide larger tissue samples for comprehensive molecular testing   **Staging Workup**   1. **PET/CT scan**    1. Evaluates metabolic activity of the primary tumor and assesses for distant metastases    2. Helps differentiate between benign and malignant lymphadenopathy    3. Identifies occult metastatic disease not detected on conventional imaging 2. **Brain MRI with contrast**    1. Essential for complete staging, particularly for upper lobe tumors    2. Detects brain metastases that may be present even in asymptomatic patients 3. **Pulmonary function tests (PFTs)**    1. Assesses baseline lung function    2. Determines operability if surgical resection is considered    3. May include DLCO, spirometry, and lung volumes   **Molecular and Pathological Assessment**   1. **Comprehensive pathological examination**    1. Histological typing (NSCLC vs. SCLC, adenocarcinoma vs. squamous cell, etc.)    2. Immunohistochemistry for tumor classification 2. **Molecular testing (particularly if NSCLC is confirmed)**    1. EGFR, ALK, ROS1, BRAF, NTRK testing    2. Extended molecular panel including MET, RET, KRAS    3. PD-L1 expression testing for immunotherapy planning 3. **Mediastinal staging**    1. Endobronchial ultrasound (EBUS) with transbronchial needle aspiration    2. Mediastinoscopy if EBUS is negative but high suspicion remains for N2/N3 disease   Given the patient's presentation with pleural invasion and mediastinal lymphadenopathy, these comprehensive tests are essential to confirm the diagnosis, determine the precise stage, and identify potential treatment options tailored to his specific tumor characteristics. |
| 1. **A 55-year-old female patient, diagnosed with advanced non-small cell lung cancer (NSCLC), with EGFR gene mutation positive. She has received gefitinib targeted therapy, but recently developed drug resistance and tumor progression. How to adjust the next treatment plan?** | For a 55-year-old female patient with EGFR-positive advanced NSCLC who has developed resistance to first-line gefitinib, several evidence-based approaches should be considered:  **First, Determine the Mechanism of Resistance**  **Repeat biopsy or liquid biopsy (circulating tumor DNA) to identify the resistance mechanism:**   1. Test specifically for T790M mutation (most common resistance mechanism, ~60% of cases) 2. Assess for other resistance mechanisms (MET amplification, HER2 amplification, small cell transformation)   **Treatment Options Based on Resistance Mechanism**   1. **If T790M mutation is detected:**    1. Osimertinib (third-generation EGFR-TKI) is the standard of care    2. Offers superior blood-brain barrier penetration if CNS metastases are present    3. Median progression-free survival of 10-12 months after gefitinib resistance 2. **If T790M negative:**    1. Platinum-based chemotherapy (carboplatin/cisplatin) plus pemetrexed    2. Consider adding bevacizumab (anti-angiogenic) to chemotherapy    3. Immunotherapy combinations may be considered after chemotherapy, though benefits in EGFR+ disease are less pronounced 3. **If MET amplification is detected:**    1. Consider EGFR-TKI plus MET inhibitor (crizotinib, capmatinib, or tepotinib)    2. Clinical trials of combination targeted therapies if available 4. **If transformed to small cell lung cancer:**   Switch to small cell lung cancer treatment protocols (platinum/etoposide)  **Additional Considerations**   1. **Evaluate for oligoprogressive disease:**    1. If progression is limited to 1-3 sites, consider local therapy (radiation, surgery) to those sites    2. Continue gefitinib for systemic control if most disease remains controlled 2. **Clinical trial options:**    1. Fourth-generation EGFR inhibitors    2. Novel combination approaches    3. Antibody-drug conjugates 3. **Re-evaluate overall disease burden and patient performance status:**    1. If the patient has maintained good performance status, more aggressive approaches may be appropriate    2. If deteriorating, consider quality of life and symptom management priorities   The most likely next step would be osimertinib if T790M mutation is detected, or platinum-based chemotherapy if T790M is negative. This approach should be discussed in a multidisciplinary tumor board to tailor treatment to her specific clinical situation and preferences. |
| 1. **A 70-year-old male patient, on the 3rd day after lung cancer surgery, developed pleural effusion and poor drainage. Body temperature was 37.8°C, heart rate was 90 beats/min, respiratory rate was 22 breaths/min, and the drainage tube was blocked. The patient complained of chest tightness. How to deal with the blocked drainage tube?** | This 70-year-old male patient is showing concerning signs of a post-operative complication related to a blocked drainage tube. The situation requires prompt intervention to prevent further deterioration. Here's how to manage the blocked drainage tube:  **Immediate Management**   1. **Assessment of the drainage system:**    1. Visually inspect the entire tube system for kinks, clots, or compression    2. Check for fibrin plugs or blood clots at the insertion site    3. Ensure the drainage chamber is properly positioned below chest level 2. **Attempt to restore patency:**    1. Milk/strip the tube gently if hospital protocol allows    2. Change the patient's position to mobilize fluid    3. If hospital policy permits, consider gentle flushing with sterile saline under strict aseptic technique 3. **If tube remains blocked:**    1. Immediate replacement of the chest tube may be necessary    2. This should be done by a qualified thoracic surgeon or practitioner   **Additional Management Steps**   1. **Urgent chest imaging:**    1. Obtain a bedside chest X-ray or ultrasound to assess the volume and location of pleural fluid    2. Rule out pneumothorax or hemothorax 2. **Monitor vital signs and symptoms:**    1. The patient's elevated temperature, tachycardia, and tachypnea suggest developing infection or inflammatory response    2. Continuous monitoring of oxygen saturation    3. Consider arterial blood gas analysis if respiratory distress worsens 3. **Supportive measures:**    1. Supplemental oxygen as needed    2. Semi-Fowler's position (30-45° elevation) to improve breathing mechanics    3. Pain management to ensure adequate ventilation 4. **Investigate and treat underlying cause:**    1. Blood cultures if infection is suspected    2. Consider empiric antibiotics if signs of empyema are present   The blocked chest tube requires urgent attention as it can rapidly lead to respiratory compromise, especially in an elderly post-surgical patient. The combination of fever, tachycardia, tachypnea, and subjective chest tightness indicates the patient may be developing a significant post-operative complication that needs immediate intervention. |
| 1. **A 48-year-old female patient, newly diagnosed with advanced lung cancer, is feeling low and refusing treatment. The patient has obvious anxiety and depression, worrying about the side effects of treatment and family burden. How to help the patient rebuild treatment confidence?** | Helping this patient navigate her emotional response to a lung cancer diagnosis requires sensitivity, clear information, and practical support. Here's how to approach rebuilding her confidence in treatment:  **Acknowledge and Validate Her Feelings**  Begin by creating a safe space where she can express her concerns without judgment. Her anxiety, depression, and worries about treatment side effects and burdening her family are completely understandable responses to a life-changing diagnosis. Validate that these feelings are normal and that many patients experience similar emotions.  **Provide Psychological Support**   1. Offer referral to a psycho-oncologist or therapist with experience in cancer care 2. Consider short-term anxiety/depression medication if clinically appropriate 3. Introduce her to support groups where she can connect with others facing similar challenges 4. Suggest mindfulness techniques or relaxation exercises that may help manage anxiety   **Address Treatment Concerns with Clear Information**   1. Explain treatment options using plain language, focusing on both benefits and risks 2. Discuss specific side effects she's concerned about and the strategies available to manage them 3. Share success stories and statistics about treatment effectiveness when appropriate 4. Use visual aids or decision-making tools to help her process information 5. Consider a staged approach to treatment that might feel less overwhelming   **Family Involvement and Practical Support**   1. With her permission, involve family members in discussions to address concerns about being a burden 2. Connect her with a social worker who can discuss practical matters (financial resources, home support) 3. Explore what specific supports would help her feel less burdensome to her family 4. Arrange for family counseling if family dynamics are contributing to her distress   **Build a Relationship of Trust**   1. Schedule adequate time for appointments to avoid feeling rushed 2. Ensure continuity of care with the same healthcare providers when possible 3. Be honest but hopeful in all communications 4. Acknowledge uncertainties while emphasizing what can be controlled 5. Respect her autonomy while gently encouraging engagement with treatment   **Consider a Multidisciplinary Approach**   1. Involve palliative care early (focusing on quality of life, not just end-of-life care) 2. Coordinate between oncology, nursing, social work, psychology, and primary care 3. Consider complementary approaches that might help her feel more in control (nutrition counseling, exercise support, integrative medicine where appropriate)   **Small Steps Forward**  Finally, help her identify small, manageable steps toward engagement with treatment rather than pushing for immediate full commitment. This might include meeting with one specialist at a time, starting with less invasive interventions, or attending a single support group meeting.  By combining emotional support with practical information and respecting her autonomy, you can help this patient process her diagnosis and rebuild confidence in the treatment process at her own pace. |
| 1. **A 65-year-old male patient, diagnosed with locally advanced non-small cell lung cancer, is not suitable for surgical resection. He has completed concurrent chemoradiotherapy, but developed radiation pneumonitis and shortness of breath. How to relieve the symptoms of radiation pneumonitis?** | Managing radiation pneumonitis in this 65-year-old male with locally advanced NSCLC requires a comprehensive approach focused on reducing inflammation and improving respiratory symptoms. Here's how to address his radiation pneumonitis:  **Pharmacological Management**   1. **Corticosteroids - First-line treatment**    1. Start with prednisone 40-60 mg daily (or equivalent)    2. Continue initial dose until symptom improvement (typically 1-2 weeks)    3. Gradually taper over 6-12 weeks to avoid symptom rebound    4. Monitor for steroid-related side effects (blood glucose, blood pressure, etc.) 2. **Prophylactic antimicrobials**    1. Consider prophylaxis against Pneumocystis jirovecii pneumonia during prolonged steroid use    2. Monitor for opportunistic infections 3. **Supportive medications**    1. Antitussives for persistent cough    2. Bronchodilators if there's a component of bronchospasm    3. Consider diuretics if fluid overload is contributing to symptoms   **Respiratory Support**   1. **Oxygen therapy**    1. Supplemental oxygen for hypoxemia    2. Titrate to maintain SpO2 > 92% (or patient-specific goal)    3. Consider home oxygen evaluation if persistently hypoxemic 2. **Pulmonary rehabilitation**    1. Breathing exercises and techniques    2. Energy conservation strategies    3. Gradual, supervised exercise program   **Monitoring and Follow-up**   1. **Regular clinical assessment**    1. Symptom monitoring (dyspnea scale, cough, fatigue)    2. Physical examination    3. Pulse oximetry 2. **Imaging**    1. Follow-up chest imaging to assess response to treatment    2. Consider high-resolution CT to evaluate extent of pneumonitis 3. **Pulmonary function tests**   Baseline and follow-up to track improvement or deterioration  **Lifestyle Modifications**   1. **Activity pacing**    1. Balance activity with rest    2. Energy conservation techniques 2. **Environmental considerations**    1. Avoid respiratory irritants (smoke, pollution, strong fumes)    2. Maintain optimal indoor air quality 3. **Nutritional support**    1. Proper hydration    2. Anti-inflammatory diet    3. Small, frequent meals if dyspnea affects eating   For severe or refractory cases, consider:   1. Hospitalization for intensive monitoring and treatment 2. Higher doses of corticosteroids 3. Second-line immunosuppressive agents in consultation with pulmonology 4. Mechanical ventilatory support if respiratory failure develops   This multifaceted approach addresses both the underlying inflammation and the symptomatic manifestations of radiation pneumonitis, with the goal of improving quality of life while monitoring for potential complications. |

**Supplementary Table 2b:** Gemini Advanced 2.0 Responses to Lung Cancer Questions

| **Question** | **Gemini Advanced 2.0** |
| --- | --- |
| **Topic: Overview** | |
| 1. **What is lung cancer?** | Lung cancer is a disease characterized by the uncontrolled growth of abnormal cells that begin in one or both lungs. These cells can form tumors and interfere with the lung's ability to function properly. Here's a breakdown of key aspects:  **Key Points:**  **Uncontrolled Cell Growth:**   1. Lung cancer arises when cells in the lungs undergo genetic changes (mutations) that cause them to divide and multiply uncontrollably. 2. This uncontrolled growth leads to the formation of tumors.   **Types of Lung Cancer:**  The two main types are:   - 1. Non-small cell lung cancer (NSCLC): This is the most common type, accounting for the majority of lung cancer cases.   2. Small cell lung cancer (SCLC): This type tends to grow and spread more rapidly.   **Causes and Risk Factors:**   1. Smoking: The leading cause of lung cancer. 2. Secondhand smoke: Exposure to smoke from others. 3. Radon gas: A naturally occurring radioactive gas. 4. Asbestos: Exposure to this mineral. 5. Other carcinogens: Exposure to certain chemicals and pollutants. 6. Family history: A genetic predisposition.   **Symptoms:**  Symptoms may not appear until the cancer is advanced. Common symptoms include:   - 1. Persistent cough that worsens.   2. Coughing up blood.   3. Shortness of breath.   4. Chest pain.   5. Hoarseness.   6. Unexplained weight loss.   **Spread (Metastasis):**  Lung cancer can spread (metastasize) to other parts of the body, such as the brain, bones, and liver.  It's important to remember that early detection is crucial for improving outcomes. If you have any concerns about lung cancer, it's essential to consult with a healthcare professional. |
| 1. **What are the clinical features of lung cancer?** | Lung cancer can present with a variety of clinical features, and it's important to note that early-stage lung cancer often has very few, if any, symptoms. Symptoms tend to appear or worsen as the cancer progresses. Here's a breakdown of common clinical features:  **Respiratory Symptoms:**   1. Persistent cough:   A new cough that doesn't go away or a chronic cough that worsens.   1. Coughing up blood (hemoptysis):   Even small amounts of blood in sputum should be taken seriously.   1. Shortness of breath (dyspnea):   Difficulty breathing, especially with exertion.   1. Chest pain:   Can be dull, aching, or sharp, and may worsen with deep breathing, coughing, or laughing.   1. Wheezing:   A whistling sound during breathing.   1. Hoarseness:   Changes in voice.   1. Recurrent respiratory infections:   Such as bronchitis or pneumonia that don't clear up or keep returning.  **Systemic Symptoms (Symptoms affecting the whole body):**   1. Unexplained weight loss:   Significant weight loss without trying.   1. Loss of appetite:   Reduced desire to eat   1. Fatigue:   Feeling unusually tired or weak.  **Symptoms of Metastasis (Spread of Cancer):**   1. Bone pain:   If the cancer has spread to the bones.   1. Headaches, neurological symptoms:   Such as weakness or seizures, if the cancer has spread to the brain.   1. Jaundice:   Yellowing of the skin and eyes, if the cancer has spread to the liver.   1. Swollen lymph nodes:   In the neck or collarbone area.  **Other Possible Signs:**   1. Finger clubbing:   Changes in the shape of the fingertips and nails.   1. Superior vena cava syndrome:   Swelling in the face, neck, and upper chest due to compression of the superior vena cava.   1. Paraneoplastic syndromes:   These are conditions caused by substances produced by the tumor cells, which can affect various parts of the body, including the nervous system and endocrine system.  It's crucial to remember that these symptoms can also be caused by other conditions. If you experience any of these symptoms, especially if you have risk factors for lung cancer, it's essential to consult a healthcare professional for evaluation. |
| 1. **Is lung cancer hereditary?** | While the vast majority of lung cancer cases are linked to environmental factors, particularly smoking, there is indeed a genetic component that can increase a person's risk. Here's a breakdown:  **Key Points:**   1. **Not Primarily Hereditary:**   Most lung cancers are caused by acquired genetic mutations due to factors like smoking, radon exposure, and other carcinogens. These are "somatic mutations" and are not inherited.   1. **Genetic Predisposition:**     1. However, some people inherit genetic variations that make them more susceptible to lung cancer. These are "germline mutations."    2. This means that while they don't inherit lung cancer itself, they inherit an increased risk of developing it. 2. **Factors to Consider:**     1. A family history of lung cancer, especially in individuals who have never smoked, can suggest a possible genetic predisposition.    2. Research is ongoing to identify specific genes that contribute to lung cancer risk. 3. **Important Considerations:**     1. Even with a genetic predisposition, environmental factors still play a significant role.    2. It's crucial to remember that having a genetic risk does not guarantee that someone will develop lung cancer. 4. In essence, while lung cancer is not typically considered a hereditary disease in the traditional sense, genetic factors can increase an individual's susceptibility. |
| 1. **What are the types of pathological diagnoses for lung cancer?** | Pathological diagnosis of lung cancer is crucial for determining the specific type of lung cancer, which in turn guides treatment decisions. Here's a breakdown of the key pathological diagnoses:  **Major Categories:**   1. **Non-Small Cell Lung Cancer (NSCLC):**     1. This is the most common type, accounting for the majority of lung cancer cases.    2. Subtypes of NSCLC include:       1. Adenocarcinoma:          1. Often arises in the outer regions of the lungs.          2. Frequently seen in non-smokers.          3. Molecular testing is very important for this subtype.       2. Squamous Cell Carcinoma:          1. Typically originates in the central airways.          2. Strongly associated with smoking.       3. Large Cell Carcinoma:          1. A less common subtype.          2. Diagnosed when the cancer cells don't fit clearly into the other NSCLC categories. 2. **Small Cell Lung Cancer (SCLC):**     1. Also known as "oat cell carcinoma."    2. Tends to grow and spread rapidly.    3. Strongly associated with smoking.    4. Often diagnosed at an advanced stage.   **Diagnostic Techniques:**   1. **Histopathology:**     1. Examination of tissue samples under a microscope.    2. This is the cornerstone of lung cancer diagnosis. 2. **Cytology:**     1. Examination of cells from sputum or fluid samples.    2. Can be used for initial screening or to confirm a diagnosis. 3. **Immunohistochemistry (IHC):**     1. Uses antibodies to identify specific proteins in cancer cells.    2. Helps to distinguish between different subtypes of lung cancer. 4. **Molecular Testing:**     1. Analysis of genetic mutations in cancer cells.    2. Essential for identifying targets for targeted therapies, particularly in adenocarcinoma.    3. This includes techniques like:       1. PCR (Polymerase Chain Reaction)       2. NGS (Next-Generation Sequencing)       3. FISH (Fluorescence in situ hybridization)   **Important Considerations:**   1. The 2021 World Health Organization (WHO) Classification of Thoracic Tumours emphasizes the importance of integrating morphological, immunohistochemical, and molecular findings for accurate diagnosis. 2. It is very important to get enough tissue from the tumor to allow for all the testing that is needed, especially for molecular testing.    1. In summary, pathological diagnosis of lung cancer involves a combination of techniques to accurately identify the specific type of cancer and its molecular characteristics. This information is critical for guiding personalized treatment strategies. |
| 1. **What are the characteristics of different types of lung cancer?** | Lung cancer is broadly categorized into two main types: non-small cell lung cancer (NSCLC) and small cell lung cancer (SCLC), each with distinct characteristics. Here's a breakdown:  **Non-Small Cell Lung Cancer (NSCLC):**   1. Prevalence:   This is the most common type, accounting for approximately 80-85% of lung cancer cases.   1. Growth and Spread:   Generally grows and spreads more slowly than SCLC.   1. Subtypes:    1. Adenocarcinoma:       1. Most common NSCLC subtype.       2. Often originates in the outer areas of the lungs.       3. More common in non-smokers.       4. Tends to be detected at an earlier stage than other lung cancers.    2. Squamous Cell Carcinoma:    3. Typically arises in the central airways (bronchi).    4. Strongly associated with smoking.    5. Tends to grow relatively slowly.    6. Large Cell Carcinoma:       1. Less common subtype.       2. Can occur in any part of the lung.       3. Tends to grow and spread quickly.   **Small Cell Lung Cancer (SCLC):**   1. Prevalence:   Accounts for about 10-15% of lung cancers.   1. Growth and Spread:    1. Known for its rapid growth and early spread (metastasis).    2. Often diagnosed at an advanced stage. 2. Association with Smoking:   Strongly linked to cigarette smoking.   1. Characteristics:    1. Cells appear small and "oat-shaped" under a microscope.    2. Typically starts in the central airways.    3. Responds well to chemotherapy and radiation initially, but recurrence is common. 2. Staging:   SCLC is often staged as "limited" (confined to one lung and nearby lymph nodes) or "extensive" (spread to other parts of the body).  **Key Differences Summarized:**   1. Growth Rate:    1. SCLC: Rapid.    2. NSCLC: Slower (varies by subtype). 2. Spread:    1. SCLC: Early and widespread.    2. NSCLC: Slower and more localized (initially). 3. Association with Smoking:    1. SCLC: Very strong.    2. NSCLC: Strong (especially squamous cell), but adenocarcinoma is more common in non-smokers.   Understanding these distinctions is crucial for determining the most effective treatment approach. |
| 1. **What are the common metastasis sites of lung cancer?** | Lung cancer, unfortunately, has a tendency to spread, or metastasize, to various parts of the body. Here are the most common sites:   1. **Brain:**   Brain metastases can cause a range of neurological symptoms, such as headaches, seizures, vision changes, and weakness.   1. **Bones:**   Bone metastases often lead to bone pain, and can increase the risk of fractures.   1. **Liver:**   Liver metastases can cause abdominal pain, jaundice (yellowing of the skin and eyes), and other liver-related problems.   1. **Adrenal** **glands:**   These are small glands located on top of the kidneys. Metastases to the adrenal glands may not always cause noticeable symptoms.   1. **Lymph nodes:**   Lung cancer frequently spreads to nearby lymph nodes in the chest (mediastinal lymph nodes). It also can spread to distant lymph nodes.   1. **Other** **lung:**   It is possible for lung cancer to spread to the other lung.  **Key points to remember:**   1. The spread of lung cancer can significantly impact treatment options and prognosis. 2. Early detection and treatment are crucial to help prevent or slow the spread of the disease. |
| **Topic:** **Diagnosis** | |
| 1. **What are the early symptoms of lung cancer?** | It's important to understand that early lung cancer often has very few, if any, noticeable symptoms. This is why lung cancer can be so dangerous. However, some early signs can occur, and it's crucial to be aware of them. Here are some potential early symptoms of lung cancer:   1. Persistent Cough:   A new cough that doesn't go away, or a chronic cough that changes or worsens.   1. Changes in a Chronic Cough:   If you have a long-standing cough, any changes in its frequency, intensity, or character should be checked.   1. Coughing Up Blood (Hemoptysis):   Even small amounts of blood in your sputum (phlegm) warrant immediate medical attention.   1. Chest Pain:   Chest pain that is persistent, especially if it worsens with deep breathing, coughing, or laughing.   1. Shortness of Breath:   Unexplained shortness of breath, especially with activities you used to be able to do easily.   1. Hoarseness:   changes in your voice.  **Key considerations:**   1. These symptoms can also be caused by various other conditions, such as infections. However, if you experience any of these symptoms, especially if you have risk factors for lung cancer (such as smoking), it's essential to consult a doctor.   Early detection is crucial for improving the chances of successful treatment. |
| 1. **What are the diagnostic criteria for lung cancer?** | The diagnostic criteria for lung cancer involve a multi-step process, combining clinical evaluation, imaging, and pathological confirmation. Here's a breakdown:  **Clinical Evaluation:**   1. Medical History:   Detailed history of symptoms, risk factors (smoking, exposure to carcinogens), and family history.   1. Physical Examination:   Checking for signs such as abnormal lung sounds, swollen lymph nodes, or other physical abnormalities.  **Imaging Studies:**   1. Chest X-ray:   Often the initial imaging test. May reveal a lung nodule or mass.   1. Computed Tomography (CT) Scan:   Provides more detailed images of the lungs and chest, helping to identify and characterize lung lesions.   1. Positron Emission Tomography (PET) Scan:   Used to assess the metabolic activity of lung lesions, helping to differentiate between benign and malignant tumors. Often combined with CT (PET-CT).   1. Magnetic Resonance Imaging (MRI):   May be used to evaluate the extent of tumor invasion or to assess for brain or spinal cord metastases.   1. Bronchoscopy:   A thin tube with a light and camera is inserted into the airways. This allows for visual inspection, and the ability to take tissue samples.  **Pathological Confirmation:**   1. Tissue Biopsy:   The definitive diagnosis of lung cancer requires a tissue biopsy. This can be obtained through:   - - 1. Bronchoscopy: Taking tissue samples from the airways.     2. Needle biopsy (CT-guided or ultrasound-guided): Inserting a needle through the chest wall to obtain a tissue sample.     3. Surgical biopsy: Obtaining a tissue sample during a surgical procedure.     4. Endobronchial ultrasound (EBUS): using ultrasound during a bronchoscopy to guide biopsies of lymph nodes.  1. Cytology:   Examination of cells from sputum or pleural fluid.   1. Histopathology:   Examination of the tissue sample under a microscope. This is the gold standard of diagnosis.   1. Immunohistochemistry (IHC):   Uses antibodies to identify specific proteins in the tumor cells, helping to determine the type and origin of the cancer.   1. Molecular Testing:   Testing the tissue for genetic mutations. This is vital for targeted therapies.  **Diagnostic Criteria Summary:**   1. A suspicious finding on imaging (chest X-ray, CT scan, or PET scan). 2. Pathological confirmation of malignancy through biopsy or cytology. 3. Staging evaluation to determine the extent of the disease.   It's important to note that the specific diagnostic procedures used will vary depending on the individual's symptoms, risk factors, and the findings of initial imaging studies. |
| 1. **What are the risk factors for lung cancer?** | Lung cancer risk factors can be broadly categorized, and it's important to understand that having a risk factor doesn't guarantee you'll get lung cancer, but it does increase your chances. Here's a breakdown:  **Primary Risk Factors:**   1. Smoking:    1. This is the leading cause of lung cancer. Cigarette smoking accounts for the vast majority of lung cancer deaths.    2. The risk increases with the number of cigarettes smoked and the duration of smoking.    3. Cigar and pipe smoking also increase the risk. 2. Secondhand Smoke:   Even if you don't smoke, breathing in secondhand smoke increases your risk.   1. Radon Gas:    1. Radon is a naturally occurring radioactive gas that can accumulate in homes.    2. It's the second leading cause of lung cancer. 2. Asbestos:   Exposure to asbestos, often in occupational settings, significantly increases the risk, especially in smokers.   1. Other Carcinogens:   Exposure to other cancer-causing agents, such as:   - - 1. Arsenic     2. Chromium     3. Nickel     4. Coal products     5. Silica     6. Diesel exhaust.  1. Family History:   A family history of lung cancer can increase your risk, possibly due to inherited genetic factors.   1. Radiation Therapy to the Chest:   Previous radiation therapy to the chest area can increase the risk.   1. Air Pollution:   living in areas with high air pollution can slightly increase the risk.  **Important Considerations:**   1. The risk of lung cancer is often higher when multiple risk factors are present. For example, smoking and asbestos exposure together greatly increase the risk.   Although smoking is the predominant risk factor, lung cancer can occur in people who have never smoked. |
| 1. **What are the auxiliary examinations and assessments that should be performed for lung cancer?** | When lung cancer is suspected or diagnosed, several auxiliary examinations and assessments are performed to determine the extent of the disease, guide treatment decisions, and assess the patient's overall health. These can be broadly categorized as imaging, tissue sampling, and functional assessments. Here's a breakdown:  **Imaging Studies:**   1. CT Scan (Chest, Abdomen, and Pelvis):   To evaluate the primary tumor, assess lymph node involvement, and detect distant metastases in the abdomen and pelvis.   1. PET-CT Scan:   To assess the metabolic activity of the tumor and identify areas of potential metastasis.   1. MRI (Brain, Spine):   To evaluate for brain or spinal cord metastases, especially if neurological symptoms are present.   1. Bone Scan or PET Bone Scan:   To evaluate for bone metastases, especially if the patient is experiencing bone pain.   1. Endobronchial Ultrasound (EBUS):   To visualize and biopsy mediastinal lymph nodes.   1. Mediastinoscopy:   Surgical procedure to visualize and biopsy mediastinal lymph nodes.  **Tissue Sampling and Pathological Assessments:**   1. Bronchoscopy with Biopsy/Brushings/Washings:   To obtain tissue samples from the airways.   1. CT-Guided Needle Biopsy:   To obtain tissue samples from peripheral lung lesions or mediastinal masses.   1. Pleural Fluid Cytology:   If pleural effusion is present, to examine cells in the fluid for malignancy.   1. Surgical Biopsy (Thoracoscopy or Thoracotomy):   To obtain tissue samples when other methods are inconclusive or for surgical staging.   1. Pathological Examination:   Histopathological examination of tissue samples to confirm the diagnosis and determine the subtype of lung cancer.   1. Immunohistochemistry (IHC):   To further characterize the tumor and identify specific markers.   1. Molecular Testing (Genetic Testing):   To identify specific genetic mutations (e.g., EGFR, ALK, ROS1, PD-L1) that may be targets for targeted therapies.  **Functional Assessments:**   1. Pulmonary Function Tests (PFTs):   To assess lung function and determine the patient's respiratory reserve, especially before surgery.   1. Arterial Blood Gas (ABG) Analysis:   To assess blood oxygen and carbon dioxide levels.   1. Complete Blood Count (CBC):   To evaluate blood cell counts.   1. Comprehensive Metabolic Panel (CMP):   To assess liver and kidney function.   1. Electrocardiogram (ECG):   To evaluate heart function.   1. Echocardiogram:   To evaluate heart function, especially before certain treatments.   1. Performance Status Assessment (e.g., ECOG or Karnofsky score):   To assess the patient's overall functional status and ability to tolerate treatment.   1. Nutritional Assessment:   To evaluate and address any nutritional deficiencies.  **Staging Procedures:**  Clinical and pathological staging (TNM staging) is crucial for determining the extent of the disease and guiding treatment decisions.  These examinations and assessments are crucial for accurate diagnosis, staging, and treatment planning in lung cancer. The specific tests performed will vary depending on the individual patient's clinical presentation and the suspected stage of the disease. |
| 1. **What is the role of CT scan in the diagnosis of lung cancer?** | CT scans play a crucial and multifaceted role in the diagnosis and management of lung cancer. Here's a breakdown of their key functions:  **Detection and Localization of Lung Abnormalities:**   1. Identifying Lung Nodules and Masses: CT scans are highly sensitive in detecting small lung nodules or masses that may be indicative of lung cancer, often more so than standard chest X-rays. 2. Precise Localization: They provide detailed cross-sectional images, allowing for accurate localization of lesions within the lung.   **Characterization of Lung Lesions:**   1. Size and Shape Assessment: CT scans allow for precise measurement of the size and assessment of the shape and density of lung lesions. 2. Evaluation of Lesion Characteristics: They can help differentiate between solid and ground-glass opacities, as well as identify features suggestive of malignancy, such as spiculated margins or calcifications. 3. Assessing Invasion: CT scans can show if a tumor is invading surrounding structures, such as the chest wall, mediastinum, or major blood vessels.   **Staging of Lung Cancer:**   1. Lymph Node Evaluation: CT scans can visualize mediastinal and hilar lymph nodes, helping to assess for lymph node involvement, a critical factor in lung cancer staging. 2. Detection of Distant Metastases: CT scans of the chest, abdomen, and pelvis can help detect metastases in other organs, such as the liver, adrenal glands, or bones. 3. TNM Staging: CT scan information is essential for determining the T (tumor size and extent), N (lymph node involvement), and M (distant metastasis) components of the TNM staging system.   **Guiding Biopsy Procedures:**   1. CT-Guided Needle Biopsy: CT scans are used to guide needle biopsies of lung lesions, allowing for precise targeting and minimizing the risk of complications. 2. Planning Bronchoscopy: CT scans can help plan bronchoscopy procedures by providing a roadmap of the airways and identifying target lesions.   **Monitoring Treatment Response:**   1. Evaluating Tumor Size and Regression: CT scans are used to monitor the response of lung cancer to treatment, such as chemotherapy, radiation therapy, or targeted therapy, by assessing changes in tumor size and density. 2. Detecting Recurrence: They can help detect recurrence of lung cancer after treatment.   **Key Advantages of CT Scans:**   1. High sensitivity for detecting lung abnormalities. 2. Detailed cross-sectional imaging. 3. Ability to assess surrounding structures. 4. Guidance for biopsy procedures.   In summary, CT scans are indispensable tools in the diagnosis, staging, and management of lung cancer, providing essential information for treatment planning and monitoring. |
| 1. **What is the role of genetic testing in the diagnosis of lung cancer?** | Genetic testing has become increasingly crucial in the management of lung cancer, particularly non-small cell lung cancer (NSCLC), for several key reasons. Here's a breakdown of its role:  **Identifying Targetable Mutations:**   1. Personalized Treatment:    1. Genetic testing helps identify specific genetic mutations or alterations within the tumor cells.    2. The presence of certain mutations, such as EGFR, ALK, ROS1, BRAF, MET, and NTRK, indicates that the tumor may be susceptible to targeted therapies.    3. Targeted therapies are drugs that specifically attack cancer cells with these mutations, often leading to better outcomes and fewer side effects compared to traditional chemotherapy. 2. Improving Survival:   Identifying these mutations allows for the use of more effective, personalized treatments, significantly improving patient survival rates.  **Guiding Treatment Decisions:**   1. Selecting Optimal Therapy:    1. Genetic testing helps oncologists select the most appropriate treatment strategy for each individual patient.    2. It avoids the use of ineffective therapies and helps ensure that patients receive the most beneficial treatment from the outset. 2. Predicting Response to Therapy:   In some cases, genetic testing can also help predict how a patient's tumor is likely to respond to certain therapies.  **Identifying Resistance Mechanisms:**  Monitoring Treatment Effectiveness:   1. Genetic testing can be used to monitor the effectiveness of targeted therapies. 2. If a patient's tumor stops responding to a targeted therapy, genetic testing can identify new mutations that may have developed, leading to drug resistance. 3. This information can guide the selection of alternative therapies.   **Refining Diagnosis and Prognosis:**   1. Subtype Classification:   Genetic testing can help refine the classification of lung cancer subtypes, especially in cases where the histological diagnosis is unclear.   1. Prognostic Information:   Some genetic alterations can provide prognostic information, helping to predict the likely course of the disease.  **Liquid Biopsies:**   1. Less Invasive Testing:    1. Liquid biopsies, which analyze circulating tumor DNA (ctDNA) in the blood, offer a less invasive alternative to traditional tissue biopsies.    2. They can be used to detect mutations, monitor treatment response, and identify resistance mechanisms. 2. Monitoring Minimal Residual Disease:   Liquid biopsies can also be used to detect minimal residual disease after treatment, helping to identify patients at risk of recurrence.  **In summary:**  Genetic testing has revolutionized the management of lung cancer by enabling personalized treatment, improving survival, and refining diagnosis and prognosis. It is now a standard of care for patients with advanced NSCLC. |
| **Topic:** **Treatment** | |
| 1. **What are the treatment methods for lung cancer?** | Lung cancer treatment depends on several factors, including the type of lung cancer (NSCLC or SCLC), the stage of the cancer, the patient's overall health, and their preferences. Here's an overview of common treatment methods:  **Surgery:**   1. For Early-Stage NSCLC:    1. Surgery is often the primary treatment for early-stage NSCLC.    2. Procedures may include:       1. Wedge resection: Removal of a small wedge-shaped portion of the lung.       2. Segmentectomy: Removal of a larger portion of the lung than a wedge resection.       3. Lobectomy: Removal of an entire lobe of the lung.       4. Pneumonectomy: Removal of an entire lung. 2. Purpose: To remove the tumor and any affected lymph nodes.   **Radiation Therapy:**   1. External Beam Radiation Therapy: 2. Uses high-energy X-rays to kill cancer cells. 3. Can be used alone, before surgery (neoadjuvant), or after surgery (adjuvant). 4. Can be used to treat tumors that are inoperable, or to relieve symptoms such as pain or bleeding. 5. Stereotactic Body Radiation Therapy (SBRT):    1. Delivers high doses of radiation to a small, precisely targeted area.    2. Often used for early-stage NSCLC in patients who are not candidates for surgery. 6. Prophylactic Cranial Irradiation (PCI):   Radiation given to the brain to prevent brain metastases, most often used in SCLC.  **Chemotherapy:**   1. Systemic Treatment:    1. Uses drugs to kill cancer cells throughout the body.    2. Often used for advanced NSCLC and SCLC.    3. Can be used before or after surgery, or as the primary treatment for advanced disease. 2. Combination Chemotherapy:   Often, multiple chemotherapy medications are used together.  **Targeted Therapy:**   1. For NSCLC with Specific Genetic Mutations:    1. Drugs that target specific genetic mutations in cancer cells, such as EGFR, ALK, ROS1, BRAF, MET, and NTRK.    2. Often more effective than chemotherapy for patients with these mutations. 2. Oral Medications:   These medications are often taken orally.  **Immunotherapy:**   1. Stimulates the Immune System:    1. Drugs that help the body's immune system recognize and attack cancer cells.    2. Used for advanced NSCLC.    3. PD-1 and PD-L1 inhibitors are common forms of immunotherapy used. 2. Can be used alone or in combination with chemotherapy.   **Combined Modality Therapy:**  Combining Treatments:   1. Often, a combination of treatments is used, such as chemotherapy and radiation therapy, or surgery followed by chemotherapy. 2. The combination of immunotherapy and chemotherapy is also common.   **Palliative Care:**  Symptom Management:   1. Focuses on relieving symptoms and improving quality of life for patients with advanced lung cancer. 2. Can include pain management, breathing support, and emotional support.   **Clinical Trials:**  Investigational Treatments:  Participation in clinical trials may offer access to new and promising treatments.  **Key Considerations:**   1. Treatment decisions are made on a case-by-case basis, considering the individual patient's circumstances.   A multidisciplinary team of specialists, including pulmonologists, oncologists, surgeons, and radiation oncologists, is involved in treatment planning. |
| 1. **Is further treatment needed after surgery?** | Whether or not further treatment is needed after lung cancer surgery depends heavily on the stage of the cancer and the specific characteristics of the tumor. Here's a general overview:  **Factors Determining Need for Further Treatment:**   1. Stage of Cancer:    1. Early-stage lung cancer (especially stage I) may sometimes be treated successfully with surgery alone.    2. More advanced stages often require additional treatments. 2. Pathological Findings:    1. After surgery, the removed tissue is carefully examined. If cancer cells are found at the edges of the tissue (positive margins), it indicates that some cancer may have been left behind, necessitating further treatment.    2. The presence of cancer cells in the lymph nodes also signifies a higher risk of recurrence and usually leads to recommendations for additional therapy. 3. Type of Lung Cancer:   The type of lung cancer (NSCLC or SCLC) also influences post-surgical treatment decisions.   1. Patient's Overall Health:   A patient's overall health and ability to tolerate further treatments are also considered.  **Common Post-Surgical Treatments:**   1. Chemotherapy:   Often recommended after surgery to kill any remaining cancer cells and reduce the risk of recurrence. This is called adjuvant chemotherapy.   1. Radiation Therapy:   May be used to target any remaining cancer cells in the chest area, especially if positive margins were found during surgery.   1. Targeted Therapy:   For NSCLC with specific genetic mutations, targeted therapy may be recommended after surgery to further reduce the risk of recurrence.   1. Immunotherapy:   Immunotherapy can also be used after surgery, to help prevent the cancer from returning.   1. Follow-up Care:   Regardless of whether further treatment is needed, regular follow-up appointments with imaging studies and other tests are essential to monitor for any signs of recurrence.  **In summary:**   1. While surgery is a crucial part of lung cancer treatment, it's often just one component. 2. Further treatment after surgery is common, especially for more advanced stages of cancer.   The specific post-surgical treatment plan is tailored to each individual patient. |
| 1. **In what situations is targeted therapy for lung cancer applicable?** | Targeted therapy for lung cancer is applicable in specific situations primarily involving non-small cell lung cancer (NSCLC) and depends on the presence of particular genetic mutations or alterations within the tumor cells. Here's a breakdown:  **Key Situations for Targeted Therapy:**   1. **Presence of Specific Genetic Mutations:**     1. Targeted therapies are designed to attack cancer cells that have specific genetic abnormalities. Therefore, genetic testing of the tumor tissue is essential to identify these mutations.    2. Commonly targeted mutations include:       1. EGFR (Epidermal Growth Factor Receptor) mutations:          1. These mutations are more common in adenocarcinomas, particularly in non-smokers or light smokers.          2. EGFR inhibitors (e.g., gefitinib, erlotinib, osimertinib) are used to target these mutations.       2. ALK (Anaplastic Lymphoma Kinase) rearrangements:          1. These rearrangements are also more common in adenocarcinomas and in younger, non-smoking individuals.          2. ALK inhibitors (e.g., crizotinib, alectinib, brigatinib) are used to target these alterations.       3. ROS1 rearrangements:          1. Similar to ALK rearrangements, these are also more common in adenocarcinomas.          2. ROS1 inhibitors (e.g., crizotinib, entrectinib) are used.       4. BRAF V600E mutations:   BRAF inhibitors (e.g., dabrafenib, vemurafenib) are used, often in combination with MEK inhibitors (e.g. trametinib).   - - 1. MET exon 14 skipping mutations or MET amplifications:   MET inhibitors (e.g., capmatinib, tepotinib) are used.   - - 1. NTRK gene fusions:   NTRK inhibitors (e.g., entrectinib, larotrectinib) are used.   - - 1. RET rearrangements:   RET inhibitors (e.g., selpercatinib, pralsetinib) are used.   1. **Advanced or Metastatic NSCLC:**   Targeted therapies are most commonly used in patients with advanced or metastatic NSCLC, where the cancer has spread beyond the lungs.   1. **When Chemotherapy Is Not Optimal:**   In some cases, targeted therapy may be preferred over chemotherapy, especially in patients with specific genetic mutations and who may not tolerate chemotherapy well.   1. **Maintenance Therapy:**   In some cases, targeted therapies can be used as maintenance therapy after initial chemotherapy, to help prevent the cancer from returning.  **Important Considerations:**   1. Genetic Testing:   Comprehensive genetic testing is crucial to identify the presence of targetable mutations.   1. Ongoing Research:   The field of targeted therapy is rapidly evolving, with new targeted therapies and biomarkers being discovered.  In essence, targeted therapy is a personalized approach to lung cancer treatment, tailored to the specific genetic characteristics of each patient's tumor. |
| 1. **How effective is chemotherapy for lung cancer?** | The effectiveness of chemotherapy for lung cancer varies significantly depending on several factors, including the type of lung cancer (NSCLC or SCLC), the stage of the cancer, and the patient's overall health. Here's a general overview:  **Small Cell Lung Cancer (SCLC):**   1. SCLC is generally more responsive to chemotherapy than NSCLC. 2. Chemotherapy is often the primary treatment for SCLC, especially in the extensive stage (when the cancer has spread). 3. While initial response rates can be high, SCLC often develops resistance to chemotherapy, and recurrence is common. 4. Chemotherapy can significantly improve survival and quality of life for patients with SCLC.   **Non-Small Cell Lung Cancer (NSCLC):**   1. The effectiveness of chemotherapy for NSCLC varies depending on the stage of the cancer and the presence of specific genetic mutations. 2. In early-stage NSCLC, chemotherapy may be used after surgery (adjuvant chemotherapy) to reduce the risk of recurrence. 3. In advanced or metastatic NSCLC, chemotherapy can help to slow the progression of the disease, relieve symptoms, and improve survival. 4. However, in NSCLC, targeted therapy and immunotherapy have significantly changed the treatment landscape, and are often preferred when applicable. 5. Chemotherapy is still a very important part of treatment for NSCLC. Especially when targeted therapies, and immunotherapies are not an option.   **General Considerations:**   1. Chemotherapy can have significant side effects, which can impact a patient's quality of life. 2. The effectiveness of chemotherapy can be influenced by factors such as the patient's age, overall health, and the presence of other medical conditions. 3. Advances in lung cancer treatment, such as targeted therapy and immunotherapy, have provided additional options for patients, particularly those with NSCLC. 4. Chemotherapy is often used in combination with other treatments, such as radiation therapy or immunotherapy.   **Key Points:**   1. Chemotherapy remains an important treatment option for lung cancer, particularly SCLC and certain stages of NSCLC. 2. The effectiveness of chemotherapy varies, and treatment decisions are made on a case-by-case basis. 3. Ongoing research continues to improve the effectiveness of chemotherapy and reduce its side effects.   It is important to discuss treatment options and expectations with an oncologist to determine the most appropriate course of action. |
| 1. **In what situations is radiotherapy for lung cancer applicable?** | Radiotherapy plays a significant role in lung cancer treatment, and its applicability varies depending on the type and stage of the cancer, as well as the patient's overall health. Here's a breakdown of common situations where radiotherapy is used:  **Non-Small Cell Lung Cancer (NSCLC):**   1. Early-Stage NSCLC (Stage I and II):    1. Stereotactic body radiation therapy (SBRT) or stereotactic ablative radiotherapy (SABR) can be used as a primary treatment option for patients who are not suitable candidates for surgery due to medical comorbidities or patient preference.    2. Postoperative radiation therapy may be used after surgery if there are positive surgical margins or if there is a high risk of recurrence. 2. Locally Advanced NSCLC (Stage III):    1. Radiation therapy is often combined with chemotherapy (chemoradiation) for unresectable locally advanced NSCLC. This is considered a standard of care.    2. Radiation therapy may be used to shrink the tumor before surgery (neoadjuvant radiation therapy) in select cases. 3. Advanced or Metastatic NSCLC (Stage IV):    1. Palliative radiation therapy is used to relieve symptoms such as pain, bleeding, or airway obstruction caused by the tumor.    2. Brain metastases can be treated with whole-brain radiation therapy or stereotactic radiosurgery.    3. Bone metastases can be treated with radiation therapy to alleviate pain and prevent fractures.   **Small Cell Lung Cancer (SCLC):**   1. Limited-Stage SCLC:    1. Radiation therapy is typically combined with chemotherapy (chemoradiation) for limited-stage SCLC.    2. Prophylactic cranial irradiation (PCI) is often recommended after chemoradiation to reduce the risk of brain metastases. 2. Extensive-Stage SCLC:    1. Radiation therapy may be used for palliative purposes to relieve symptoms.    2. PCI may also be considered in select patients with extensive-stage SCLC who have responded well to initial chemotherapy.   **Other Situations:**   1. Superior Vena Cava Syndrome:   Radiation therapy can be used to relieve compression of the superior vena cava, which can cause swelling in the face, neck, and arms.   1. Endobronchial Obstruction:   Radiation therapy can be used to open up blocked airways.   1. Pain Management:   Radiotherapy can be used to treat pain from bone metastases, or nerve compression.  **Key Considerations:**   1. The specific radiation therapy technique and dose will depend on the individual patient's situation. 2. Radiation therapy can have side effects, which can vary depending on the area being treated.   A multidisciplinary team of specialists, including radiation oncologists, medical oncologists, and pulmonologists, will work together to develop a treatment plan. |
| 1. **What is immunotherapy?** | Immunotherapy is a type of cancer treatment that leverages the body's own immune system to fight cancer. Essentially, it helps your immune system recognize and attack cancer cells. Here's a breakdown:  **How it Works:**   1. The immune system naturally defends the body against foreign invaders, including abnormal cells like cancer cells. However, cancer cells can sometimes evade the immune system's detection. 2. Immunotherapy aims to boost or modify the immune system's response so that it can effectively target and destroy cancer cells.   **Types of Immunotherapy:**  There are several different types of immunotherapy, including:   1. Immune checkpoint inhibitors:   These drugs block "checkpoints" that normally prevent the immune system from becoming too active. By blocking these checkpoints, the immune system is allowed to mount a stronger attack against cancer cells.   1. T-cell transfer therapy:   This involves taking T-cells (a type of immune cell) from the patient's blood or tumor, modifying them in a lab to enhance their cancer-fighting ability, and then infusing them back into the patient.   1. Monoclonal antibodies:   These are laboratory-produced antibodies that are designed to target specific proteins on cancer cells. They can work in various ways, such as marking cancer cells for destruction by the immune system.   1. Cancer vaccines:   These vaccines stimulate the immune system to recognize and attack cancer cells. Unlike preventive vaccines, cancer vaccines are designed to treat existing cancer.   1. Cytokine therapy:   Cytokines are proteins that play a crucial role in immune system signaling. Cytokine therapy involves using cytokines to boost the immune response against cancer.  **Key Points:**   1. Immunotherapy has revolutionized cancer treatment, offering new hope for patients with various types of cancer. 2. It can be used alone or in combination with other cancer treatments, such as chemotherapy, radiation therapy, and targeted therapy. 3. While immunotherapy can be very effective, it can also cause side effects, as the immune system can sometimes attack healthy tissues. |
| **Topic: Prognosis** | |
| 1. **What is the frequency of follow-up after lung cancer surgery?** | Follow-up care after lung cancer surgery is crucial for monitoring for recurrence and managing any potential complications. While specific follow-up schedules can vary based on individual patient circumstances and the treating physician's recommendations, here's a general outline:  **General Follow-up Schedule:**   1. Initial Period (First 2 Years):    1. This is the period of highest risk for recurrence. Therefore, follow-up is typically more frequent.    2. Follow-up visits are often scheduled every 3 to 6 months.    3. These visits typically involve:       1. Physical examinations.       2. Imaging studies, such as CT scans.       3. Possibly blood tests. 2. Intermediate Period (Years 2-5):    1. If there are no signs of recurrence, the frequency of follow-up visits may decrease.    2. Visits may transition to every 6 months. 3. Long-Term Follow-up (After 5 Years):    1. After 5 years, follow-up visits may be reduced to once a year.    2. The focus shifts to monitoring for long-term complications and the development of new lung cancers.   **Key Components of Follow-up:**   1. Imaging:    1. CT scans are a primary tool for monitoring for recurrence.    2. Chest X-rays may also be used. 2. Physical Examinations:   Doctors will assess for any signs or symptoms of recurrence.   1. Symptom Monitoring:   Patients are encouraged to report any new or worsening symptoms, such as:   - - 1. Persistent cough.     2. Shortness of breath.     3. Chest pain.     4. Unexplained weight loss.  1. Smoking Cessation:   If the patient is a smoker, ongoing support and encouragement to quit are essential.  **Important Considerations:**   1. The specific follow-up plan should be tailored to the individual patient's risk factors and needs.   It's crucial to maintain open communication with the healthcare team and attend all scheduled follow-up appointments. |
| 1. **What should be done if a new nodule is found during follow-up?** | Finding a new nodule during follow-up after lung cancer treatment can be concerning, but it doesn't automatically mean a recurrence. The approach to managing a new nodule depends on several factors, and a systematic evaluation is crucial. Here's a general approach:  **Assessment of Risk Factors:**   1. Previous Lung Cancer History: The type and stage of the previous lung cancer are critical. 2. Smoking History: Continued smoking or a history of smoking increases the risk of new lung cancers. 3. Other Risk Factors: Exposure to radon, asbestos, or other carcinogens.   **Evaluation of the Nodule:**   1. Size and Characteristics:    1. The size, shape, density, and growth rate of the nodule are important.    2. Features such as spiculated margins, ground-glass opacities, and calcifications are noted. 2. Location:   The location of the nodule within the lung is considered.   1. Growth Rate:   Comparing the current CT scan with previous scans is essential to determine the nodule's growth rate. Rapid growth is more concerning.  **Imaging Studies:**   1. Repeat CT Scan:   A follow-up CT scan may be performed in a shorter interval to assess the nodule's growth.   1. PET-CT Scan:   A PET-CT scan can help determine the metabolic activity of the nodule, helping to differentiate between benign and malignant lesions.   1. Bronchoscopy:   If the nodule is accessible via bronchoscopy, a biopsy may be performed.   1. CT-Guided Needle Biopsy:   For peripheral nodules, a CT-guided needle biopsy may be performed to obtain a tissue sample.   1. Surgical Biopsy:   In some cases, a surgical biopsy (thoracoscopy or thoracotomy) may be necessary to obtain a definitive diagnosis.  **Clinical Decision-Making:**   1. Multidisciplinary Team:   A multidisciplinary team, including pulmonologists, oncologists, radiologists, and surgeons, should be involved in the evaluation and management of the nodule.   1. Risk Stratification:   The risk of malignancy is assessed based on the nodule's characteristics and the patient's risk factors.   1. Management Options:    1. Observation: If the nodule is small, stable, and has low suspicion for malignancy, observation with serial CT scans may be appropriate.    2. Biopsy: If the nodule is suspicious for malignancy, a biopsy is recommended.    3. Surgical Resection: If the nodule is highly suspicious for malignancy or if a biopsy confirms lung cancer, surgical resection may be recommended.   **Important Considerations:**   1. Early Detection:   Early detection of recurrence or new lung cancers is crucial for improving outcomes.   1. Patient Anxiety:   Finding a new nodule can cause significant anxiety. Open communication and support are essential.   1. It is very important that any new nodule found should be evaluated by the patients medical team, and that no assumptions should be made about the nature of the nodule without proper medical evaluation.   In summary, the management of a new nodule after lung cancer treatment requires a careful and systematic approach. The goal is to determine the nature of the nodule and to provide appropriate treatment if necessary. |
| 1. **How to determine whether lung cancer recurs during follow-up?** | Detecting lung cancer recurrence during follow-up involves a combination of careful monitoring, imaging, and sometimes invasive procedures. Here's a breakdown of how it's typically done:  **Regular Follow-up Appointments:**  Consistent appointments with an oncologist are essential. These visits allow for:   1. Physical examinations. 2. Review of any new or persistent symptoms. 3. Discussion of the patient's overall well-being.   **Imaging Studies:**   1. CT Scans:    1. These are the primary imaging tool for detecting lung cancer recurrence.    2. They provide detailed images of the lungs and surrounding structures, allowing for the detection of new nodules, masses, or changes in existing abnormalities. 2. PET-CT Scans:    1. These scans can help identify metabolically active areas, which may indicate cancer recurrence.    2. They are particularly useful for distinguishing between scar tissue and recurrent tumors. 3. Other Imaging:   Depending on the patient's symptoms and risk factors, other imaging studies, such as MRI or bone scans, may be used to detect distant metastases.  **Symptom Monitoring:**   1. Patients are advised to report any new or worsening symptoms, such as:    1. Persistent cough.    2. Coughing up blood.    3. Shortness of breath.    4. Chest pain.    5. Unexplained weight loss.    6. Fatigue.    7. Any new pain. 2. These symptoms may indicate local, regional, or distant recurrence.   **Biopsy:**   1. If imaging studies or symptoms suggest recurrence, a biopsy is often necessary to confirm the diagnosis. 2. Biopsy methods may include:    1. Bronchoscopy.    2. CT-guided needle biopsy.    3. Surgical biopsy.   **Tumor Markers:**  In some cases, blood tests for tumor markers may be used to monitor for recurrence. However, these are not always reliable for lung cancer.  **Key Considerations:**   1. Early Detection:   Early detection of recurrence is crucial for improving treatment outcomes.   1. Individualized Approach:   The frequency and type of follow-up tests will vary depending on the individual patient's risk factors and the stage of the initial cancer.   1. Multidisciplinary Care:   A team of specialists, including oncologists, radiologists, and pulmonologists, is involved in monitoring for recurrence.  By combining these methods, healthcare professionals can effectively monitor for lung cancer recurrence and provide appropriate treatment if necessary. |
| 1. **How to manage lifestyle after lung cancer surgery?** | Managing your lifestyle after lung cancer surgery is crucial for recovery and overall well-being. Here's a breakdown of key areas to focus on:  **Pulmonary Rehabilitation and Exercise:**   1. Breathing Exercises:    1. These are essential to improve lung function and prevent complications like pneumonia.    2. Techniques like deep breathing and coughing exercises help clear secretions and expand the lungs. 2. Gradual Exercise:    1. Start with gentle activities and gradually increase intensity as tolerated.    2. Walking is an excellent way to improve cardiovascular fitness.    3. Pulmonary rehabilitation programs can provide structured exercise and guidance. 3. Maintain a healthy weight:   Being overweight or underweight can cause additional stress on the lungs.  **Nutrition:**   1. Balanced Diet:    1. Focus on a diet rich in fruits, vegetables, lean proteins, and whole grains.    2. Adequate protein intake is essential for tissue healing. 2. Hydration:   Drink plenty of fluids to stay hydrated and help thin mucus.   1. Small, Frequent Meals:   This can be easier to tolerate than large meals, especially if you experience shortness of breath.   1. Nutritional Supplements:   Consult with your doctor or a registered dietitian to determine if supplements are needed.  **Smoking Cessation:**  Absolutely Essential:   1. If you are still smoking, quitting is the most important step you can take to improve your health. 2. Smoking increases the risk of recurrence and complications. 3. Seek support from smoking cessation programs or healthcare professionals.   **Emotional and Mental Health:**   1. Managing Stress and Anxiety:    1. Lung cancer and surgery can be emotionally challenging.    2. Seek support from family, friends, support groups, or mental health professionals. 2. Getting Adequate Sleep:   Rest is essential for recovery.   1. Mindfulness and Relaxation Techniques:   These can help manage stress and improve overall well-being.  **Pain Management:**   1. Follow Doctor's Instructions:    1. Take pain medications as prescribed.    2. Communicate with your doctor about any pain you experience. 2. Alternative Therapies:   Techniques like massage, acupuncture, or heat therapy may help manage pain.  **Follow-up Care:**   1. Attend All Appointments:   Regular follow-up visits are crucial for monitoring your recovery and detecting any signs of recurrence.   1. Report Any Symptoms:   Don't hesitate to contact your healthcare team if you experience any new or worsening symptoms.  **Infection Prevention:**   1. Hand Hygiene:   Wash your hands frequently to prevent infections.   1. Avoid Sick People:   Limit contact with people who have colds or other infections.   1. Vaccinations:   Get recommended vaccinations, such as the flu and pneumonia vaccines.  **Support Systems:**   1. Family and Friends:   Lean on your support network for emotional and practical assistance.   1. Support Groups:   Connecting with other lung cancer survivors can provide valuable support and understanding.  By adopting these lifestyle changes, you can optimize your recovery and improve your quality of life after lung cancer surgery. |
| 1. **Whether the focus of follow-up for long-term survivors (e.g., more than 5 years after surgery) needs to be adjusted?** | Yes, the focus of follow-up for long-term lung cancer survivors (e.g., more than 5 years after surgery) often needs to be adjusted. While the risk of recurrence significantly decreases after this period, other important considerations come into play. Here's a breakdown of how the focus shifts:  **Shifting Focus:**   1. Reduced Emphasis on Recurrence Monitoring:    1. While surveillance for recurrence remains important, the intensity of imaging studies (like frequent CT scans) may be reduced.    2. The interval between follow-up visits may be extended. 2. Increased Emphasis on Long-Term Effects:    1. Second Primary Lung Cancers: Long-term survivors, especially those with a smoking history, remain at risk for developing new lung cancers. Therefore, continued vigilance for new pulmonary nodules or symptoms is essential.    2. Comorbidities: Many lung cancer survivors have other health conditions (e.g., cardiovascular disease, COPD) that require ongoing management.    3. Late Effects of Treatment:       1. Radiation therapy can cause long-term lung damage (e.g., pulmonary fibrosis).       2. Chemotherapy can have long-lasting effects on the heart, nerves, and other organs.       3. Surgical complications can arise later.    4. Quality of Life:       1. Addressing physical and emotional well-being becomes paramount.       2. This includes managing chronic pain, fatigue, and psychological distress.       3. Pulmonary rehabilitation and exercise programs remain important.    5. Smoking Cessation Support:   Reinforcing smoking cessation is crucial, as the risk of second primary lung cancers and other smoking-related diseases remains elevated.   - 1. Psychosocial Support:   Long term cancer survivors can still have psychological problems such as PTSD, anxiety, and depression. These should be addressed.   - 1. Nutritional counseling:   Helping patients maintain a healthy diet is a long term need.  **Specific Adjustments:**   1. Imaging:    1. The frequency of CT scans may be reduced or transitioned to lower-dose CT scans.    2. The focus shifts to detecting new lung nodules rather than monitoring for subtle changes in previous surgical sites. 2. Pulmonary Function Tests (PFTs):   Regular PFTs may be continued to monitor lung function, especially in patients with pre-existing lung disease or those who received radiation therapy.   1. Cardiovascular Monitoring:   Patients who received certain chemotherapy drugs or radiation therapy to the chest may require ongoing cardiovascular monitoring.   1. Symptom Management:   The focus shifts to managing chronic symptoms and improving quality of life.  **Key Takeaway:**  The long-term follow-up of lung cancer survivors requires a holistic approach that addresses not only the risk of recurrence but also the long-term effects of treatment and the patient's overall well-being. |
| 1. **How to manage long-term side effects after treatment, such as lung function damage, radiation pneumonitis, etc.** | Managing long-term side effects after lung cancer treatment requires a multidisciplinary approach, focusing on symptom control, rehabilitation, and preventative measures. Here's how to address common long-term effects:  **Lung Function Damage (Pulmonary Fibrosis, Chronic Obstructive Pulmonary Disease - COPD):**   1. Pulmonary Rehabilitation:   This is a cornerstone of management. It includes:   - - 1. Breathing exercises (diaphragmatic breathing, pursed-lip breathing).     2. Exercise training to improve endurance and reduce shortness of breath.     3. Education on energy conservation techniques.  1. Oxygen Therapy:   Supplemental oxygen may be needed for patients with persistent hypoxemia (low blood oxygen levels).   1. Medications:    1. Bronchodilators (e.g., albuterol, tiotropium) can help open up airways.    2. Inhaled corticosteroids may be used to reduce inflammation.    3. For Pulmonary fibrosis, medications such as pirfenidone and nintedanib can slow down the progression of the disease. 2. Lifestyle Modifications:    1. Smoking cessation is crucial.    2. Maintaining a healthy weight.    3. Avoiding respiratory irritants (e.g., dust, smoke).    4. Vaccinations against influenza and pneumonia.   **Radiation Pneumonitis/Fibrosis:**   1. Corticosteroids:   These are often used to reduce inflammation in acute radiation pneumonitis.   1. Oxygen Therapy:   Supplemental oxygen may be required.   1. Pulmonary Rehabilitation:   Similar to lung function damage, pulmonary rehabilitation can help improve breathing and exercise tolerance.   1. Monitoring:   Regular pulmonary function tests and imaging studies are important for monitoring lung function.   1. Medications:   As stated above, medications such as pirfenidone and nintedanib can slow down the progression of pulmonary Fibrosis.  **Chronic Pain:**   1. Pain Medications:   Nonsteroidal anti-inflammatory drugs (NSAIDs), opioids, or neuropathic pain medications may be used.   1. Physical Therapy:   Physical therapy can help improve mobility and reduce pain.   1. Alternative Therapies:   Acupuncture, massage, and relaxation techniques may provide pain relief.   1. Nerve Blocks:   In some cases, nerve blocks can be used to manage localized pain.  **Fatigue:**   1. Exercise:   Gradual exercise can help improve energy levels.   1. Sleep Hygiene:   Establish a regular sleep schedule and create a relaxing bedtime routine.   1. Nutritional Counseling:   A balanced diet can help improve energy levels.   1. Psychological Support:   Counseling or support groups can help manage fatigue-related distress.  **Cardiovascular Issues:**   1. Monitoring:   Regular cardiovascular monitoring is essential, especially for patients who received certain chemotherapy drugs or radiation therapy to the chest.   1. Medications:   Medications may be needed to manage hypertension, heart failure, or other cardiovascular conditions.   1. Lifestyle Modifications:   A heart-healthy diet, regular exercise, and smoking cessation are crucial.  **Psychological and Emotional Support:**   1. Counseling:   Individual or group counseling can help manage anxiety, depression, and other psychological issues.   1. Support Groups:   Connecting with other lung cancer survivors can provide valuable support and understanding.   1. Mindfulness and Relaxation Techniques:   These can help manage stress and improve overall well-being.  **Neuropathy:**   1. Medications:   Medications such as gabapentin or pregabalin can help manage neuropathic pain.   1. Physical Therapy:   Physical therapy can help improve balance and coordination.  **Key Principles:**   1. Multidisciplinary Approach:   A team of specialists, including pulmonologists, oncologists, pain specialists, physical therapists, and psychologists, should be involved in managing long-term side effects.   1. Individualized Treatment:   Treatment plans should be tailored to the individual patient's needs and symptoms.   1. Regular Monitoring:   Regular follow-up appointments are essential for monitoring symptoms and adjusting treatment as needed.   1. Patient Education:   Patients should be educated about their long-term side effects and how to manage them. |
| **Topic: Case** | |
| 1. **A 68-year-old male patient, with a 40-year history of smoking, has been experiencing cough and blood-tinged sputum for 3 months. Chest X-ray showed an irregular mass in the right upper lung with a rough edge; CT scan suggested that the mass invaded the pleura and mediastinal lymph nodes were enlarged. Lung cancer is initially considered, and bronchoscopy and biopsy are recommended to confirm the pathology. What further tests does the patient need to confirm the diagnosis?** | This clinical scenario strongly suggests lung cancer. To confirm the diagnosis and determine the extent of the disease, the following further tests are essential:   1. **Bronchoscopy with Biopsy and Cytology:**    1. As already recommended, this is crucial for obtaining tissue samples for pathological confirmation.    2. Biopsy: A direct tissue sample from the mass.    3. Bronchial washings and brushings: Collecting cells from the airways for cytological examination.    4. This will allow for:       1. Histological diagnosis (e.g., NSCLC subtype, SCLC).       2. Immunohistochemistry (IHC) to further characterize the tumor.       3. Molecular testing (genetic testing) to identify targetable mutations. 2. **Pathological Evaluation:**    1. Histopathology: Microscopic examination of the biopsied tissue to confirm the presence of cancer cells and determine the specific type of lung cancer.    2. Immunohistochemistry (IHC): To further characterize the tumor and identify specific markers.    3. Molecular Testing (Genetic Testing):       1. Essential for identifying targetable mutations, especially in adenocarcinoma.       2. Tests for EGFR, ALK, ROS1, BRAF, MET, PD-L1, and other relevant markers.       3. This guides targeted therapy and immunotherapy decisions. 3. **Imaging for Staging:**    1. PET-CT Scan:       1. Crucial for assessing the metabolic activity of the mass and detecting potential metastases in distant organs.       2. Helps to determine the extent of lymph node involvement.       3. Provides a more accurate staging than CT alone.    2. Brain MRI:   To evaluate for brain metastases, especially given the presence of mediastinal lymph node enlargement, which increases the likelihood of distant spread.   - 1. Bone Scan or PET Bone Scan:   If the patient has bone pain, or if the PET-CT scan suggests bone involvement, a bone scan or PET bone scan should be performed to evaluate for bone metastases.   - 1. Endobronchial Ultrasound (EBUS) with Needle Aspiration:   To get a tissue sample from the enlarged mediastinal lymph nodes seen on the CT scan. This is a less invasive way to stage the lymph nodes than a mediastinoscopy.   1. **Pulmonary Function Tests (PFTs):**    1. To assess lung function and determine the patient's respiratory reserve, especially if surgery is considered.    2. Essential for pre-operative evaluation. 2. **Complete Blood Count (CBC) and Comprehensive Metabolic Panel (CMP):**    1. To assess the patient's overall health and identify any abnormalities.    2. Essential for pre-treatment evaluation. 3. **Arterial Blood Gas (ABG) Analysis:**   To assess blood oxygen and carbon dioxide levels.   1. **Performance Status Assessment:**   To evaluate the patient's overall functional status (e.g., ECOG or Karnofsky score).  **Rationale:**   1. The 40-year smoking history and clinical presentation are highly suggestive of lung cancer. 2. The chest X-ray and CT scan findings indicate a potentially advanced stage of the disease. 3. Pathological confirmation is essential for definitive diagnosis and treatment planning. 4. Staging is crucial for determining the extent of the disease and guiding treatment decisions.   Because the CT scan showed mediastinal lymph node involvement, it is very important to get a tissue sample from those lymph nodes. EBUS is a good less invasive way of accomplishing this. |
| 1. **A 55-year-old female patient, diagnosed with advanced non-small cell lung cancer (NSCLC), with EGFR gene mutation positive. She has received gefitinib targeted therapy, but recently developed drug resistance and tumor progression. How to adjust the next treatment plan?** | This scenario describes a common challenge in treating advanced NSCLC with EGFR mutations: the development of resistance to first-generation EGFR tyrosine kinase inhibitors (TKIs) like gefitinib. Here's how to adjust the treatment plan:   1. **Confirm EGFR T790M Mutation:**   Repeat Biopsy and Genetic Testing:   - - 1. A repeat biopsy of the tumor is crucial to determine the mechanism of resistance.     2. Specifically, test for the EGFR T790M mutation, which is the most common cause of acquired resistance to gefitinib.     3. Liquid biopsy (ctDNA analysis) can also be considered as a less invasive alternative.  1. **If EGFR T790M Positive:**   Osimertinib (Tagrisso):   - - 1. Osimertinib is a third-generation EGFR TKI specifically designed to target both EGFR activating mutations and the T790M resistance mutation.     2. It is the standard of care for patients with T790M-positive NSCLC who have progressed on first-generation EGFR TKIs.     3. It typically has better efficacy and tolerability than chemotherapy in this setting.  1. **If EGFR T790M Negative (or if Osimertinib Fails):**    1. Consider Other Resistance Mechanisms:       1. Other resistance mechanisms can occur, such as MET amplification, HER2 amplification, BRAF mutations, or PIK3CA mutations.       2. Next-generation sequencing (NGS) of the tumor tissue or liquid biopsy can help identify these alternative resistance mechanisms.    2. Chemotherapy:       1. If no other targetable mutations are identified, chemotherapy is the next line of treatment.       2. Platinum-based doublet chemotherapy (e.g., platinum + pemetrexed) is commonly used.    3. Immunotherapy:       1. Immunotherapy, such as PD-1 or PD-L1 inhibitors, may be considered in select patients, especially if the tumor has high PD-L1 expression.       2. However, immunotherapy has generally shown less efficacy in EGFR-mutated NSCLC compared to chemotherapy.    4. Clinical Trials:       1. Participation in clinical trials investigating new targeted therapies or combination therapies should be considered.       2. This may offer access to potentially more effective treatments.    5. MET Inhibitors, BRAF Inhibitors, HER2 Inhibitors:   If other resistance mechanisms are found, then targeted therapies against those mechanisms should be initiated.   1. **Palliative Care:**    1. Symptom Management:   Regardless of the treatment approach, palliative care should be integrated to manage symptoms such as pain, shortness of breath, and fatigue.   - 1. Quality of Life:   Maintaining the patient's quality of life is a primary goal.  **Key Steps:**   1. Repeat Biopsy/Liquid Biopsy: Confirm the presence or absence of the T790M mutation. 2. Osimertinib: If T790M positive, switch to osimertinib. 3. NGS: If T790M negative, identify other resistance mechanisms. 4. Chemotherapy/Immunotherapy/Clinical Trials: If no other targetable mutations are found, consider chemotherapy, immunotherapy, or clinical trials.   Palliative Care: Integrate palliative care for symptom management. |
| 1. **A 70-year-old male patient, on the 3rd day after lung cancer surgery, developed pleural effusion and poor drainage. Body temperature was 37.8°C, heart rate was 90 beats/min, respiratory rate was 22 breaths/min, and the drainage tube was blocked. The patient complained of chest tightness. How to deal with the blocked drainage tube?** | This patient is experiencing a common post-operative complication following lung cancer surgery: pleural effusion with a blocked drainage tube, leading to chest tightness and potential infection. Here's a step-by-step approach to managing this situation:   1. **Immediate Assessment and Stabilization:**    1. Respiratory Status:       1. Assess the patient's oxygen saturation and work of breathing.       2. Administer supplemental oxygen if needed.       3. Auscultate lung sounds to evaluate the extent of the pleural effusion and any potential atelectasis.    2. Cardiovascular Status:       1. Monitor heart rate and blood pressure.       2. Assess for signs of hemodynamic instability.    3. Pain Assessment:       1. Evaluate the patient's level of chest tightness and pain.       2. Administer pain medication as needed. 2. **Addressing the Blocked Drainage Tube:** 3. Gentle Irrigation:    - 1. Attempt to gently irrigate the drainage tube with sterile saline solution to dislodge any clots or debris.      2. Use aseptic technique to prevent infection. 4. Tube Repositioning:    - 1. If irrigation is unsuccessful, gently manipulate the drainage tube to ensure it is properly positioned within the pleural space.      2. Sometimes the tube can become kinked or displaced. 5. Tube Replacement:    - 1. If irrigation and repositioning fail to clear the blockage, the drainage tube may need to be replaced.      2. This should be performed by a qualified healthcare professional. 6. Imaging:   A chest X-ray, or CT scan, may be needed to confirm the amount of pleural fluid, and the placement of the tube.   1. **Management of Pleural Effusion:**    1. Thoracentesis:       1. If the pleural effusion is large or causing significant respiratory distress, a thoracentesis may be performed to drain the fluid.       2. This can provide immediate relief of chest tightness and improve lung function.    2. Pleurodesis:   If the pleural effusion is recurrent, pleurodesis (a procedure to obliterate the pleural space) may be considered.   - 1. Treat the cause:   It is important to determine why the pleural effusion is so large. Is it because of a leak, or an infection?   1. **Addressing Potential Infection:** 2. Blood Cultures:   Obtain blood cultures to evaluate for bacteremia.   1. Sputum Cultures:   If the patient is producing sputum, obtain a sample for culture.   1. Pleural Fluid Analysis:   If pleural fluid is drained, send it for analysis (cell count, protein, glucose, cultures) to evaluate for infection.   1. Antibiotic Therapy:   If infection is suspected or confirmed, initiate appropriate antibiotic therapy.   1. **Supportive Care:**    1. Oxygen Therapy:   Continue supplemental oxygen as needed to maintain adequate oxygen saturation.   - 1. Pain Management:   Provide adequate pain relief to improve patient comfort and facilitate deep breathing.   - 1. Chest Physiotherapy:   Chest physiotherapy can help clear secretions and improve lung function.   - 1. Close Monitoring:   Closely monitor the patient's respiratory status, vital signs, and drainage output.  **Key Considerations:**   1. Aseptic Technique:   Strict aseptic technique is essential during any procedure involving the drainage tube to prevent infection.   1. Experienced Personnel:   Procedures such as tube replacement and thoracentesis should be performed by experienced healthcare professionals.   1. Prompt Intervention:   Prompt intervention is crucial to prevent complications such as respiratory distress and empyema (infection in the pleural space).   1. Team approach: 2. A team approach including surgeons, pulmonologists, and nurses, is very important. |
| 1. **A 48-year-old female patient, newly diagnosed with advanced lung cancer, is feeling low and refusing treatment. The patient has obvious anxiety and depression, worrying about the side effects of treatment and family burden. How to help the patient rebuild treatment confidence** | This patient is facing a significant emotional and psychological burden alongside her new lung cancer diagnosis. Here's a comprehensive approach to help her rebuild treatment confidence:   1. **Establish Trust and Empathy:**    1. Active Listening:       1. Listen attentively to her concerns, fears, and anxieties without judgment.       2. Acknowledge and validate her feelings.    2. Empathy:       1. Show genuine empathy and understanding of her situation.       2. Let her know that her feelings are normal and valid.    3. Build Rapport:   Create a safe and supportive environment where she feels comfortable expressing her emotions.   1. **Address Specific Concerns:**    1. Side Effects of Treatment:       1. Provide clear and accurate information about the potential side effects of treatment.       2. Explain that side effects can often be managed effectively with medications and supportive care.       3. Discuss personalized treatment plans that minimize side effects.       4. Provide statistics on modern treatment success, and the improvements that have been made to reduce side effects.    2. Family Burden:       1. Discuss her concerns about the financial and emotional burden on her family.       2. Explore available resources, such as financial assistance programs, support groups, and home healthcare services.       3. Encourage open communication with family members to address concerns and develop a support system.       4. Social workers, and case managers are very helpful in this situation.    3. Anxiety and Depression:       1. Recognize and address her anxiety and depression.       2. Refer her to a mental health professional (psychiatrist or psychologist) for evaluation and treatment.       3. Consider medication, therapy, or a combination of both. 2. **Provide Education and Information:**    1. Lung Cancer Information:       1. Provide comprehensive information about her specific type of lung cancer, stage, and treatment options.       2. Use clear and understandable language, avoiding medical jargon.       3. Provide access to reliable resources, such as reputable websites and patient advocacy groups.    2. Treatment Options:       1. Explain the benefits and risks of each treatment option.       2. Discuss the goals of treatment, such as extending survival, improving quality of life, or managing symptoms.       3. Emphasize the importance of early intervention and adherence to treatment.    3. Personalized Treatment Plan:       1. Develop a personalized treatment plan that considers her individual needs, preferences, and goals.       2. Involve her in the decision-making process. 3. **Build a Support System:**    1. Family and Friends:       1. Encourage her to lean on her support network for emotional and practical assistance.       2. Facilitate communication between her and her family members.    2. Support Groups:       1. Connect her with lung cancer support groups, either in person or online.       2. Support groups provide a sense of community and allow her to share experiences with others facing similar challenges.    3. Healthcare Team:       1. Emphasize the importance of open communication with her healthcare team.       2. Assure her that her healthcare team is there to support her throughout her journey. 4. **Foster Hope and Empowerment:**    1. Positive Outlook:       1. Maintain a positive and hopeful outlook while being realistic about the challenges ahead.       2. Share stories of lung cancer survivors who have achieved positive outcomes.    2. Empowerment:       1. Empower her to take an active role in her care.       2. Encourage her to ask questions, express her concerns, and make informed decisions.   **Key Strategies:**   1. Multidisciplinary Team:   Involve a multidisciplinary team of specialists, including oncologists, nurses, social workers, and mental health professionals.   1. Patient-Centered Approach:   Prioritize the patient's needs, preferences, and goals.   1. Ongoing Support:   Provide ongoing support and reassurance throughout her treatment journey.   1. Palliative Care:   Introduce palliative care early, so that the patient understands that this is a part of good cancer care, and not a sign that treatment is failing.  By addressing her concerns, providing education and support, and fostering hope, you can help this patient rebuild her treatment confidence and improve her overall well-being. |
| 1. **A 65-year-old male patient, diagnosed with locally advanced non-small cell lung cancer, is not suitable for surgical resection. He has completed concurrent chemoradiotherapy, but developed radiation pneumonitis and shortness of breath. How to relieve the symptoms of radiation pneumonitis?** | This patient is experiencing a common complication of concurrent chemoradiotherapy for locally advanced NSCLC: radiation pneumonitis. Here's how to manage the symptoms and provide relief:   1. **Assessment and Monitoring:**    1. Respiratory Assessment:       1. Assess the severity of shortness of breath, cough, and any other respiratory symptoms.       2. Monitor oxygen saturation, respiratory rate, and lung sounds.    2. Imaging:   A chest CT scan can help evaluate the extent of radiation pneumonitis and rule out other complications (e.g., infection, pleural effusion).   - 1. Pulmonary Function Tests (PFTs):   PFTs can assess lung function and monitor the progression of radiation pneumonitis.   - 1. Blood Tests:   A complete blood count (CBC) and inflammatory markers can help evaluate for infection.   1. **Treatment of Radiation Pneumonitis:**    1. Corticosteroids:       1. Corticosteroids (e.g., prednisone) are the mainstay of treatment for radiation pneumonitis.       2. They help reduce inflammation in the lungs.       3. The dosage and duration of corticosteroid therapy will depend on the severity of the pneumonitis.       4. A tapering dose is usually used to minimize side effects.    2. Oxygen Therapy:       1. Supplemental oxygen should be provided to maintain adequate oxygen saturation.       2. The level of oxygen support will depend on the patient's oxygen requirements.    3. Bronchodilators:   Bronchodilators (e.g., albuterol) can help open up airways and relieve shortness of breath.   - 1. Cough Suppressants:   Cough suppressants may be used to relieve persistent cough.   - 1. Pulmonary Rehabilitation:      1. Pulmonary rehabilitation can help improve lung function and exercise tolerance.      2. It includes breathing exercises, exercise training, and education on energy conservation.  1. **Supportive Care:**    1. Hydration:   Adequate hydration is essential to help thin secretions.   - 1. Nutritional Support:   Ensure the patient is receiving adequate nutrition to support recovery.   - 1. Pain Management:   Provide pain medication as needed to manage chest pain or discomfort.   - 1. Rest:   Adequate rest is crucial for recovery.   1. **Monitoring for Complications:**    1. Infection:       1. Radiation pneumonitis can increase the risk of infection.       2. Monitor for signs of infection (e.g., fever, increased cough, purulent sputum).       3. Promptly treat any infections with appropriate antibiotics.    2. Pulmonary Fibrosis:       1. Radiation pneumonitis can lead to pulmonary fibrosis (scarring of the lungs).       2. Monitor for signs of progressive lung function decline.       3. Medications such as pirfenidone and nintedanib can slow down the progression of pulmonary fibrosis. 2. **Follow-up Care:**    1. Regular Follow-up:   Regular follow-up appointments are essential to monitor lung function and manage any long-term complications.   - 1. Long term oxygen therapy:   Some patients will require long term oxygen therapy.  **Key Considerations:**   1. Early Intervention:   Early recognition and treatment of radiation pneumonitis are crucial to prevent complications.   1. Individualized Treatment:   Treatment plans should be tailored to the individual patient's needs and symptoms.   1. Multidisciplinary Approach:   A multidisciplinary team, including pulmonologists, oncologists, and respiratory therapists, should be involved in the patient's care.   1. Patient Education:   Educate the patient about radiation pneumonitis, its symptoms, and how to manage them. |

**Supplementary Table 2c:** ChatGpt 4o Responses to Lung Cancer Questions

| **Question** | **ChatGpt 4o Response** |
| --- | --- |
| **Topic: Overview** | |
| 1. **What is lung cancer?** | Lung cancer is a type of cancer that begins in the lungs, characterized by uncontrolled cell growth in lung tissues. This abnormal growth can interfere with normal lung function and has the potential to spread to other parts of the body.  **Types of Lung Cancer**  The two primary types of lung cancer are:   1. **Non-Small Cell Lung Cancer (NSCLC):** Accounting for approximately 80% to 85% of lung cancers, NSCLC includes subtypes such as adenocarcinoma, squamous cell carcinoma, and large cell carcinoma. These subtypes originate from different types of lung cells but are often grouped together due to similarities in treatment and prognosis. 2. **Small Cell Lung Cancer (SCLC):** Representing about 10% to 15% of lung cancers, SCLC tends to grow and spread more rapidly than NSCLC. It is often associated with a history of smoking and may have already spread to other parts of the body at the time of diagnosis.   **Causes and Risk Factors**  Several factors can increase the risk of developing lung cancer:   1. Smoking: The leading cause of lung cancer, with tobacco smoke containing numerous carcinogens that damage lung cells. Both active smoking and exposure to secondhand smoke significantly elevate the risk. 2. Exposure to Radon Gas: A naturally occurring radioactive gas that can accumulate in homes and buildings, increasing lung cancer risk. 3. Asbestos and Other Carcinogens: Occupational exposure to substances like asbestos, arsenic, and certain chemicals can heighten the risk. 4. Air Pollution: Long-term exposure to high levels of air pollution has been linked to an increased risk of lung cancer. 5. Genetic Factors: A family history of lung cancer can predispose individuals to the disease, suggesting a genetic component.   **Symptoms**  Lung cancer symptoms often do not appear until the disease is advanced. Common symptoms include:   1. A persistent cough that worsens over time 2. Chest pain 3. Shortness of breath 4. Coughing up blood 5. Unexplained weight loss 6. Fatigue   If you experience any of these symptoms, especially if you have risk factors for lung cancer, it is important to consult a healthcare provider.  **Diagnosis and Treatment**  Diagnosis typically involves imaging tests such as chest X-rays or CT scans, followed by a biopsy to confirm the presence of cancer cells. Treatment options depend on the type and stage of lung cancer and may include surgery, radiation therapy, chemotherapy, targeted therapy, and immunotherapy. Early detection significantly improves the effectiveness of treatment and survival rates.  For more detailed information, resources like the American Cancer Society and the Centers for Disease Control and Prevention offer comprehensive overviews of lung cancer. |
| 1. **What are the clinical features of lung cancer?** | Lung cancer often remains asymptomatic in its early stages, with clinical features typically emerging as the disease advances. Recognizing these symptoms is crucial for early detection and treatment.  **Respiratory Symptoms:**   1. Persistent Cough: A new cough that doesn't go away or a chronic cough that worsens over time. 2. Hemoptysis: Coughing up blood, even in small amounts. 3. Shortness of Breath: Difficulty breathing or wheezing. 4. Chest Pain: Discomfort or pain in the chest that may worsen with deep breathing, coughing, or laughing.   **Systemic Symptoms:**   1. Unexplained Weight Loss: Losing weight without trying. 2. Fatigue: Feeling unusually tired or weak. 3. Loss of Appetite: Decreased desire to eat.   **Other Possible Symptoms:**   1. Hoarseness: Changes in voice, such as becoming raspy. 2. Frequent Infections: Recurring respiratory infections like bronchitis or pneumonia. 3. Swelling: Swelling in the face, neck, arms, or upper chest due to superior vena cava syndrome.   It's important to note that these symptoms can also be associated with other health conditions. However, if you experience any of these symptoms persistently, especially if you have risk factors for lung cancer, it's essential to consult a healthcare provider for a thorough evaluation.  Early detection of lung cancer can significantly improve treatment outcomes and survival rates. Therefore, awareness of these clinical features is vital. |
| 1. **Is lung cancer hereditary?** | Yes, lung cancer can have a hereditary component, meaning that genetic factors can influence an individual's risk of developing the disease. Here's how heredity plays a role:  **Family History and Genetic Predisposition**   1. **Increased Risk with Family History:** Individuals with a close relative (such as a parent or sibling) who has had lung cancer are at a higher risk of developing the disease themselves. This elevated risk persists even after accounting for shared environmental factors like smoking. 2. **Inherited Genetic Mutations:** Certain inherited genetic mutations can increase susceptibility to lung cancer. For example, mutations in the CHEK2 gene have been linked to a higher risk of developing various cancers, including lung cancer.     **Genetic Variants and Susceptibility**  **Chromosomal Regions Associated with Risk:** Specific regions on chromosomes, such as 15q25 and 5p15, have been associated with an increased risk of lung cancer. These loci contain genes that, when mutated, may contribute to cancer development.    **Gene-Environment Interactions**  **Combined Effects:** Genetic predisposition can interact with environmental factors, such as smoking or exposure to carcinogens, to further elevate lung cancer risk. For instance, individuals with certain genetic backgrounds may be more susceptible to the harmful effects of tobacco smoke.  **Sporadic vs. Hereditary Cases**  While hereditary factors do play a role, it's important to note that the majority of lung cancer cases are sporadic, resulting from environmental exposures and lifestyle choices rather than inherited genetic mutations.  **Implications for Screening and Prevention**  Understanding one's family history and genetic predisposition can inform personalized screening strategies and preventive measures. Individuals with a family history of lung cancer or known genetic mutations associated with increased risk should discuss these factors with healthcare providers to determine appropriate monitoring and risk-reduction strategies.  In summary, while environmental factors like smoking remain the primary causes of lung cancer, hereditary components can significantly influence individual risk. Awareness of family history and genetic factors is crucial for early detection and prevention efforts. |
| 1. **What are the types of pathological diagnoses for lung cancer?** | Lung cancer is pathologically classified based on the appearance and characteristics of tumor cells under a microscope. The primary pathological types are:  **Non-Small Cell Lung Cancer (NSCLC):** This category accounts for approximately 80% to 85% of lung cancers and includes several subtypes:   1. Adenocarcinoma: The most common subtype, often originating in peripheral lung tissue. It is also prevalent among non-smokers. 2. Squamous Cell Carcinoma: Typically arises in the central parts of the lungs, near the bronchi. 3. Large Cell Carcinoma: A less common subtype that can occur in any part of the lung and tends to grow rapidly.   **Small Cell Lung Cancer (SCLC):** Representing about 10% to 15% of lung cancers, SCLC is known for its rapid growth and early spread to other body parts.  **Other Types:**   1. Adenosquamous Carcinoma: A rare tumor containing both adenocarcinoma and squamous cell carcinoma components. 2. Combined Small-Cell Lung Carcinoma: A variant of SCLC that includes components of non-small cell carcinoma.   Accurate pathological diagnosis is crucial for determining the most effective treatment strategies for lung cancer patients. |
| 1. **What are the characteristics of different types of lung cancer?** | Lung cancer is primarily categorized into two main types: non-small cell lung cancer (NSCLC) and small cell lung cancer (SCLC), each with distinct characteristics.  **Non-Small Cell Lung Cancer (NSCLC):**  NSCLC accounts for approximately 80% to 85% of lung cancer cases and encompasses several subtypes:   1. Adenocarcinoma: This is the most common subtype of NSCLC, often originating in the outer regions of the lungs. It is more frequently diagnosed in individuals who have never smoked and tends to grow slower than other lung cancers. 2. Squamous Cell Carcinoma: Typically linked to a history of smoking, this subtype usually develops in the central parts of the lungs, near the bronchi. It is characterized by flat, thin cells resembling the lining of the airways. 3. Large Cell Carcinoma: Known for its rapid growth and tendency to spread, large cell carcinoma can appear in any part of the lung. Due to its undifferentiated nature, it is challenging to treat.   **Small Cell Lung Cancer (SCLC):**  SCLC constitutes about 10% to 15% of lung cancers and is strongly associated with cigarette smoking. It is characterized by small cells that multiply quickly, leading to rapid tumor growth and early metastasis. Due to its aggressive nature, SCLC often requires prompt treatment, typically involving chemotherapy and radiation therapy.  **Other Types:**  Lung Carcinoid Tumors: These are rare, accounting for fewer than 5% of lung tumors. Carcinoid tumors generally grow slowly and are less likely to spread.  Understanding these distinct types is crucial, as they differ in growth patterns, spread, and responses to treatment. Accurate diagnosis informs appropriate therapeutic strategies and impacts prognosis. |
| 1. **What are the common metastasis sites of lung cancer?** | Lung cancer frequently metastasizes to various parts of the body. The most common sites include:   1. Lymph Nodes: Cancer cells often first spread to nearby lymph nodes before reaching distant organs. 2. Liver: Metastasis to the liver is common and can impair liver function. 3. Bones: The spine, pelvis, and long bones are frequently affected, leading to pain and fractures. 4. Brain: Brain metastases can cause neurological symptoms such as headaches and seizures. 5. Adrenal Glands: These glands are common sites for metastasis, though they often do not cause noticeable symptoms.   Understanding these common metastasis sites is crucial for early detection and management of metastatic lung cancer. |
| **Topic:** **Diagnosis** | |
| 1. **What are the early symptoms of lung cancer?** | Early-stage lung cancer often presents with subtle or no symptoms, making early detection challenging. However, some individuals may experience the following signs:   1. Persistent Cough: A cough that does not go away or worsens over time. 2. Shortness of Breath: Difficulty breathing or becoming easily winded during routine activities. 3. Chest Pain: Discomfort or pain in the chest that may worsen with deep breathing, coughing, or laughing. 4. Hoarseness: Changes in the voice, such as becoming raspy or hoarse. 5. Unexplained Weight Loss: Losing weight without trying or a noticeable decrease in appetite. 6. Fatigue: Feeling unusually tired or weak without a clear reason. 7. Frequent Respiratory Infections: Recurring infections like bronchitis or pneumonia that are difficult to treat.   It's important to note that these symptoms can also be associated with other conditions. If you or someone you know experiences any of these symptoms persistently, especially if there's a history of smoking or exposure to risk factors, it's crucial to consult a healthcare provider for evaluation. Early detection of lung cancer can significantly improve treatment outcomes.navlistRising Incidence of Lung Cancer Among Non-Smokers, Especially |
| 1. **What are the diagnostic criteria for lung cancer?** | Diagnosing lung cancer involves a comprehensive evaluation combining medical history, physical examination, imaging studies, and tissue sampling. The primary diagnostic criteria include:  **Medical History and Physical Examination:**  A thorough assessment of the patient's medical history, risk factors (such as smoking), and a physical examination to identify any signs indicative of lung cancer.  **Imaging Studies:**   1. Chest X-ray: Often the initial imaging test, it can reveal masses or abnormalities in the lungs. However, its sensitivity is limited, and further imaging is usually required. 2. Computed Tomography (CT) Scan: Provides detailed cross-sectional images of the chest, helping to identify the size, shape, and location of lung tumors. 3. Positron Emission Tomography (PET) Scan: Detects areas of increased metabolic activity, which may indicate cancerous growths.   **Tissue Sampling (Biopsy):**  A definitive diagnosis requires obtaining a tissue sample for microscopic examination:   1. Bronchoscopy: Insertion of a flexible tube through the airway to visualize the lungs and collect tissue samples. 2. Needle Biopsy: Using imaging guidance, a needle is inserted through the chest wall to obtain tissue from the suspicious area. 3. Surgical Biopsy: In certain cases, a more invasive procedure may be necessary to acquire an adequate tissue sample.   **Laboratory Tests:**  Analysis of blood and other bodily fluids can provide additional information about overall health and organ function.  **Molecular Testing:**  Identifies specific genetic mutations or markers in cancer cells, guiding targeted therapy decisions.  Early detection through appropriate screening, especially for high-risk individuals, is crucial for improving treatment outcomes. |
| 1. **What are the risk factors for lung cancer?** | Lung cancer risk is influenced by several factors, including:   1. Smoking: The leading risk factor, responsible for about 80% to 90% of lung cancer deaths. 2. Secondhand Smoke Exposure: Increases lung cancer risk in non-smokers. 3. Radon Exposure: A naturally occurring gas that can accumulate indoors, leading to increased lung cancer risk. 4. Occupational Carcinogens: Exposure to substances like asbestos, arsenic, and diesel exhaust can elevate lung cancer risk. 5. Air Pollution: Long-term exposure to polluted air contributes to lung cancer development. 6. Family History: Having a parent, sibling, or child with lung cancer increases one's risk. 7. Previous Radiation Therapy: Radiation treatments to the chest can heighten lung cancer risk. 8. Diet and Lifestyle: Diets high in fats and sugars may promote lung cancer growth. Understanding these risk factors is crucial for prevention and early detection of lung cancer.Lung Cancer Cases Among Non-Smokers Linked to Air |
| 1. **What are the auxiliary examinations and assessments that should be performed for lung cancer?** | In addition to standard diagnostic procedures, several auxiliary examinations and assessments can provide valuable information in the diagnosis and management of lung cancer:   1. **Laboratory Tests:**    1. Complete Blood Count (CBC): Evaluates levels of red and white blood cells and platelets, which can indicate overall health and detect potential anemia or infections.    2. Blood Chemistry Tests: Assess organ function by measuring substances like electrolytes, liver enzymes, and calcium levels.    3. Tumor Markers: Analyzing pleural fluid for tumor markers such as carcinoembryonic antigen (CEA) can aid in identifying malignancies. 2. **Sputum Cytology:**   Microscopic examination of mucus expelled from the lungs can detect cancer cells, particularly in centrally located tumors.   1. **Immunohistochemistry (IHC):**   Utilizes antibodies to detect specific antigens in tissue samples, aiding in tumor classification and guiding treatment strategies.   1. **Molecular Diagnostics:**   Identifies genetic mutations or alterations in cancer cells, informing targeted therapy decisions.   1. **Pulmonary Function Tests (PFTs):**   Assess lung capacity and function to determine suitability for treatments like surgery.   1. **Exhaled Breath Analysis:**   Emerging techniques analyze volatile organic compounds in breath to monitor treatment responses, offering a non-invasive assessment method.  These auxiliary assessments complement primary diagnostic methods, providing a comprehensive understanding of lung cancer and facilitating personalized treatment approaches. |
| 1. **What is the role of CT scan in the diagnosis of lung cancer?** | Computed Tomography (CT) scans play a pivotal role in the diagnosis and management of lung cancer through several key functions:   1. **Detection and Screening:**   Low-Dose CT (LDCT) Screening: For individuals at high risk of lung cancer, such as long-term smokers, LDCT scans are employed to detect early-stage tumors before symptoms emerge. Research indicates that annual LDCT screenings can reduce lung cancer mortality by approximately 20% compared to standard chest X-rays.   1. **Diagnosis:**    1. Identifying Abnormalities: CT scans provide detailed cross-sectional images of the lungs, enabling the identification of suspicious nodules or masses that may not be visible on standard chest X-rays.    2. Guiding Biopsies: When abnormalities are detected, CT imaging assists in precisely guiding biopsy procedures to obtain tissue samples for histological examination, essential for confirming a lung cancer diagnosis. 2. **Staging:**    1. Assessing Tumor Extent: CT scans evaluate the size, location, and extent of the primary tumor, including its relationship to adjacent structures.    2. Detecting Metastasis: They are instrumental in identifying the spread of cancer to lymph nodes or distant organs, crucial for accurate staging and treatment planning. 3. **Treatment Planning and Monitoring:**    1. Planning Interventions: Detailed imaging from CT scans aids in formulating surgical approaches, radiation therapy targeting, and other treatment strategies.    2. Evaluating Treatment Response: Subsequent CT scans monitor the effectiveness of treatments by assessing changes in tumor size and the emergence of new lesions.   While CT scans are invaluable in the diagnostic process, it's important to note that a definitive lung cancer diagnosis requires a biopsy. CT imaging findings guide clinicians in determining the most appropriate sites for tissue sampling.  In summary, CT scans are integral to the early detection, accurate diagnosis, precise staging, and effective management of lung cancer, significantly contributing to improved patient outcomes. |
| 1. **What is the role of genetic testing in the diagnosis of lung cancer?** | Genetic testing plays a crucial role in the diagnosis and management of lung cancer, particularly non-small cell lung cancer (NSCLC), by identifying specific genetic mutations within tumor cells. This information enables personalized treatment strategies and can influence prognosis. The key aspects of genetic testing in lung cancer include:   1. **Identification of Driver Mutations:**   Genetic testing detects specific mutations in cancer cells that drive tumor growth. Common mutations in NSCLC include:   - 1. EGFR (Epidermal Growth Factor Receptor): Mutations in the EGFR gene can promote cancer cell proliferation. Identifying these mutations is essential, as tumors with EGFR alterations often respond well to EGFR tyrosine kinase inhibitors (TKIs).   2. ALK (Anaplastic Lymphoma Kinase): Rearrangements in the ALK gene are found in a subset of NSCLC patients. Targeted therapies, such as ALK inhibitors, have shown effectiveness in treating cancers with these alterations.   3. KRAS: Mutations in the KRAS gene are among the most common in NSCLC. While historically challenging to target, recent advancements have led to the development of therapies aimed at specific KRAS mutations.  1. **Guiding Treatment Decisions:**   The identification of specific genetic mutations through testing allows oncologists to tailor treatment plans:   - 1. Targeted Therapy: Patients with identifiable mutations may benefit from targeted therapies designed to inhibit the function of the mutated proteins, leading to improved outcomes and reduced side effects compared to traditional chemotherapy.   2. Clinical Trials: Genetic testing results can qualify patients for clinical trials investigating novel therapies aimed at specific genetic alterations, providing access to cutting-edge treatments.  1. **Prognostic Implications:**   Certain genetic mutations can provide information about the aggressiveness of the cancer and potential responses to specific treatments, aiding in prognosis determination.     1. **Types of Genetic Testing:**   Several methods are employed to analyze genetic mutations in lung cancer:   - 1. Next-Generation Sequencing (NGS): This comprehensive approach allows for the simultaneous analysis of multiple genes, providing a broad genetic profile of the tumor.   2. Polymerase Chain Reaction (PCR): A more targeted method used to detect specific known mutations within genes.  1. **Timing and Sample Collection:**   Genetic testing is typically performed on tumor tissue obtained during a biopsy or surgery. In some cases, circulating tumor DNA (ctDNA) from blood samples, known as liquid biopsies, can be analyzed, offering a less invasive testing option.  In summary, genetic testing is integral to the modern management of lung cancer, enabling personalized treatment approaches that improve patient outcomes. It assists in identifying actionable mutations, guiding therapy choices, and providing prognostic information, thereby playing a vital role in comprehensive cancer care. |
| **Topic:** **Treatment** | |
| 1. **What are the treatment methods for lung cancer?** | Lung cancer treatment strategies are tailored to the cancer's type (non-small cell lung cancer [NSCLC] or small cell lung cancer [SCLC]), stage, and the patient's overall health. The primary treatment modalities include:  **Surgery**   1. Purpose: To remove cancerous tissue, typically employed in early-stage NSCLC. 2. Procedures:    1. Lobectomy: Removal of an entire lobe of the lung.    2. Pneumonectomy: Removal of an entire lung.    3. Segmentectomy or Wedge Resection: Removal of a small part of the lung.   Surgery may not be suitable if the cancer has metastasized or if the patient has other significant health issues.  **Radiation Therapy**   1. Purpose: To destroy cancer cells or shrink tumors using high-energy radiation. 2. Techniques:    1. External Beam Radiation Therapy (EBRT): Directs radiation from outside the body.    2. Stereotactic Body Radiotherapy (SBRT): Delivers precise, high-dose radiation, often used for small, early-stage tumors.   Radiation therapy can be a primary treatment or used post-surgery to eliminate residual cancer cells.  **Chemotherapy**   1. Purpose: To kill or inhibit the growth of cancer cells using drugs. 2. Application: Often used in combination with other treatments, chemotherapy can be administered before surgery (neoadjuvant) to shrink tumors or after surgery (adjuvant) to target remaining cancer cells.   **Targeted Therapy**   1. Purpose: To attack specific genetic mutations or proteins that promote cancer growth. 2. Examples:    1. EGFR Inhibitors: Such as osimertinib, used for tumors with EGFR mutations.    2. ALK Inhibitors: Such as alectinib, for cancers with ALK gene rearrangements.   Targeted therapies are typically used for advanced NSCLC with specific genetic alterations.  **Immunotherapy**   1. Purpose: To enhance the immune system's ability to recognize and attack cancer cells. 2. Agents:   Checkpoint Inhibitors: Such as pembrolizumab and nivolumab, which block proteins that prevent immune cells from attacking cancer.  Immunotherapy is often used in advanced lung cancers and can be combined with chemotherapy.  **Tumor Treating Fields (TTF) Therapy**   1. Purpose: To disrupt cancer cell division using electric fields. 2. Application: TTF is an emerging treatment modality currently under investigation for its efficacy in lung cancer.   **Clinical Trials**   1. Purpose: To provide access to new and experimental therapies. 2. Consideration: Patients may consider enrolling in clinical trials to access cutting-edge treatments that are not yet widely available.   **Supportive/Palliative Care**   1. Purpose: To alleviate symptoms and improve quality of life. 2. Services: Include pain management, nutritional support, and psychological counseling.   Treatment plans are individualized based on the cancer's characteristics and the patient's health status. A multidisciplinary team approach ensures comprehensive care tailored to each patient's needs. |
| 1. **Is further treatment needed after surgery?** | After lung cancer surgery, additional treatments, known as adjuvant therapies, may be recommended to reduce the risk of cancer recurrence by targeting any remaining cancer cells. The necessity and type of adjuvant therapy depend on factors such as the cancer's stage, specific genetic mutations, and the patient's overall health. Common adjuvant therapies include:   1. **Chemotherapy:**   Administered to eliminate residual cancer cells post-surgery, chemotherapy has been shown to improve survival rates in patients with completely resected non-small cell lung cancer (NSCLC).   1. **Immunotherapy:**   This approach enhances the immune system's ability to target cancer cells. For instance, the FDA approved atezolizumab as an adjuvant treatment for certain patients with early-stage NSCLC to reduce the risk of recurrence.   1. **Targeted Therapy:**   For tumors with specific genetic mutations, targeted therapies can be effective. For example, osimertinib has been approved for routine use in patients with EGFR mutation-positive NSCLC after surgery, demonstrating significant improvements in disease-free survival.  The decision to pursue adjuvant therapy is personalized, considering the potential benefits and risks. It's essential to discuss with your healthcare team to determine the most appropriate post-surgical treatment plan based on your specific circumstances. |
| 1. **In what situations is targeted therapy for lung cancer applicable?** | Targeted therapies are most effective in tumors harboring particular genetic changes. Common actionable mutations in NSCLC include:   1. EGFR (Epidermal Growth Factor Receptor) Mutations: Approximately 10-15% of NSCLC patients in Western countries and up to 50% in Asian populations have EGFR mutations. Patients with these mutations may benefit from EGFR inhibitors such as osimertinib. 2. ALK (Anaplastic Lymphoma Kinase) Rearrangements: Found in about 5% of NSCLC cases, ALK rearrangements can be targeted with ALK inhibitors like alectinib. 3. KRAS G12C Mutations: KRAS mutations are present in approximately 25% of NSCLC cases. Sotorasib is an approved therapy targeting the KRAS G12C mutation.   **Advanced or Metastatic Disease:**  Targeted therapies are commonly utilized in cases where lung cancer has advanced locally or metastasized to other parts of the body. These therapies can be used alone or in combination with other treatments, depending on the specific genetic alterations present.  **Post-Surgical Recurrence:**  In instances where cancer recurs after initial surgery or radiation therapy, targeted therapies may be considered, especially if new genetic mutations are identified.  **Patient's Overall Health:**  For patients who may not tolerate traditional chemotherapy due to other health conditions, targeted therapies can offer a more tailored treatment option with potentially fewer side effects.  It's essential to conduct comprehensive biomarker testing to identify specific genetic mutations before initiating targeted therapy. This personalized approach ensures that the chosen treatment aligns with the tumor's unique characteristics, thereby improving efficacy and minimizing unnecessary side effects.  In summary, targeted therapy is applicable in lung cancer cases where specific genetic mutations are present, particularly in advanced or metastatic stages, or upon recurrence after initial treatments. Thorough molecular profiling of the tumor is crucial to determine the most appropriate targeted therapeutic strategy. |
| 1. **How effective is chemotherapy for lung cancer?** | Chemotherapy plays a pivotal role in the management of lung cancer, with its effectiveness varying based on the cancer type, stage, and individual patient factors.  **Non-Small Cell Lung Cancer (NSCLC):**   1. Early-Stage (I-II): In certain cases, chemotherapy is administered post-surgery (adjuvant chemotherapy) to eradicate residual cancer cells, potentially enhancing survival rates. A meta-analysis indicated a 4% absolute increase in 5-year survival for patients receiving adjuvant chemotherapy after surgical resection. 2. Locally Advanced (Stage III): Chemotherapy, often combined with radiation therapy, is standard for unresectable stage III NSCLC, aiming to improve survival outcomes. 3. Advanced or Metastatic (Stage IV): Chemotherapy can alleviate symptoms, control disease progression, and enhance quality of life. While it may not cure the cancer, it can extend survival and provide symptomatic relief.   **Small Cell Lung Cancer (SCLC):**  Chemotherapy is the cornerstone of treatment for SCLC due to its rapid growth and early spread. It has been shown to improve both quality of life and survival duration. Without treatment, SCLC has a poor prognosis, but chemotherapy can significantly extend survival.  **Combination with Other Therapies:**  Chemotherapy is frequently used alongside other treatments:   1. With Radiation Therapy: Combining chemotherapy with radiation may enhance the effectiveness of both treatments, particularly in certain stages of lung cancer. 2. With Immunotherapy: In advanced NSCLC, chemotherapy is often combined with immunotherapy agents, improving overall survival compared to chemotherapy alone.     **Duration and Administration:**  The length of chemotherapy treatment varies:   1. Adjuvant and Neoadjuvant Settings: Typically administered for 3 to 4 months, depending on the specific drugs used. 2. Advanced Disease: Treatment duration depends on the individual's response and tolerance to therapy.   **Side Effects:**  Chemotherapy can cause side effects such as fatigue, nausea, hair loss, and increased infection risk. The specific side effects depend on the drugs used and individual patient factors.  In summary, chemotherapy remains a fundamental component in treating both NSCLC and SCLC, offering benefits in survival and symptom management. Its effectiveness is influenced by various factors, including cancer stage and patient health, necessitating a personalized treatment approach. |
| 1. **In what situations is radiotherapy for lung cancer applicable?** | Radiotherapy, or radiation therapy, is a pivotal treatment modality for lung cancer, employing high-energy x-rays to target and destroy cancer cells. Its application varies based on the type and stage of lung cancer, as well as individual patient factors.  **Non-Small Cell Lung Cancer (NSCLC):**   1. Early-Stage (Stage I): For patients who are not surgical candidates due to medical comorbidities or personal preference, stereotactic body radiation therapy (SBRT) offers a non-invasive alternative. SBRT delivers high doses of radiation with precision, effectively controlling or eliminating tumors. 2. Locally Advanced (Stages II-III): Radiotherapy is often combined with chemotherapy to enhance treatment efficacy. This concurrent approach aims to control the primary tumor and address regional lymph node involvement. 3. Post-Surgical (Adjuvant) Therapy: In cases where surgical margins are positive or there's involvement of mediastinal lymph nodes (N2 disease), postoperative radiotherapy may be considered to reduce the risk of local recurrence.   **Small Cell Lung Cancer (SCLC):**   1. Limited Stage: Radiotherapy to the chest is commonly administered alongside chemotherapy, aiming for curative intent. This combined modality treatment has been shown to improve survival outcomes. 2. Extensive Stage: While chemotherapy remains the primary treatment, radiotherapy can be utilized for palliation, addressing symptoms such as pain, bleeding, or airway obstruction.   **Prophylactic Cranial Irradiation (PCI):**  Given the high propensity for SCLC to metastasize to the brain, PCI is employed to reduce the risk of cerebral metastases, thereby potentially prolonging survival.  **Palliative Radiotherapy:**  Regardless of lung cancer type, radiotherapy serves a crucial role in palliation, alleviating symptoms such as pain, hemoptysis, or dyspnea, thereby enhancing the patient's quality of life.  **Technological Advancements:**  Modern radiotherapy techniques, including intensity-modulated radiation therapy (IMRT) and image-guided radiation therapy (IGRT), have improved the precision of treatment delivery, minimizing exposure to surrounding healthy tissues and reducing side effects.  **Considerations and Side Effects:**  Potential side effects of thoracic radiotherapy include fatigue, esophagitis, and radiation pneumonitis. These risks necessitate a thorough discussion between the patient and the oncology team to weigh the benefits and potential adverse effects of treatment.  In summary, radiotherapy's role in lung cancer is multifaceted, encompassing curative, adjuvant, and palliative intents, tailored to the individual patient's clinical scenario. |
| 1. **What is immunotherapy?** | Immunotherapy is a type of biological therapy that utilizes the body's immune system to combat diseases, including cancer. It functions by enhancing or modifying the immune system's ability to recognize and attack cancer cells.  **How Immunotherapy Works:**  The immune system naturally identifies and destroys abnormal cells. However, cancer cells can develop mechanisms to evade immune detection. Immunotherapy aims to bolster the immune system's capacity to detect and eliminate these malignant cells.  **Types of Immunotherapy:**   1. Checkpoint Inhibitors: These drugs block proteins that inhibit immune responses, thereby allowing T-cells to effectively attack cancer cells. 2. CAR T-Cell Therapy: This approach involves modifying a patient's T-cells to better recognize and target cancer cells. 3. Cancer Vaccines: Vaccines can stimulate the immune system to recognize and attack specific cancer antigens.   **Applications in Cancer Treatment:**  Immunotherapy has shown promise across various cancer types, including melanoma, lung cancer, and certain lymphomas. It can be used alone or in conjunction with other treatments like chemotherapy and radiation therapy.  **Recent Advancements:**  Innovations in immunotherapy have led to significant breakthroughs. For example, a clinical trial at the Melanoma Institute Australia demonstrated the potential to "cure" advanced melanoma using a combination of immunotherapy drugs, nivolumab and ipilimumab.  **Considerations:**  While immunotherapy offers substantial benefits, it may not be suitable for all patients. Potential side effects and individual health factors must be carefully evaluated by healthcare professionals.  In summary, immunotherapy represents a significant advancement in cancer treatment, harnessing the body's immune system to fight cancer more effectively. |
| **Topic:** **Prognosis** | |
| 1. **What is the frequency of follow-up after lung cancer surgery?** | After lung cancer surgery, a structured follow-up plan is essential to monitor for potential recurrence and manage any arising complications. The frequency and nature of these follow-up visits can vary based on individual circumstances and the healthcare provider's protocol. However, general guidelines suggest the following schedule:  **Initial Follow-Up:**  to 6 Weeks Post-Surgery: An initial follow-up appointment is typically scheduled within this timeframe. During this visit, the surgeon will:   - 1. Discuss the surgical outcomes and pathology results.   2. Conduct a physical examination.   3. Address any postoperative concerns or complications.   This appointment also provides an opportunity for patients to ask questions and receive guidance on recovery.  **Subsequent Surveillance:**   1. First 2 Years: Follow-up visits are commonly recommended every 3 to 6 months. These appointments may include:    1. A thorough medical history review and physical examination.    2. Imaging studies, such as chest computed tomography (CT) scans, to detect any signs of recurrence or new primary lung cancers.   The American Society of Clinical Oncology (ASCO) advises surveillance imaging every 6 months during this period.   1. Years 3 to 5: If no recurrence is detected, the frequency of follow-up visits may decrease to every 6 to 12 months. Annual imaging studies are typically performed to continue monitoring. 2. After 5 Years: Annual visits may suffice for ongoing surveillance, focusing on overall health and any late effects of treatment.   **Considerations:**   1. Individual Risk Factors: Patients with higher-stage tumors or other risk factors may require more frequent follow-ups. 2. Imaging Modality: High-resolution CT scans are preferred over chest X-rays due to their superior sensitivity in detecting recurrences or new primary lung cancers. 3. Patient Health Status: The intensity of follow-up should be tailored to the patient's overall health and ability to undergo further treatment if necessary.   It's important to note that these guidelines can vary, and patients should engage in shared decision-making with their healthcare team to develop a follow-up plan that aligns with their specific needs and circumstances. |
| 1. **What should be done if a new nodule is found during follow-up?** | The discovery of a new pulmonary nodule during follow-up after lung cancer surgery necessitates a thorough evaluation to determine its nature and appropriate management. The following steps are typically considered:  **Comprehensive Assessment:**   1. Patient History and Risk Factors: Evaluate the patient's smoking history, exposure to carcinogens, family history of cancer, and any previous malignancies. These factors can influence the likelihood of the nodule being malignant. 2. Nodule Characteristics: Analyze the size, shape, location, and growth rate of the nodule. Larger nodules and those exhibiting growth over time are more concerning for malignancy.   **Imaging Studies:**   1. High-Resolution CT Scan: A detailed CT scan provides precise information about the nodule's size, margins, and density, aiding in risk stratification. 2. PET-CT Scan: This imaging modality assesses metabolic activity. Nodules with increased uptake may indicate malignancy, though false positives can occur due to inflammatory processes.   **Biopsy Procedures:**   1. Image-Guided Needle Biopsy: For accessible nodules, a percutaneous needle biopsy can obtain tissue samples for histopathological examination. 2. Bronchoscopy with Biopsy: If the nodule is near the central airways, bronchoscopy allows for direct visualization and sampling. 3. Surgical Biopsy: In certain cases, minimally invasive surgical techniques like video-assisted thoracoscopic surgery (VATS) may be employed to remove and diagnose the nodule.   **Multidisciplinary Evaluation:**  A team comprising thoracic surgeons, pulmonologists, radiologists, and oncologists should collaboratively review the findings to formulate an individualized management plan.  **Management Strategies:**   1. Benign Nodules: If the nodule is determined to be benign, regular surveillance with periodic imaging may be sufficient. 2. Malignant Nodules: For confirmed malignancies, treatment options include surgical resection, radiation therapy, or systemic therapies, depending on the patient's overall health and cancer staging. 3. Indeterminate Nodules: When the nature of the nodule remains uncertain, a short-term follow-up with repeat imaging or a biopsy may be recommended.   In conclusion, the detection of a new pulmonary nodule post-lung cancer surgery requires a systematic approach, integrating patient history, imaging studies, possible biopsy, and multidisciplinary input to ensure accurate diagnosis and optimal management. |
| 1. **How to determine whether lung cancer recurs during follow-up?** | Detecting a recurrence of lung cancer during follow-up involves a combination of regular medical evaluations, imaging studies, and patient vigilance for specific symptoms. Here's an overview of the recommended approach:  **Scheduled Surveillance Imaging:**   1. Frequency: Patients should undergo surveillance imaging every 6 months for the first 2 years post-treatment, followed by annual scans thereafter. 2. Imaging Modality: Chest computed tomography (CT) scans are preferred due to their sensitivity in detecting recurrences or new primary lung cancers.   **Regular Clinical Evaluations:**   1. Physical Examinations: Routine visits to a healthcare provider for physical assessments can help identify signs of recurrence. 2. Symptom Assessment: Patients should report new or worsening symptoms such as:    1. Chronic cough    2. Coughing up blood    3. Breathing difficulties (e.g., wheezing, shortness of breath)    4. Unexplained weight loss    5. Persistent fatigue    6. Dizziness or visual disturbances 3. Advanced Diagnostic Tools: 3. Circulating Tumor DNA (ctDNA) Analysis: Emerging techniques like Cancer Personalized Profiling by Deep Sequencing (CAPP-Seq) can detect ctDNA in the bloodstream, offering a non-invasive method to monitor tumor burden and identify recurrences. 4. Artificial Intelligence (AI) in Imaging: Machine learning models are being developed to assist in the detection and analysis of pulmonary nodules in follow-up CT scans, potentially enhancing early identification of recurrences.   **Multidisciplinary Approach:**  Engaging a team of specialists—including oncologists, radiologists, and pulmonologists—ensures comprehensive evaluation and management of any suspected recurrence.  **Patient Education and Self-Monitoring:**  Educating patients about potential recurrence symptoms empowers them to seek timely medical attention, facilitating early detection and treatment.  Implementing a structured follow-up plan that incorporates these strategies is crucial for the early detection of lung cancer recurrence, which can significantly improve the chances of successful retreatment and survival. |
| 1. **How to manage lifestyle after lung cancer surgery?** | After lung cancer surgery, adopting a healthy lifestyle is crucial for recovery, reducing the risk of recurrence, and enhancing overall well-being. Key considerations include:  **Smoking Cessation:**  If you smoke, quitting is imperative. Continuing to smoke after a lung cancer diagnosis can adversely affect treatment outcomes and increase the risk of recurrence. Resources and support systems are available to assist in quitting.  **Nutrition:**  Maintaining a balanced diet supports healing and overall health. Recommendations include:   1. Soft and Easy-to-Chew Foods: Post-surgery discomfort may make swallowing challenging; opt for foods that are gentle on the throat. 2. Small, Frequent Meals: Eating smaller portions more frequently can aid digestion and ensure adequate nutrient intake. 3. Avoid Irritating Foods: Steer clear of spicy or acidic foods that may irritate the digestive tract. 4. Nutritional Supplements: Consult your healthcare provider about supplements to address specific nutritional needs.   **Physical Activity:**  Engaging in regular physical activity can improve mood, energy levels, and overall health. Suggestions include:   1. Start Slowly: Begin with light activities, such as walking, and gradually increase intensity as tolerated. 2. Incorporate Strength Training: Building muscle strength has been linked to reduced mortality in cancer patients. 3. Consult Your Doctor: Before starting any exercise program, discuss it with your healthcare provider to ensure safety.   **Emotional Well-being:**  Addressing the emotional impact of cancer is vital:   1. Seek Support: Connect with support groups, counselors, or social workers to navigate complex emotions. 2. Stress Management: Engage in activities like meditation, journaling, or hobbies to manage stress.   **Follow-Up Care:**  Regular medical check-ups are essential to monitor recovery and detect any signs of recurrence:   1. Adhere to Appointments: Keep all scheduled follow-up visits with your healthcare team. 2. Report New Symptoms: Inform your doctor promptly about any new or worsening symptoms.   Implementing these lifestyle changes can significantly impact your recovery and quality of life after lung cancer surgery. Always consult your healthcare team before making any significant changes to your lifestyle or treatment plan. |
| 1. **Whether the focus of follow-up for long-term survivors (e.g., more than 5 years after surgery) needs to be adjusted?** | For long-term survivors of lung cancer, particularly those beyond 5 years post-surgery, follow-up care remains crucial but may be adjusted to focus on the detection of new primary lung cancers rather than recurrence. Here's how the focus of follow-up may be tailored:  **Surveillance Imaging:**  Annual Low-Dose CT Scans: After the initial 5-year period, many guidelines recommend transitioning to annual low-dose computed tomography (LDCT) scans. This approach aims to detect new primary lung cancers at an early, more treatable stage.  **Clinical Evaluations:**  Regular Check-Ups: Continued periodic visits to your healthcare provider are important to monitor overall health, manage any chronic conditions, and promptly address new symptoms that could indicate a recurrence or a new cancer.  **Lifestyle Modifications:**   1. Smoking Cessation: If you smoke, quitting remains vital to reduce the risk of new lung cancers. 2. Healthy Lifestyle: Maintaining a balanced diet and regular physical activity supports overall health and may lower cancer risk.   **Personalized Follow-Up Plan:**  Individualized Approach: Collaborate with your healthcare team to develop a follow-up plan tailored to your specific risk factors, overall health, and preferences.  In summary, while the intensity of follow-up may decrease after 5 years, ongoing annual LDCT scans and regular clinical evaluations remain essential to detect new primary lung cancers early. Engaging in healthy lifestyle practices and maintaining open communication with your healthcare team further support long-term health. |
| 1. **How to manage long-term side effects after treatment, such as lung function damage, radiation pneumonitis, etc.** | Managing long-term side effects after lung cancer treatment, such as reduced lung function and radiation pneumonitis, is crucial for maintaining quality of life. Here's how these conditions can be addressed:  **Impaired Lung Function:**   1. Pulmonary Rehabilitation: Engage in structured exercise programs designed to improve respiratory efficiency and overall physical endurance. 2. Breathing Exercises: Techniques like diaphragmatic breathing can enhance lung capacity and reduce breathlessness. 3. Medications: Bronchodilators or inhaled corticosteroids may be prescribed to alleviate respiratory symptoms.   **Radiation Pneumonitis:**   1. Observation: Mild cases might resolve without intervention and can be monitored for spontaneous improvement. 2. Corticosteroids: For moderate to severe symptoms, oral prednisone is commonly administered to reduce lung inflammation. A typical regimen starts with 1 mg/kg per day for 2 to 4 weeks, followed by a gradual taper over 6 to 12 weeks. 3. Supportive Measures: Using humidifiers, maintaining adequate hydration, and avoiding respiratory irritants can help manage symptoms.   **General Strategies for Long-Term Side Effect Management:**   1. Regular Follow-Ups: Attend scheduled appointments to monitor and address any emerging issues promptly. 2. Healthy Lifestyle: Maintain a balanced diet and engage in appropriate physical activity to support overall health and recovery. 3. Emotional Support: Seek counseling or join support groups to address the psychological impact of long-term side effects.   Collaborating closely with your healthcare team is essential to tailor interventions to your specific needs and ensure optimal management of long-term side effects. |
| **Topic:** **Case** | |
| 1. **A 68-year-old male patient, with a 40-year history of smoking, has been experiencing cough and blood-tinged sputum for 3 months. Chest X-ray showed an irregular mass in the right upper lung with a rough edge; CT scan suggested that the mass invaded the pleura and mediastinal lymph nodes were enlarged. Lung cancer is initially considered, and bronchoscopy and biopsy are recommended to confirm the pathology. What further tests does the patient need to confirm the diagnosis?** | Given the patient's history and imaging findings, a comprehensive diagnostic approach is essential to confirm the diagnosis and determine the extent of the disease. In addition to bronchoscopy and biopsy, the following evaluations are recommended:   1. **Positron Emission Tomography (PET) Scan:**   A PET scan involves injecting a small amount of radioactive sugar into the bloodstream. Cancer cells, due to their higher metabolic activity, absorb more of this sugar, making them detectable on the scan. This imaging modality helps in assessing the metabolic activity of the lung mass and identifying potential metastasis.   1. **Mediastinoscopy:**   This procedure involves inserting a thin, lighted tube through a small incision at the base of the neck to examine and biopsy the mediastinal lymph nodes. It provides a definitive assessment of lymph node involvement, which is crucial for accurate staging.   1. **Endobronchial Ultrasound (EBUS):**   EBUS combines bronchoscopy with ultrasound to visualize structures adjacent to the airways. It allows for real-time needle aspiration of suspicious lymph nodes or masses, aiding in diagnosis and staging.   1. **Magnetic Resonance Imaging (MRI):**   An MRI of the brain is recommended to detect any potential metastases, especially in cases where neurological symptoms are present. MRI provides detailed images of soft tissues and is particularly useful for brain imaging.   1. **Pulmonary Function Tests (PFTs):**   PFTs assess lung capacity and function, providing baseline data that is essential for evaluating the patient's ability to tolerate potential treatments, such as surgery or radiation therapy.   1. **Laboratory Tests:**   Comprehensive blood work, including a complete blood count (CBC) and liver and kidney function tests, is necessary to evaluate the patient's overall health and readiness for treatment.  These additional tests will provide a thorough understanding of the tumor's characteristics and the patient's physiological status, facilitating the development of an effective and personalized treatment plan. |
| 1. **A 55-year-old female patient, diagnosed with advanced non-small cell lung cancer (NSCLC), with EGFR gene mutation positive. She has received gefitinib targeted therapy, but recently developed drug resistance and tumor progression. How to adjust the next treatment plan?** | In cases where a patient with advanced non-small cell lung cancer (NSCLC) harboring an EGFR mutation develops resistance to first-generation EGFR tyrosine kinase inhibitors (TKIs) like gefitinib, the treatment strategy should be adjusted based on the underlying resistance mechanisms and the patient's overall health status.   1. **Assess Resistance Mechanisms:**   Molecular Testing: Perform a re-biopsy or liquid biopsy to identify specific genetic alterations responsible for the acquired resistance. The presence of the T790M mutation, for instance, would influence subsequent treatment choices.   1. **Alternative EGFR TKIs:**   Osimertinib: This third-generation EGFR TKI is effective against both sensitizing EGFR mutations and the T790M resistance mutation. If the T790M mutation is detected, osimertinib is the preferred treatment option due to its efficacy and favorable safety profile.   1. **Combination Therapies:**    1. EGFR TKIs Plus Chemotherapy: For patients without the T790M mutation or those who progress on osimertinib, combining EGFR TKIs with platinum-based chemotherapy has shown improved outcomes. This approach may help overcome resistance and enhance treatment efficacy.    2. EGFR TKIs Plus Anti-Angiogenic Agents: Combining EGFR TKIs with angiogenesis inhibitors, such as bevacizumab or ramucirumab, has demonstrated enhanced progression-free survival in clinical trials. This strategy targets both the tumor cells and their blood supply, potentially improving treatment outcomes. 2. **Emerging Therapies:**    1. Amivantamab Plus Lazertinib: Recent studies have shown that the combination of amivantamab, a bispecific antibody targeting EGFR and MET, with lazertinib, a third-generation EGFR TKI, significantly prolongs progression-free survival compared to osimertinib alone. This combination is emerging as a promising option for patients with EGFR-mutant NSCLC.    2. Patritumab Deruxtecan: This antibody-drug conjugate has demonstrated efficacy in extending progression-free survival in patients with EGFR-mutated NSCLC who have developed resistance to prior EGFR TKI therapies. It represents a novel therapeutic avenue for overcoming resistance. 3. **Clinical Trials:**   Participation in Clinical Trials: Enrolling in clinical trials offers access to cutting-edge therapies and novel combinations that may provide additional benefits beyond standard treatments. Patients are encouraged to discuss available clinical trial options with their oncologists.   1. **Supportive Care:**   Symptom Management: Alongside active treatment, addressing symptoms and maintaining quality of life are paramount. Supportive care measures should be integrated into the treatment plan.  In summary, for a 55-year-old female patient with advanced EGFR-mutant NSCLC who has developed resistance to gefitinib, the next steps involve identifying the specific resistance mechanisms through molecular testing and tailoring the treatment plan accordingly. Options include switching to osimertinib if the T790M mutation is present, exploring combination therapies, considering emerging treatments like amivantamab plus lazertinib or patritumab deruxtecan, and evaluating eligibility for clinical trials. A multidisciplinary approach is essential to optimize outcomes and maintain quality of life. |
| 1. **A 70-year-old male patient, on the 3rd day after lung cancer surgery, developed pleural effusion and poor drainage. Body temperature was 37.8°C, heart rate was 90 beats/min, respiratory rate was 22 breaths/min, and the drainage tube was blocked. The patient complained of chest tightness. How to deal with the blocked drainage tube?** | Managing a blocked chest drainage tube in a postoperative lung cancer patient is crucial to prevent complications such as pleural effusion accumulation and respiratory distress. The following steps are recommended:   1. **Assess and Address the Drainage System:**    1. Check for Kinks or Obstructions: Examine the entire length of the chest tube for any visible kinks, bends, or external obstructions that could impede drainage.    2. Milking or Stripping the Tube: Gently manipulate the tube to dislodge potential blockages like blood clots or fibrin. However, this technique is controversial due to potential tissue damage from increased negative pressure and should be performed cautiously.    3. Ensure Secure Connections: Verify that all connections between the chest tube and the drainage system are tight and secure to maintain effective drainage. 2. **Evaluate the Patient's Clinical Status:**    1. Monitor Vital Signs: Regularly assess temperature, heart rate, respiratory rate, and oxygen saturation to detect signs of infection or respiratory compromise.    2. Assess Respiratory Function: Observe for symptoms such as chest tightness, shortness of breath, or decreased breath sounds, which may indicate inadequate drainage or tube malfunction. 3. **Imaging Studies:**   Chest X-Ray or Ultrasound: Perform imaging to evaluate the position of the chest tube and the extent of any residual pleural effusion or pneumothorax.   1. **Interventions for Persistent Blockage:**    1. Tube Clearance: If non-invasive measures fail, consider using sterile techniques to clear the blockage, such as flushing the tube with saline under medical supervision.    2. Tube Replacement: If the tube remains obstructed and the patient's condition does not improve, replacing the chest tube may be necessary to restore proper drainage. 2. **Preventive Measures:**    1. Proper Tube Positioning: Ensure optimal placement of the chest tube during insertion to facilitate effective drainage and reduce the risk of blockage.    2. Regular Monitoring: Implement routine checks of the drainage system to promptly identify and address potential issues.   Prompt identification and management of a blocked chest drainage tube are essential to prevent complications and promote recovery in postoperative lung cancer patients. Close monitoring and timely interventions can significantly improve patient outcomes. |
| 1. **A 48-year-old female patient, newly diagnosed with advanced lung cancer, is feeling low and refusing treatment. The patient has obvious anxiety and depression, worrying about the side effects of treatment and family burden. How to help the patient rebuild treatment confidence?** | A diagnosis of advanced lung cancer can be profoundly distressing, leading to anxiety, depression, and concerns about treatment side effects and the potential burden on family members. Addressing these emotional and psychological challenges is essential to help the patient regain confidence in pursuing treatment. The following strategies may be beneficial:   1. **Psychological Support:**    1. Professional Counseling: Encourage the patient to engage with mental health professionals, such as psychologists or counselors, who specialize in oncology. These professionals can provide coping strategies tailored to the individual's needs.    2. Mindfulness and Relaxation Techniques: Practices like yoga, meditation, and deep-breathing exercises can reduce stress and enhance mental clarity. 2. **Education and Communication:**    1. Detailed Information: Provide comprehensive information about the diagnosis, treatment options, potential side effects, and their management. Understanding what to expect can alleviate fears and uncertainties.    2. Open Dialogue: Encourage the patient to express their concerns and questions. Open communication with the healthcare team fosters trust and ensures that the patient's values and preferences are considered in the treatment plan. 3. **Support Networks:**    1. Support Groups: Connecting with others who have faced similar diagnoses can provide emotional support and practical advice. Support groups offer a sense of community and understanding.    2. Family Involvement: Encourage family participation in discussions and decision-making processes. This involvement can alleviate the patient's feelings of isolation and distribute the emotional burden. 4. **Addressing Practical Concerns:**    1. Financial Counseling: Provide information about financial resources, insurance coverage, and assistance programs to alleviate concerns about the economic impact of treatment.    2. Care Coordination: Offer support in organizing appointments, transportation, and daily activities to reduce the logistical burdens associated with treatment. 5. **Integrative Therapies:**   Complementary Therapies: Integrating therapies such as acupuncture, massage, or art therapy can improve quality of life and provide additional coping mechanisms.   1. **Reassessing Treatment Options:**   Personalized Treatment Plan: Discuss the possibility of tailoring treatment to minimize side effects while effectively managing the disease. This approach can include exploring less intensive therapies or clinical trials offering novel treatments.  By addressing both the emotional and practical concerns of the patient, healthcare providers can help rebuild confidence in the treatment process, ensuring that care aligns with the patient's values and improves their quality of life. |
| 1. **A 65-year-old male patient, diagnosed with locally advanced non-small cell lung cancer, is not suitable for surgical resection. He has completed concurrent chemoradiotherapy, but developed radiation pneumonitis and shortness of breath. How to relieve the symptoms of radiation pneumonitis?** | Radiation pneumonitis is an inflammation of the lung tissue that can occur following radiation therapy, particularly in treatments involving the chest area. Symptoms typically manifest between 4 to 12 weeks after completing radiation therapy and can include cough, fever, shortness of breath, and a general feeling of discomfort or illness (malaise).   1. **Management and Symptom Relief:**    1. Corticosteroids: The primary treatment for symptomatic radiation pneumonitis involves corticosteroids to reduce inflammation. A common regimen starts with oral prednisone at doses ranging from 20 mg to 60 mg daily, depending on severity, followed by a gradual taper over several weeks. It's essential to monitor blood glucose levels during corticosteroid therapy, especially in diabetic patients, and consider prophylaxis for Pneumocystis jiroveci pneumonia (PJP) in those receiving prolonged, high-dose corticosteroids.    2. Bronchodilators: These medications can help open airways, making breathing easier. While evidence is limited, they may provide symptomatic relief in some patients.    3. Cough Suppressants and Decongestants: To manage cough and congestion, over-the-counter or prescribed medications can be utilized. It's advisable to consult the healthcare team before starting these treatments.    4. Oxygen Therapy: For patients experiencing significant shortness of breath or low oxygen levels, supplemental oxygen may be necessary to maintain adequate oxygenation. 2. **Supportive Measures:**    1. Rest: Adequate rest is crucial, especially when experiencing shortness of breath.    2. Positioning: Using an extra pillow to elevate the head and upper body during rest or sleep can facilitate easier breathing.    3. Environmental Control: Avoid exposure to extreme temperatures, as hot, humid, or very cold conditions can irritate the lungs.    4. Clothing: Wearing light, loose-fitting clothing and avoiding tight collars can help reduce discomfort. 3. **Consultation with Specialists:**   Given the complexity of radiation pneumonitis, especially in patients with underlying conditions like lung cancer, involving a respiratory physician can provide specialized care and tailored management strategies.  Early recognition and prompt management of radiation pneumonitis are vital to prevent progression to more severe lung injury, such as pulmonary fibrosis. Close collaboration with the healthcare team ensures that treatment is optimized, and supportive measures are effectively implemented to enhance the patient's quality of life. |

**Supplementary Table 2d:** Copilot Responses to Lung Cancer Questions

| **Question** | **Copilot Response** |
| --- | --- |
| **Topic: Overview** | |
| 1. **What is lung cancer?** | Lung cancer is a type of cancer that begins in the lungs, which are vital organs responsible for taking in oxygen and expelling carbon dioxide. It occurs when abnormal cells in the lung grow uncontrollably, forming tumors. These tumors can interfere with the lung's ability to function properly and may spread to other parts of the body (a process known as metastasis).  **There are two main types of lung cancer:**   1. Non-small cell lung cancer (NSCLC): This is the most common type and includes subtypes like adenocarcinoma, squamous cell carcinoma, and large cell carcinoma. 2. Small cell lung cancer (SCLC): This is less common but tends to grow and spread more rapidly than NSCLC.   Lung cancer is strongly linked to smoking, but it can also occur in non-smokers due to factors like exposure to secondhand smoke, air pollution, radon gas, or genetic predisposition. |
| 1. **What are the clinical features of lung cancer?** | The clinical features of lung cancer can vary depending on the stage of the disease, its type, and the location of the tumor. Common signs and symptoms include:  **Respiratory Symptoms**   1. Persistent cough that doesn't go away or worsens over time. 2. Coughing up blood (hemoptysis), even in small amounts. 3. Shortness of breath or wheezing. 4. Chest pain that may get worse with deep breathing, coughing, or laughing.   **General Symptoms**   1. Unexplained weight loss or loss of appetite. 2. Fatigue or weakness. 3. Fever and recurring respiratory infections, such as pneumonia or bronchitis.   **Advanced Symptoms (if the cancer has spread)**   1. Bone pain, often in the back, hips, or ribs. 2. Neurological symptoms like headaches, dizziness, or seizures if it spreads to the brain. 3. Swelling in the face or neck due to compression of blood flow (often from a tumor pressing on large veins).   Some people with lung cancer might not experience symptoms in the early stages, which is why it's sometimes detected late. |
| 1. **Is lung cancer hereditary?** | Lung cancer itself isn't typically classified as a hereditary disease, but genetics can play a role in increasing a person's risk. Here's how:   1. **Family History:** Having a close relative (like a parent or sibling) with lung cancer may raise your risk, even if you're a nonsmoker. This could be due to shared genetic factors or environmental exposures, like secondhand smoke or radon gas. 2. **Genetic Mutations:** Some individuals may inherit genetic mutations that make their lung cells more vulnerable to damage or that hinder the body's ability to repair damaged cells. For example, mutations in genes like EGFR (Epidermal Growth Factor Receptor) are linked to certain types of lung cancer, though they may not always be inherited. 3. **Smoking and Genes:** Genetics can also influence how your body reacts to carcinogens in tobacco smoke, affecting your risk if you're a smoker.   While genetics can contribute to the risk, lung cancer is more often caused by environmental factors like smoking, air pollution, or exposure to toxic substances. If you have a family history of lung cancer and are concerned, it might be helpful to consult a medical professional for personalized advice and consider regular health screenings. |
| 1. **What are the types of pathological diagnoses for lung cancer?** | Pathological diagnoses for lung cancer are made by analyzing tissue or cell samples under a microscope, often in combination with molecular testing. These diagnoses are essential for determining the type of lung cancer and guiding treatment. The main types of pathological diagnoses are:  **Non-Small Cell Lung Cancer (NSCLC) Subtypes**   1. Adenocarcinoma: The most common type, especially in nonsmokers. It originates in the glandular cells and is usually found in the outer areas of the lungs. 2. Squamous Cell Carcinoma: Often linked to smoking, it arises in the cells lining the airways and is typically located near the central bronchi. 3. Large Cell Carcinoma: A less common type that can occur anywhere in the lung and tends to grow and spread quickly.   **Small Cell Lung Cancer (SCLC)**  Typically associated with smoking, this cancer is aggressive and often presents as "small round blue cells" under a microscope. It's divided into:   - 1. Limited-stage SCLC: Confined to one lung.   2. Extensive-stage SCLC: Spread beyond the lung.   **Carcinoid Tumors**  Rare, slow-growing lung tumors that arise from neuroendocrine cells. These are categorized into:   - 1. Typical Carcinoids: Low grade, less aggressive.   2. Atypical Carcinoids: Intermediate grade, with a slightly higher risk of spreading.   **Other Rare Subtypes**   1. Sarcomatoid Carcinomas: A group of rare, aggressive tumors with both carcinomatous and sarcomatous features. 2. Adenosquamous Carcinoma: Contains both adenocarcinoma and squamous cell components.   **Precursor and Benign Lesions**   1. Atypical Adenomatous Hyperplasia (AAH): A precursor to adenocarcinoma. 2. Benign Tumors: Such as hamartomas, which are noncancerous but may require monitoring.   **Molecular and Biomarker Testing**  Pathological diagnoses often include testing for genetic mutations, such as:   - 1. EGFR, ALK, or KRAS mutations.   2. PD-L1 expression levels, which can guide immunotherapy decisions.   These classifications provide critical insights for creating individualized treatment plans. |
| 1. **What are the characteristics of different types of lung cancer?** | The characteristics of lung cancer types depend on their origin, growth patterns, and behavior. Here's an overview:  **Non-Small Cell Lung Cancer (NSCLC)**   1. Adenocarcinoma:    1. Common in both smokers and non-smokers.    2. Tends to grow in the outer areas of the lungs.    3. Often detected before it spreads, making it more treatable. 2. Squamous Cell Carcinoma:    1. Typically linked to smoking.    2. Originates in the central airways, closer to the bronchus.    3. May cause symptoms like coughing or airway obstruction earlier on. 3. Large Cell Carcinoma:    1. Less common and can occur anywhere in the lungs.    2. Known for rapid growth and spread.   **Small Cell Lung Cancer (SCLC)**   1. Aggressive and fast-growing, often spreading to distant organs early. 2. Closely linked to smoking. 3. Tumors are often centrally located in the chest. 4. Symptoms like weight loss, coughing up blood, or difficulty breathing may appear quickly.   **Carcinoid Tumors**   1. Typical Carcinoids:    1. Slow-growing and less likely to spread.    2. Can occur in younger patients and nonsmokers. 2. Atypical Carcinoids:   Grow faster and have a higher risk of spreading compared to typical carcinoids.  **Metastatic Lung Cancer**   1. Results when cancer spreads to the lungs from other organs. 2. Tumors in this case reflect the characteristics of the primary cancer (e.g., breast, colon).   Each subtype has distinct growth patterns, aggressiveness, and molecular features. Treatments and prognosis vary significantly based on these characteristics. |
| 1. **What are the common metastasis sites of lung cancer?** | Lung cancer can spread (metastasize) to various parts of the body, particularly in advanced stages. Common metastasis sites include:   1. **Lymph Nodes**   Frequently, lung cancer spreads to nearby lymph nodes in the chest and neck regions.   1. **Liver**   Metastasis to the liver is common and might cause symptoms like abdominal pain, nausea, or jaundice. However, liver metastases are often asymptomatic in early stages.   1. **Bones**   The bones, especially the spine, pelvis, and ribs, are frequent sites for lung cancer metastasis. This can result in bone pain, fractures, or elevated calcium levels in the blood (hypercalcemia).   1. **Brain**   The brain is a common site for metastasis, particularly with small cell lung cancer (SCLC) and some adenocarcinomas. Symptoms might include headaches, seizures, dizziness, or cognitive changes.   1. **Adrenal Glands**   The adrenal glands are another frequent location for metastasis. This typically does not cause symptoms unless the spread is significant.   1. **Other Lungs**   Lung cancer may spread from one lung to the other or to the pleura (the lining around the lungs), potentially causing fluid buildup (pleural effusion) and breathing difficulties.  The symptoms of metastasis vary depending on the location affected. Regular follow-up imaging and testing can help detect and manage metastatic lung cancer. |
| **Topic:** **Diagnosis** | |
| 1. **What are the early symptoms of lung cancer?** | The early symptoms of lung cancer can often be subtle or mistaken for other common respiratory conditions. Some signs to watch for include:   1. Persistent Cough: A new cough that doesn't go away or a chronic cough that changes in character. 2. Shortness of Breath: Difficulty breathing during routine activities or at rest. 3. Coughing Up Blood: Even small amounts of blood-tinged mucus can be an early warning. 4. Chest Pain: Discomfort or pain that worsens with deep breathing, coughing, or laughing. 5. Hoarseness: A noticeable change in your voice tone or sound. 6. Frequent Infections: Recurring respiratory infections, such as bronchitis or pneumonia. 7. Unexplained Fatigue: Feeling unusually tired without an obvious reason.   Sometimes, lung cancer in its early stages might not cause symptoms at all, making regular health check-ups important, especially for individuals at higher risk. |
| 1. **What are the diagnostic criteria for lung cancer?** | The diagnostic criteria for lung cancer involve a combination of clinical evaluation, imaging studies, and pathological confirmation. Here's an overview:   1. **Clinical Evaluation**    1. Persistent symptoms such as cough, shortness of breath, chest pain, or hemoptysis (coughing up blood).    2. Risk factors like smoking history, exposure to carcinogens, or family history of lung cancer. 2. **Imaging Studies**    1. Chest X-ray: Often the first imaging test to detect abnormalities.    2. CT Scan: Provides detailed images of the lungs and helps identify the size, shape, and location of tumors.    3. PET Scan: Used to detect cancer spread (metastasis) by highlighting areas of high metabolic activity. 3. **Pathological Confirmation**    1. Biopsy: Tissue samples are obtained through methods like bronchoscopy, needle aspiration, or surgical procedures.    2. Cytology: Examination of sputum or pleural fluid for cancer cells. 4. **Molecular and Genetic Testing**   Testing for specific mutations (e.g., EGFR, ALK, KRAS) or biomarkers (e.g., PD-L1) to guide targeted therapies.   1. **Staging**   Using the TNM classification system (Tumor, Node, Metastasis) to determine the extent of the disease.  These criteria help confirm the diagnosis and guide treatment decisions. |
| 1. **What are the risk factors for lung cancer?** | Lung cancer risk is influenced by a combination of lifestyle, environmental, and genetic factors. Here are the most common ones:   1. **Tobacco Smoking**    1. Cigarette Smoking: The leading cause of lung cancer, responsible for the majority of cases. The risk increases with the number of years and quantity smoked.    2. Secondhand Smoke: Non-smokers exposed to cigarette smoke also face an elevated risk. 2. **Environmental Exposures**    1. Radon Gas: A naturally occurring radioactive gas that can accumulate in homes and buildings. Long-term exposure increases the risk.    2. Asbestos: Often found in workplaces like construction or shipyards, asbestos exposure significantly increases the risk, particularly when combined with smoking.    3. Air Pollution: Fine particulate matter in polluted air can contribute to lung cancer, especially in urban areas. 3. **Personal and Family History**    1. Previous Lung Cancer: Survivors of lung cancer have a higher chance of developing a second primary lung cancer.    2. Family History: Having a close relative with lung cancer may increase susceptibility. 4. **Occupational Hazards**   Exposure to carcinogens like arsenic, chromium, nickel, or diesel exhaust in certain industries increases risk.   1. **Other Medical Conditions**   Chronic Obstructive Pulmonary Disease (COPD), pulmonary fibrosis, or a history of tuberculosis can predispose individuals to lung cancer.   1. **Genetic Predisposition**   Certain inherited genetic mutations may make some individuals more prone to lung cancer, even without significant exposure to risk factors.   1. **Lifestyle Factors**   Low levels of physical activity, poor nutrition, and excessive alcohol consumption might contribute indirectly to the risk.  Understanding these risk factors can help with prevention and early detection efforts. |
| 1. **What are the auxiliary examinations and assessments that should be performed for lung cancer?** | Auxiliary examinations and assessments for lung cancer are crucial for diagnosis, staging, and treatment planning. These include:   1. **Imaging Studies**    1. Chest X-ray: Initial imaging to detect abnormalities.    2. CT Scan: Provides detailed images of the lungs and surrounding structures.    3. PET Scan: Identifies areas of high metabolic activity, often indicating cancer spread.    4. MRI: Used for detecting brain or spinal metastases.    5. Bone Scan: Assesses for bone metastases. 2. **Laboratory Tests**    1. Blood Tests: To check overall health and organ function.    2. Tumor Markers: Such as CEA (Carcinoembryonic Antigen) or CYFRA 21-1, which may be elevated in lung cancer. 3. **Bronchoscopy**   A procedure to visualize the airways and collect tissue samples for biopsy.   1. **Biopsy Techniques**    1. Needle Aspiration: For sampling tissue from the lung or lymph nodes.    2. Surgical Biopsy: For more extensive tissue sampling. 2. **Pulmonary Function Tests (PFTs)**   Assess lung capacity and function, especially important before surgery.   1. **Molecular and Genetic Testing**   Identifies mutations (e.g., EGFR, ALK) or biomarkers (e.g., PD-L1) to guide targeted therapies.  These assessments help in confirming the diagnosis, determining the stage, and tailoring treatment plans. |
| 1. **What is the role of CT scan in the diagnosis of lung cancer?** | A CT (Computed Tomography) scan plays a crucial role in the diagnosis and management of lung cancer. Here's how:   1. **Detection**    1. CT scans provide detailed images of the lungs and surrounding structures, allowing for early detection of abnormalities that might be missed on a standard chest X-ray.    2. They are particularly effective in spotting small nodules or lesions. 2. **Characterization**    1. CT scans help differentiate between benign and potentially malignant lesions by analyzing the size, shape, location, and density of nodules.    2. The scan can also show signs of calcification, which may indicate a benign condition. 3. **Staging**    1. CT scans determine the extent of cancer spread within the chest, including involvement of lymph nodes, pleura, or other nearby organs.    2. This is critical for accurately staging the disease and planning treatment. 4. **Biopsy Guidance**   CT scans are often used to guide fine-needle aspiration or core biopsies, especially for lesions located deep in the lung or near the chest wall.   1. **Follow-up**   They are used to monitor changes in lung nodules over time or assess the effectiveness of treatment in patients with confirmed lung cancer.   1. **Screening in High-Risk Individuals**   Low-dose CT (LDCT) scans are recommended for lung cancer screening in high-risk individuals, such as long-term smokers, as they can detect cancer in its early, more treatable stages.  CT scans are indispensable in the diagnostic process, offering precise imaging to facilitate decision-making. |
| 1. **What is the role of genetic testing in the diagnosis of lung cancer?** | Genetic testing plays a pivotal role in the diagnosis and management of lung cancer by identifying specific genetic mutations or alterations that drive the disease. Here's how it contributes:   1. **Diagnosis and Classification**    1. Genetic testing helps classify lung cancer into subtypes, such as identifying mutations in genes like EGFR, ALK, or KRAS, which are commonly associated with non-small cell lung cancer (NSCLC).    2. It aids in distinguishing between different types of lung cancer, which is crucial for selecting appropriate treatments. 2. **Targeted Therapy**    1. By identifying actionable genetic mutations, genetic testing enables the use of targeted therapies. For example:       1. EGFR mutations can be treated with EGFR inhibitors.       2. ALK rearrangements respond to ALK inhibitors.    2. This personalized approach improves treatment outcomes and reduces unnecessary side effects. 3. **Prognosis**   Certain genetic alterations can provide insights into the aggressiveness of the cancer and its likely progression, helping doctors predict outcomes more accurately.   1. **Screening and Early Detection**   In individuals with a family history of lung cancer, genetic testing can identify inherited mutations that may increase risk, allowing for early screening and preventive measures.   1. **Monitoring and Resistance**   Genetic testing can be used to monitor treatment response and detect the emergence of resistance mutations, guiding adjustments in therapy.   1. **Minimally Invasive Techniques**   Techniques like liquid biopsy analyze circulating tumor DNA (ctDNA) in the blood, offering a less invasive alternative to traditional tissue biopsies for genetic testing.  Genetic testing has revolutionized lung cancer care by enabling precision medicine and improving patient outcomes. |
| **Topic:** **Treatment** | |
| 1. **What are the treatment methods for lung cancer?** | Lung cancer treatment depends on the type, stage, and overall health of the patient. Here are the main methods:   1. **Surgery**    1. Removes the tumor and surrounding tissue.    2. Types include lobectomy (removal of a lung lobe), pneumonectomy (removal of an entire lung), and wedge resection (removal of a small part of the lung). 2. **Radiation Therapy**    1. Uses high-energy rays to kill cancer cells.    2. Can be used alone or in combination with other treatments, especially for inoperable tumors. 3. **Chemotherapy**    1. Involves drugs that kill or stop the growth of cancer cells.    2. Often used for advanced stages or in combination with surgery and radiation. 4. **Targeted Therapy**    1. Focuses on specific genetic mutations or proteins in cancer cells (e.g., EGFR or ALK inhibitors).    2. Effective for certain types of non-small cell lung cancer (NSCLC). 5. **Immunotherapy**    1. Boosts the immune system to recognize and attack cancer cells.    2. Drugs like PD-1/PD-L1 inhibitors are commonly used. 6. **Palliative Care**   Focuses on relieving symptoms and improving quality of life, especially in advanced stages.   1. **Clinical Trials**   Offers access to new and experimental treatments.  Each treatment plan is tailored to the individual. |
| 1. **Is further treatment needed after surgery?** | Yes, further treatment after surgery for lung cancer may be needed, depending on factors like the stage and type of cancer, the presence of residual cancer cells, and the patient’s overall health. Here's an overview of potential post-surgical treatments:   1. **Adjuvant Therapy**    1. Chemotherapy: Often recommended for stages II and III lung cancer to eliminate microscopic cancer cells that may remain after surgery.    2. Radiation Therapy: Used if there are concerns about residual cancer cells, particularly in the lymph nodes or surrounding areas. 2. **Targeted Therapy**   For patients with specific genetic mutations (e.g., EGFR, ALK), targeted drugs may be prescribed to prevent recurrence.   1. **Immunotherapy**   Drugs like PD-1/PD-L1 inhibitors can be given to help the immune system attack any remaining cancer cells, especially in certain types of non-small cell lung cancer (NSCLC).   1. **Regular Monitoring**   Frequent follow-ups with imaging tests (e.g., CT scans) and blood work are essential to detect any signs of recurrence.   1. **Lifestyle Modifications and Support**   Quitting smoking, maintaining a healthy diet, and managing stress can support recovery and reduce the risk of recurrence.  The decision about additional treatment is personalized, taking into account the patient's specific situation. |
| 1. **In what situations is targeted therapy for lung cancer applicable?** | Targeted therapy for lung cancer is applicable in situations where the cancer cells possess specific genetic mutations or molecular markers that can be targeted with specialized drugs. These situations typically include:   1. **Genetic Mutations**    1. EGFR Mutations: Found in many non-small cell lung cancer (NSCLC) patients, particularly adenocarcinomas. Drugs like erlotinib, gefitinib, and osimertinib are used.    2. ALK Rearrangements: Common in younger non-smokers with NSCLC. ALK inhibitors like crizotinib or alectinib can be effective.    3. ROS1 Rearrangements: Another mutation seen in some NSCLC patients; targeted drugs like crizotinib may be beneficial.    4. BRAF Mutations: Patients with BRAF V600E mutations may respond to BRAF inhibitors. 2. **Protein Expression**   PD-L1 Expression: Patients with high PD-L1 levels are eligible for certain immune checkpoint inhibitors, like pembrolizumab, which act as a type of targeted therapy.   1. **MET Amplification or Exon 14 Skipping Mutations**   MET-targeted drugs like tepotinib or capmatinib can be used for NSCLC patients with these genetic abnormalities.   1. **RET Rearrangements**   Patients with RET gene fusions may benefit from RET inhibitors like selpercatinib.   1. **HER2 Mutations**   HER2-targeted therapies may be considered for a small subset of patients with NSCLC.   1. **Combination Therapy**   In advanced stages, targeted therapy may be combined with other treatments, like chemotherapy or immunotherapy, depending on the patient's overall health and cancer progression.   1. **Resistance Development**   Targeted therapy is also applicable in cases where resistance mutations (e.g., T790M mutation in EGFR-positive patients) arise after initial treatment, as second-line targeted drugs can address these changes.   1. **Personalized Treatment Plans**   Patients with advanced or metastatic lung cancer who undergo genetic testing are often considered for targeted therapy if their tumor harbors actionable mutations.  The decision to use targeted therapy depends on molecular and genetic testing results. |
| 1. **How effective is chemotherapy for lung cancer?** | Chemotherapy is a widely used treatment for lung cancer, and its effectiveness depends on several factors, including the type and stage of the cancer, as well as the patient's overall health. Here's an overview:   1. **Small Cell Lung Cancer (SCLC)**    1. Chemotherapy is highly effective for SCLC, especially in the early stages. It is often the primary treatment because this type of cancer tends to grow and spread rapidly.    2. In advanced stages, chemotherapy can help shrink tumors, relieve symptoms, and improve survival rates. 2. **Non-Small Cell Lung Cancer (NSCLC)**    1. Chemotherapy is commonly used in combination with surgery or radiation therapy for NSCLC.    2. In early stages, it may be used as adjuvant therapy (after surgery) to eliminate any remaining cancer cells.    3. In advanced stages, chemotherapy can help control the disease and improve quality of life, though it may not be curative. 3. **Combination with Other Treatments**   Chemotherapy is often combined with targeted therapy or immunotherapy to enhance its effectiveness, particularly in advanced or metastatic lung cancer.   1. **Palliative Benefits**   Even when a cure is not possible, chemotherapy can help reduce symptoms and improve the patient's quality of life.  While chemotherapy has proven benefits, its effectiveness varies from person to person. Side effects can also impact its tolerability, but advances in supportive care have made it more manageable. |
| 1. **In what situations is radiotherapy for lung cancer applicable?** | Radiotherapy, or radiation therapy, is an important treatment for lung cancer and is applicable in various situations, depending on the type and stage of the disease. Here are the key scenarios:   1. **Early-Stage Lung Cancer**   For patients who are not suitable candidates for surgery (due to health conditions or personal choice), stereotactic body radiotherapy (SBRT) is often used. It delivers high doses of precise radiation to small, localized tumors.   1. **Locally Advanced Lung Cancer**    1. In stage III lung cancer, radiotherapy is often combined with chemotherapy (called chemoradiation) to improve treatment outcomes. It may help control the tumor and prevent spread.    2. Postoperative Radiation Therapy (PORT) may be used if residual cancer cells are found after surgery, especially in nearby lymph nodes. 2. **Advanced or Metastatic Lung Cancer**   Radiotherapy is used to relieve symptoms caused by large tumors or metastases. This is called palliative radiotherapy and can help reduce pain, ease breathing, or control bleeding.   1. **Brain Metastases**   Lung cancer, particularly small cell lung cancer (SCLC), often spreads to the brain. Whole-brain radiotherapy (WBRT) or stereotactic radiosurgery (SRS) is used to treat brain metastases.   1. **Prophylactic Cranial Irradiation (PCI)**   In patients with small cell lung cancer, PCI is sometimes used to prevent cancer from spreading to the brain, even if no metastases are currently detected.   1. **Superior Vena Cava Syndrome**   For tumors compressing the superior vena cava (a major vein in the chest), radiotherapy can relieve symptoms like swelling of the face and neck.   1. **Residual or Recurrent Disease**   Radiation may be applied to areas where cancer persists or recurs after initial treatments.  Radiotherapy is tailored to each patient’s unique situation, balancing effectiveness with potential side effects. |
| 1. **What is immunotherapy?** | Immunotherapy is a type of treatment that harnesses the power of the body's immune system to fight diseases, including cancer. Instead of directly targeting cancer cells (like chemotherapy or radiation), immunotherapy works by either boosting the immune system's natural ability to detect and destroy cancer cells or by modifying immune responses to attack cancer more effectively.  **Types of Immunotherapy for Lung Cancer:**   1. **Immune Checkpoint Inhibitors:**   These drugs block proteins that prevent immune cells (like T-cells) from attacking cancer. Common targets include:   - - 1. PD-1/PD-L1 inhibitors (e.g., pembrolizumab, nivolumab) help the immune system recognize and attack lung cancer cells.     2. CTLA-4 inhibitors (e.g., ipilimumab) enhance T-cell activity against cancer.  1. **Cancer Vaccines:**   Vaccines stimulate the immune system to attack cancer. While not widely used yet, they are being developed and tested for lung cancer.   1. **Monoclonal Antibodies:**   These lab-made antibodies can flag cancer cells for destruction by the immune system or block signals that help tumors grow.   1. **Cytokine Therapy:**   Cytokines are proteins that regulate immune responses. Interleukins or interferons are used to boost immune system activity.   1. **Adoptive Cell Transfer:**   A personalized therapy where immune cells are removed, modified to better attack cancer, and then reintroduced into the body.  **Benefits:**   1. Immunotherapy can be highly effective, especially for certain lung cancer patients with high levels of PD-L1 expression or specific genetic features. 2. It often has fewer side effects compared to traditional treatments like chemotherapy.   **Limitations:**   1. It doesn't work for all patients, as some tumors have ways of evading the immune system. 2. Response to immunotherapy may take time and varies between individuals.   Immunotherapy is a groundbreaking advancement in cancer treatment, offering hope to patients with advanced or resistant lung cancer. |
| **Topic:** **Prognosis** | |
| 1. **What is the frequency of follow-up after lung cancer surgery?** | The frequency of follow-up after lung cancer surgery depends on the type and stage of cancer, as well as the treatment received. Here are general guidelines:  **For Non-Small Cell Lung Cancer (NSCLC):**   1. **Stage I or II (after surgery with or without chemotherapy):**     1. Physical exams and chest CT scans every 6 months for the first 2–3 years.    2. Annually starting from the 4th year. 2. **Stage III or IV:**     1. Physical exams and chest CT scans every 3–6 months for the first 3 years.    2. Every 6 months for the next 2 years.    3. Annually after 5 years.   **For Small Cell Lung Cancer (SCLC):**   1. **Limited-Stage:**     1. Follow-up every 3 months for the first 2 years.    2. Every 6 months in the 3rd year.    3. Annually after that.    4. Brain MRI every 3–4 months in the first year, then every 6 months in the second year. 2. **Extensive-Stage:**     1. Follow-up every 2 months in the first year.    2. Every 3–4 months in years 2 and 3.    3. Every 6 months in years 4 and 5.    4. Annually after 5 years.   These schedules may vary based on individual circumstances, so it's essential to follow your healthcare provider's recommendations. |
| 1. **What should be done if a new nodule is found during follow-up?** | If a new nodule is found during follow-up after lung cancer treatment, the approach depends on its characteristics and the patient's medical history. Here's a general outline of what might be done:   1. **Assessment of Nodule Characteristics**    1. Size: Larger nodules are more concerning and may require immediate attention.    2. Shape and Density: Irregular or spiculated nodules are more likely to be malignant.    3. Growth Rate: Comparing the new nodule to previous scans helps determine if it's growing rapidly. 2. **Risk Evaluation**   Factors like smoking history, age, and previous cancer type are considered to assess the likelihood of malignancy.   1. **Imaging Studies**    1. CT Scan: A high-resolution CT scan is often the first step to get a detailed view of the nodule.    2. PET Scan: May be used to assess metabolic activity, which can indicate malignancy. 2. **Biopsy or Sampling**   If the nodule appears suspicious, a biopsy (via bronchoscopy or needle aspiration) may be performed to confirm the diagnosis.   1. **Monitoring**   For small or low-risk nodules, periodic imaging (e.g., follow-up CT scans) may be recommended to monitor changes over time.   1. **Treatment Planning**   If the nodule is confirmed to be malignant, treatment options like surgery, radiation, or systemic therapies (e.g., chemotherapy, targeted therapy) will be considered.  It's essential to consult with a healthcare provider for a personalized evaluation and management plan. |
| 1. **How to determine whether lung cancer recurs during follow-up?** | Determining whether lung cancer has recurred during follow-up involves a combination of clinical evaluation, imaging studies, and sometimes laboratory tests. Here's how it is typically done:   1. **Clinical Evaluation**    1. Monitoring for symptoms such as persistent cough, chest pain, shortness of breath, or unexplained weight loss.    2. Regular physical examinations to check for any abnormalities. 2. **Imaging Studies**    1. Chest CT Scans: The most common imaging tool for detecting recurrence. These are performed at regular intervals based on the follow-up schedule.    2. PET Scans: Used to identify areas of high metabolic activity, which may indicate cancer recurrence.    3. MRI: Often used if there is suspicion of brain metastases. 3. **Laboratory Tests**   Tumor Markers: Blood tests for markers like CEA (Carcinoembryonic Antigen) or CYFRA 21-1 may help detect recurrence in some cases.   1. **Biopsy**   If imaging reveals a suspicious lesion, a biopsy may be performed to confirm whether it is cancerous.   1. **Comparison with Previous Records**   Comparing new imaging results with previous scans to detect changes in size, shape, or location of nodules or masses.   1. **Patient-Reported Symptoms**   Patients are encouraged to report any new or worsening symptoms promptly, as these can be early signs of recurrence.  Regular follow-up appointments and adherence to the recommended surveillance schedule are crucial for early detection of recurrence. |
| 1. **How to manage lifestyle after lung cancer surgery?** | Managing your lifestyle after lung cancer surgery is crucial for recovery and long-term health. Here are some key areas to focus on:   1. **Physical Recovery**    1. Breathing Exercises: Practice deep breathing and use incentive spirometry to improve lung function.    2. Gradual Activity: Start with light activities like walking and gradually increase intensity as your strength improves.    3. Pain Management: Follow your doctor's advice on pain relief, and don't hesitate to report any discomfort. 2. **Healthy Diet**    1. Focus on a balanced diet rich in fruits, vegetables, lean proteins, and whole grains to support healing.    2. Stay hydrated and avoid processed or high-sugar foods. 3. **Smoking Cessation**   If you smoked before surgery, quitting is essential to prevent recurrence and improve lung health.   1. **Emotional and Psychological Support**    1. It's normal to feel anxious or depressed after surgery. Consider joining a support group or seeking counseling.    2. Stay connected with loved ones for emotional support. 2. **Follow-Up Care**   Attend all scheduled follow-up appointments and imaging tests to monitor your recovery and detect any signs of recurrence early.   1. **Avoid Environmental Risks**   Minimize exposure to air pollution, secondhand smoke, and other lung irritants.   1. **Lifestyle Adjustments**    1. Incorporate regular, moderate exercise into your routine to maintain overall health.    2. Get adequate rest and manage stress through relaxation techniques like meditation or yoga.   Recovery is a gradual process, and it's important to listen to your body and communicate with your healthcare team. |
| 1. **Whether the focus of follow-up for long-term survivors (e.g., more than 5 years after surgery) needs to be adjusted?** | Yes, the focus of follow-up for long-term lung cancer survivors often shifts as time passes, particularly for those who are more than 5 years post-surgery. Here's how it typically evolves:   1. **Cancer Recurrence Monitoring**    1. In the early years, follow-up primarily focuses on detecting recurrence through regular imaging (e.g., CT scans) and physical exams.    2. After 5 years, the risk of recurrence decreases significantly, so the frequency of imaging may be reduced, and the focus may shift to monitoring for new primary cancers. 2. **Management of Long-Term Side Effects**   Long-term survivors may experience side effects from surgery, chemotherapy, or radiation, such as reduced lung function, fatigue, or neuropathy. Follow-up care addresses these issues to improve quality of life.   1. **Screening for Secondary Cancers**   Survivors are at an increased risk of developing other cancers, especially if they have a history of smoking. Regular screenings for cancers like colorectal, breast, or prostate cancer may become a priority.   1. **Cardiovascular Health**   Treatments like chemotherapy and radiation can increase the risk of heart disease. Long-term follow-up may include monitoring cardiovascular health and managing risk factors like hypertension or cholesterol.   1. **Psychosocial Support**   Emotional and psychological well-being remains important. Survivors may need ongoing support to cope with anxiety, depression, or fear of recurrence.   1. **Lifestyle and Preventive Care**   Emphasis on maintaining a healthy lifestyle, including smoking cessation, regular exercise, and a balanced diet, becomes a key focus to reduce the risk of other health issues.   1. **Individualized Care Plans**   Follow-up care is tailored to the survivor's unique needs, taking into account their medical history, treatment side effects, and personal risk factors.  The goal of long-term follow-up is to ensure overall health and well-being while addressing any late effects of treatment. |
| 1. **How to manage long-term side effects after treatment, such as lung function damage, radiation pneumonitis, etc.** | Managing long-term side effects after lung cancer treatment, such as lung function damage and radiation pneumonitis, requires a combination of medical care, lifestyle adjustments, and supportive therapies. Here's a guide:   1. **Lung Function Damage**    1. Pulmonary Rehabilitation: Engage in a supervised program that includes breathing exercises, physical activity, and education to improve lung capacity.    2. Medications: Bronchodilators or corticosteroids may help reduce inflammation and improve airflow.    3. Oxygen Therapy: For severe cases, supplemental oxygen can assist with breathing.    4. Avoid Lung Irritants: Minimize exposure to smoke, pollution, and allergens. 2. **Radiation Pneumonitis**    1. Medications: Corticosteroids like prednisone are often prescribed to reduce inflammation in the lungs.    2. Monitoring: Regular follow-ups with imaging (e.g., CT scans) to assess lung health.    3. Symptom Management: Use humidifiers and stay hydrated to ease symptoms like coughing. 3. **Fatigue**    1. Energy Conservation: Plan activities to avoid overexertion and prioritize rest.    2. Exercise: Light to moderate physical activity can help combat fatigue over time. 4. **Emotional and Psychological Support**    1. Counseling: Seek therapy or join support groups to address anxiety, depression, or fear of recurrence.    2. Mindfulness Practices: Techniques like meditation or yoga can help manage stress. 5. **Nutrition**   Maintain a balanced diet rich in antioxidants, vitamins, and minerals to support overall health and recovery.   1. **Regular Medical Follow-Ups**   Attend all scheduled appointments to monitor for late effects and manage any new symptoms promptly.   1. **Lifestyle Adjustments**    1. Quit smoking if applicable, and avoid secondhand smoke.    2. Stay physically active and maintain a healthy weight.   Each individual's recovery journey is unique, so it's essential to work closely with your healthcare team to tailor a plan that meets your specific needs. |
| **Topic:** **Case** | |
| 1. **A 68-year-old male patient, with a 40-year history of smoking, has been experiencing cough and blood-tinged sputum for 3 months. Chest X-ray showed an irregular mass in the right upper lung with a rough edge; CT scan suggested that the mass invaded the pleura and mediastinal lymph nodes were enlarged. Lung cancer is initially considered, and bronchoscopy and biopsy are recommended to confirm the pathology. What further tests does the patient need to confirm the diagnosis?** | In addition to bronchoscopy and biopsy, the following tests may be recommended to confirm the diagnosis and assess the extent of lung cancer:   1. **Imaging Studies**    1. PET-CT Scan: Combines positron emission tomography (PET) and computed tomography (CT) to detect areas of high metabolic activity, which may indicate cancer spread.    2. MRI: Particularly useful for evaluating potential brain metastases or spinal involvement.    3. Bone Scan: Helps identify cancer spread to the bones. 2. **Endobronchial Ultrasound (EBUS)**   A specialized bronchoscopy technique that uses ultrasound to guide needle aspiration of lymph nodes or masses near the airways.   1. **Mediastinoscopy**   A surgical procedure to sample lymph nodes in the mediastinum for pathological examination.   1. **Molecular and Genetic Testing**   Identifies specific mutations (e.g., EGFR, ALK, KRAS) or biomarkers (e.g., PD-L1) to guide targeted therapy decisions.   1. **Blood Tests**    1. Tumor Markers: Such as CEA (Carcinoembryonic Antigen) or CYFRA 21-1, which may be elevated in lung cancer.    2. General blood tests to assess overall health and organ function. 2. **Thoracoscopy or Thoracentesis**   If pleural effusion is present, thoracentesis can be performed to analyze the fluid for cancer cells. Thoracoscopy allows direct visualization and biopsy of the pleura.  These tests help confirm the diagnosis, determine the stage of the cancer, and guide treatment planning. |
| 1. **A 55-year-old female patient, diagnosed with advanced non-small cell lung cancer (NSCLC), with EGFR gene mutation positive. She has received gefitinib targeted therapy, but recently developed drug resistance and tumor progression. How to adjust the next treatment plan?** | When a patient with advanced NSCLC and EGFR-positive mutation develops resistance to gefitinib and experiences tumor progression, the treatment plan can be adjusted as follows:   1. **Switch to a Third-Generation EGFR-TKI**   Osimertinib: This is often the next step if the patient has developed the T790M resistance mutation, which is a common mechanism of acquired resistance to first-generation EGFR-TKIs like gefitinib.   1. **Combination Therapy**    1. Chemotherapy: Platinum-based chemotherapy (e.g., carboplatin or cisplatin combined with pemetrexed) may be considered if targeted therapy is no longer effective.    2. Anti-VEGF Therapy: Drugs like bevacizumab can be combined with chemotherapy to enhance efficacy. 2. **Molecular Testing**   Conduct further molecular testing to identify additional mutations or alterations (e.g., MET amplification, HER2 mutations) that could guide alternative targeted therapies.   1. **Immunotherapy**   If the tumor expresses high levels of PD-L1, immune checkpoint inhibitors like pembrolizumab may be considered.   1. **Clinical Trials**   Explore ongoing clinical trials for novel therapies targeting EGFR resistance mechanisms or other actionable mutations.   1. **Local Therapy**   For isolated progression (e.g., brain or bone metastases), localized treatments like stereotactic radiosurgery or radiation therapy can be used while continuing systemic therapy.  The choice of treatment depends on the patient's overall health, specific resistance mechanisms, and tumor characteristics. |
| 1. **A 70-year-old male patient, on the 3rd day after lung cancer surgery, developed pleural effusion and poor drainage. Body temperature was 37.8°C, heart rate was 90 beats/min, respiratory rate was 22 breaths/min, and the drainage tube was blocked. The patient complained of chest tightness. How to deal with the blocked drainage tube?** | Managing a blocked drainage tube after lung cancer surgery is critical to prevent complications like pleural effusion buildup or infection. Here's how it can be addressed:   1. **Immediate Assessment**    1. Check for Blockage: Inspect the drainage tube for kinks, clots, or obstructions. Ensure the tube is properly positioned and not compressed externally.    2. Assess the Patient's Condition: Monitor vital signs and symptoms, such as chest tightness, fever, or respiratory distress, to determine the urgency of intervention. 2. **Intervention**    1. Flush the Tube: A sterile saline flush can be performed to clear minor blockages, but this should only be done by trained medical personnel.    2. Replace the Tube: If the blockage cannot be resolved, the drainage tube may need to be replaced under sterile conditions.    3. Thoracentesis: If pleural effusion persists and the tube remains ineffective, a thoracentesis (needle aspiration of fluid) may be performed. 3. **Supportive Measures**    1. Antibiotics: If infection is suspected (e.g., fever or elevated white blood cell count), antibiotics may be initiated.    2. Pain Management: Address any discomfort caused by the blocked tube or associated procedures. 4. **Monitoring**    1. Regularly check the drainage system for proper function and ensure the patient is stable.    2. Perform follow-up imaging (e.g., chest X-ray or ultrasound) to evaluate the resolution of pleural effusion.   This situation requires prompt medical attention, and all interventions should be carried out by healthcare professionals. |
| 1. **A 48-year-old female patient, newly diagnosed with advanced lung cancer, is feeling low and refusing treatment. The patient has obvious anxiety and depression, worrying about the side effects of treatment and family burden. How to help the patient rebuild treatment confidence?** | Helping a patient rebuild treatment confidence requires a compassionate and holistic approach. Here are some strategies:   1. **Address Emotional Concerns**    1. Counseling: Encourage the patient to speak with a psychologist or counselor specializing in oncology to address anxiety and depression.    2. Support Groups: Introduce her to lung cancer support groups where she can connect with others who have faced similar challenges. 2. **Provide Clear Information**    1. Explain Treatment Options: Offer detailed yet simple explanations about the benefits and side effects of treatment, emphasizing advancements that minimize discomfort.    2. Highlight Success Stories: Share examples of patients who have successfully managed advanced lung cancer with treatment. 3. **Involve Family**    1. Family Meetings: Include family members in discussions to provide emotional support and alleviate her concerns about being a burden.    2. Delegate Responsibilities: Help her family create a plan to share caregiving duties. 4. **Focus on Quality of Life**    1. Palliative Care: Reassure her that treatments can be tailored to improve her quality of life, even if the goal is not curative.    2. Symptom Management: Emphasize that side effects can be managed effectively with modern medicine. 5. **Explore Financial Support**   Financial Counseling: Connect her with resources or organizations that can assist with treatment costs to reduce financial stress.   1. **Build Trust with the Medical Team**   Regular Communication: Encourage open dialogue with her healthcare team to address fears and build confidence in the treatment plan.   1. **Consider Professional Help**   If her refusal persists, a psychiatrist may evaluate her for clinical depression and recommend appropriate interventions.  Empathy and patience are key to helping her regain confidence. |
| 1. **A 65-year-old male patient, diagnosed with locally advanced non-small cell lung cancer, is not suitable for surgical resection. He has completed concurrent chemoradiotherapy, but developed radiation pneumonitis and shortness of breath. How to relieve the symptoms of radiation pneumonitis?** | Radiation pneumonitis is a common side effect of thoracic radiation therapy, and managing its symptoms involves a combination of medical treatments and supportive care. Here's how the symptoms can be relieved:   1. **Medications**    1. Corticosteroids: Prednisone is commonly prescribed to reduce lung inflammation. The dosage is typically tapered over weeks based on the patient's response.    2. Bronchodilators: These can help open the airways and improve breathing.    3. Antibiotics: If there is a suspicion of infection, antibiotics may be added to the treatment plan. 2. **Oxygen Therapy**   Supplemental oxygen may be provided to alleviate shortness of breath and improve oxygen levels in the blood.   1. **Symptom Management**    1. Cough Suppressants: To reduce persistent coughing.    2. Pain Relievers: If chest pain is present, appropriate pain management can be provided. 2. **Lifestyle Adjustments**    1. Encourage the patient to avoid lung irritants such as smoke, dust, and strong odors.    2. Maintain hydration and use a humidifier to ease breathing. 3. **Monitoring and Follow-Up**    1. Regular follow-up with imaging (e.g., chest X-rays or CT scans) to monitor the progression or resolution of pneumonitis.    2. Pulmonary function tests may be conducted to assess lung capacity and recovery. 4. **Pulmonary Rehabilitation**   A supervised program that includes breathing exercises and physical activity to improve lung function and overall well-being.  These interventions should be tailored to the patient's specific condition and severity of symptoms. Close communication with the healthcare team is essential to ensure effective management. |

**Supplementary Table 3.** Consistency Analysis of Scores Given by Three Experts

|  | Number | ICC single | P value | ICC average | P value | Cronbach's α |
| --- | --- | --- | --- | --- | --- | --- |
| **All questions** | 29 | 0.844 | ＜0.001 | 0.942 | ＜0.001 | 0.942 |
| Different aspects |  |  |  |  |  |  |
| Basic | 6 | 0.835 | ＜0.001 | 0.938 | ＜0.001 | 0.938 |
| Diagnosis | 6 | 0.856 | ＜0.001 | 0.947 | ＜0.001 | 0.947 |
| Treatment | 6 | 0.822 | ＜0.001 | 0.933 | ＜0.001 | 0.933 |
| Prognosis | 6 | 0.857 | ＜0.001 | 0.947 | ＜0.001 | 0.947 |
| Case | 5 | 0.851 | ＜0.001 | 0.945 | ＜0.001 | 0.945 |
| Different dimensions |  |  |  |  |  |  |
| Accuracy | 29 | 0.553 | ＜0.001 | 0.788 | ＜0.001 | 0.788 |
| Comprehensiveness | 29 | 0.727 | ＜0.001 | 0.889 | ＜0.001 | 0.889 |
| Understandability | 29 | 0.687 | ＜0.001 | 0.868 | ＜0.001 | 0.868 |
| Reliability | 29 | 0.586 | ＜0.001 | 0.809 | ＜0.001 | 0.809 |
| Humanistic care | 29 | 0.382 | ＜0.001 | 0.650 | ＜0.001 | 0.650 |

**Supplementary Table 4.** Post-hoc Analysis Across Different Dimensions

| Different dimensions | LLM | Difference in means | SE | p value |
| --- | --- | --- | --- | --- |
| Accuracy | ChatGPT VS Claude | -0.379 | 0.155 | 0.016 |
|  | ChatGPT VS Coplit | 0.368 | 0.155 | 0.019 |
|  | Claude VS Coplit | 0.747 | 0.155 | ＜0.001 |
|  | Claude VS Gemini | 0.333 | 0.155 | 0.034 |
|  | Coplit VS Gemini | -0.414 | 0.155 | 0.009 |
| Comprehensiveness | ChatGPT VS Claude | -0.586 | 0.218 | 0.008 |
|  | ChatGPT VS Coplit | 0.448 | 0.218 | 0.042 |
|  | Claude VS Coplit | 1.034 | 0.218 | ＜0.001 |
|  | Coplit VS Gemini | -0.678 | 0.218 | 0.002 |
| Understandability | ChatGPT VS Claude | 0.459 | 0.201 | 0.024 |
|  | ChatGPT VS Gemini | -0.403 | 0.201 | 0.047 |
|  | Claude VS Coplit | -0.805 | 0.201 | ＜0.001 |
|  | Claude VS Gemini | -0.862 | 0.201 | ＜0.001 |
| Reliability | ChatGPT VS Coplit | 0.346 | 0.157 | 0.030 |
|  | Claude VS Coplit | 0.540 | 0.157 | 0.001 |
|  | Coplit VS Gemini | -0.322 | 0.157 | 0.043 |

**Supplementary Table 5.** Post-hoc Analysis Across Different Aspects

| Different aspects | LLM | Difference in means | SE | p value |
| --- | --- | --- | --- | --- |
| Basic | Claude VS Coplit | 0.767 | 0.282 | 0.008 |
|  | Claude VS Gemini | 0.609 | 0.282 | 0.033 |
